# Computationally guided discovery of a reactive, hydrophilic *trans*-5-oxocene dienophile for bioorthogonal labeling†
†Electronic supplementary information (ESI) available. See DOI: 10.1039/c7ob01707c


**DOI:** 10.1039/c7ob01707c

**Published:** 2017-07-20

**Authors:** William D. Lambert, Samuel L. Scinto, Olga Dmitrenko, Samantha J. Boyd, Ronald Magboo, Ryan A. Mehl, Jason W. Chin, Joseph M. Fox, Stephen Wallace

**Affiliations:** a Brown Laboratory , Department of Chemistry & Biochemistry , University of Delaware , Newark , Delaware 19716 , USA . Email: jmfox@udel.edu; b Lotus Separations LLC , Newark , DE 19711 , USA; c Department of Biochemistry and Biophysics , Oregon State University , Corvallis , Oregon 97331 , USA; d Medical Research Council Laboratory of Molecular Biology , Francis Crick Avenue , Cambridge Biomedical Campus , Cambridge CB2 0QH , UK; e Institute of Quantitative Biology , Biochemistry and Biotechnology , School of Biological Sciences , University of Edinburgh , UK

## Abstract

The use of organic chemistry principles and prediction techniques has enabled the development of new bioorthogonal reactions. As this “toolbox” expands to include new reaction manifolds and orthogonal reaction pairings, the continued development of existing reactions remains an important objective. This is particularly important in cellular imaging, where non-specific background fluorescence has been linked to the hydrophobicity of the bioorthogonal moiety. Here we report that *trans*-5-oxocene (oxoTCO) displays enhanced reactivity and hydrophilicity compared to *trans*-cyclooctene (TCO) in the tetrazine ligation reaction. Aided by *ab initio* calculations we show that the insertion of a single oxygen atom into the *trans*-cyclooctene (TCO) ring system is sufficient to impart aqueous solubility and also results in significant rate acceleration by increasing angle strain. We demonstrate the rapid and quantitative cycloaddition of oxoTCO using a water-soluble tetrazine derivative and a protein substrate containing a site-specific genetically encoded tetrazine moiety both *in vitro* and *in vivo*. We anticipate that oxoTCO will find use in studies where hydrophilicity and fast bioconjugation kinetics are paramount.

## Abstract 2

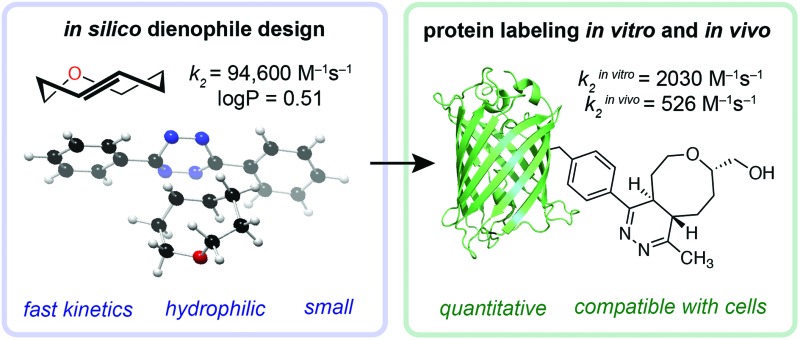
The use of organic chemistry principles and prediction techniques has enabled the development of new bioorthogonal reactions.

## Introduction

**Fig. 1:**
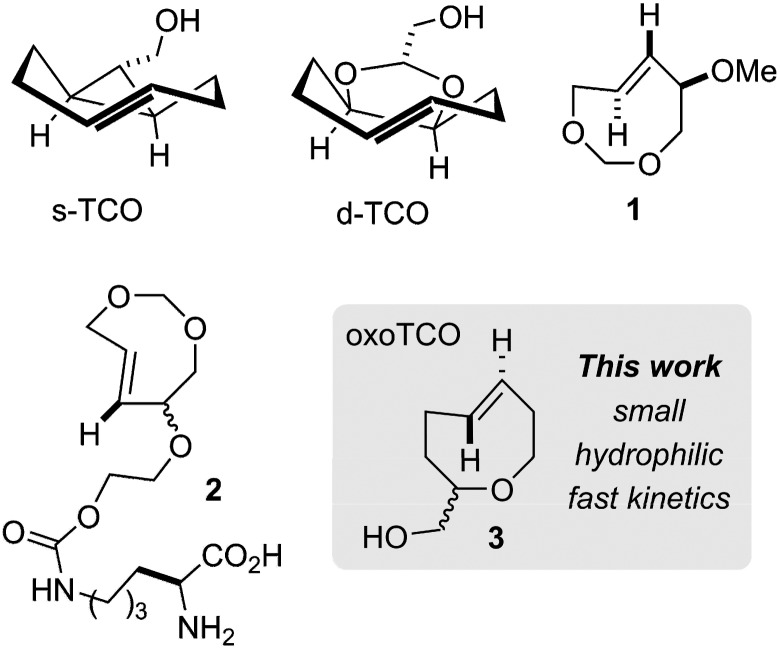
Conformationally strained and heterocyclic *trans*-cyclooctenes.

Biotechnology and biomedicine have been profoundly influenced by the development of new bioorthogonal reactions – abiotic transformations that occur selectively in a biological environment.^1^–^9^ Amongst these, the cycloaddition of alkenes/alkynes and *s*-tetrazines has become an important member of the bioorthogonal reaction “toolbox”.^10^–^20^ Since initial reports using *trans*-cyclooctene (TCO)^21^ and norbornene derivatives,^22^ a complementary range of dienophiles has been developed – including cyclopropenes,^23^,^24^ cyclooctynes^25^,^26^ and simple α-olefins.^27^–^29^ However, *trans*-cyclooctene (TCO) still maintains the advantage of exceptional reaction kinetics in this process.^3^,^10^ For example, the cycloaddition of the equatorial diastereomer of 5-hydroxy-*trans*-cyclooctene and a 3,6-dipyridyl-*s*-tetrazine derivative occurs with a second-order rate constant of 22 600 M^–1^ s^–1^ in H_2_O at 25 °C.^30^ Faster reactivity can be realized by using the axial diastereomer of 5-hydroxy-*trans*-cyclooctene (80 200 M^–1^ s^–1)^.^11^,^30^ However, the fastest bioorthogonal reactions described to date use the conformationally strained dienophiles s-TCO and d-TCO (Fig. 1).^30^,^31^ These bicyclic molecules adopt a half-chair conformation that is 5.6–5.9 kcal mol^–1^ higher in energy than the crown conformation of monocyclic TCO. Cycloaddition of these compounds with tetrazines display second-order rate constants of up to 366 000 M^–1^ s^–1^ for d-TCO and 3 300 000 M^–1^ s^–1^ for s-TCO.

From a kinetic standpoint, *trans*-cyclooctene derivatives are excellent dienophiles for studies where high reactivity is essential such as in cellular imaging and nuclear medicine.^32^,^33^ However, the hydrophobicity of TCO and s-TCO has recently been linked to high levels of non-specific background fluorescence during imaging experiments, necessitating lengthy washout protocols (>2 h) to dissociate the excess reagent from the cell.^34^,^35^ While d-TCO displays reduced lipophilicity, the compound is relatively bulky compared to the parent TCO system. The development of new, low molecular weight dienophiles for the tetrazine ligation reaction that are fast and hydrophilic is therefore an important challenge.

In seminal work, Jendrella synthesized 4,6-dioxo-TCO **1** and showed it to be 20–1000 fold faster than *trans*-cyclooctene in cycloadditions with cyclopentadiene, 2,3-dimethylbutadiene, mesitonitriloxide and diphenylketene.^36^ More recently, Dudley, Alabugin and coworkers have shown (*in silico*) that 3-oxocyclooctynes display fast reactivity in cycloadditions with azides, and have attributed their fast reactivity partly to the hyperconjugative effect of the allylic oxygens.^37^ Tomooka and Woerpel have synthesized *trans*-oxasilacycloalkenes, and have studied their reactivity in Diels–Alder and azide cycloadditions.^38^,^39^ Very recently, Lemke, Kele and coworkers reported the genetic incorporation of dioxo-TCO **2** and demonstrated that the lower lipophilicity of this molecule resulted in improved washout times during imaging experiments. In Diels–Alder reactions with tetrazines, the reaction rate with **2** is similar to that with the parent TCO.^36^,^40^

In the course of our synthetic studies on transannulations of *cis*- and *trans*-5-oxocenes, we queried whether such com-pounds would engage in rapid bioconjugation reactions.^41^,^42^ Here we report the computational design and synthesis of a *trans*-5-oxocene (“oxoTCO”, **3**) – a small, hydrophilic, and highly reactive dienophile for use in the bioorthogonal tetrazine ligation reaction. The reaction of **3** (2.2 : 1 dr) with a water-soluble 3,6-dipyridyl-*s*-tetrazine-mono-succinamic acid **10** occurs with a second order rate constant of 94 600 M^–1^ s^–1^ in PBS at 25 °C (Fig. 3), which is faster than either diastereomer of 5-hydroxy-*trans*-cyclooctene, and approaching the rate of bicyclic d-TCO. The oxoTCO heterocycle can be synthesized in seven high yielding steps from commercially available glycidol. Furthermore, oxoTCO **3** is small (MW 142) and hydrophilic with an experimental log *P* = 0.51. Finally, we describe the *in vitro* and *in vivo* kinetics of **3** on a recombinant protein substrate containing a site-specifically incorporated tetrazine-containing amino acid (*sf*GFP-150Tet-v.2.0).^43^ We anticipate that oxoTCO **3** will find applications in cellular imaging studies where small hydrophilic probes with fast reaction kinetics, low background fluorescence and/or rapid data acquisition are required.

## Results and discussion

**Fig. 2:**
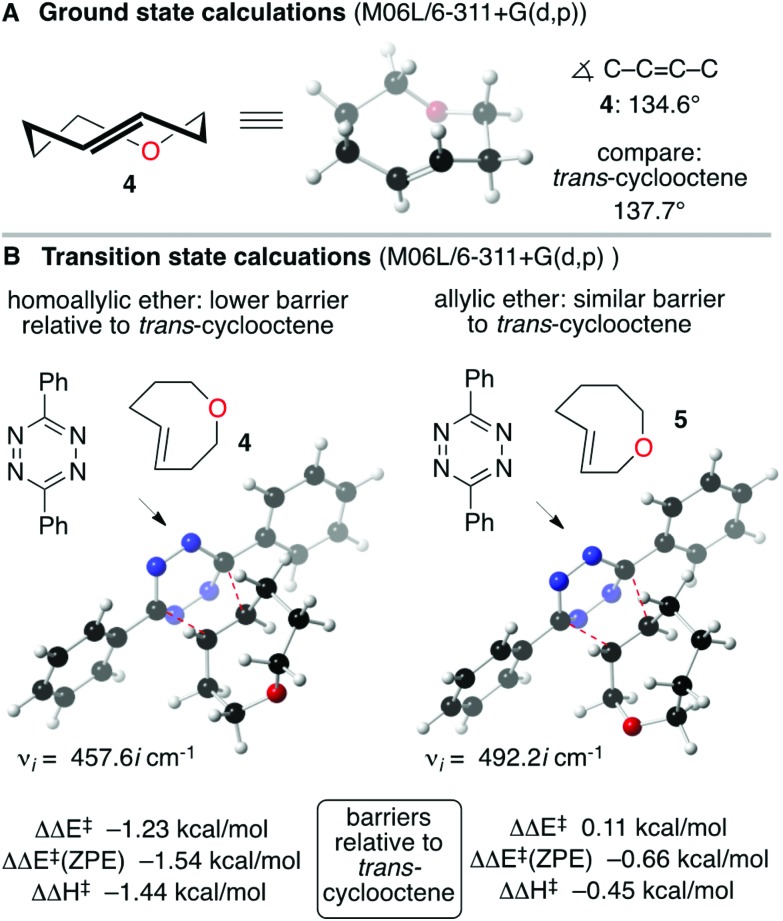
DFT transition state calculations predict that *trans*-5-oxocene **4**, but not *trans*-4-oxocene **5**, would be more reactive than trans-cyclooctene. While both **4** and **5** are more strained than *trans*-cyclooctene, that the reactivity of **5** is attenuated by the electron withdrawing allylic oxygen.

Computation was used to assist the design of a reactive and soluble *trans*-oxocene dienophile. We reasoned that the short C–O bonds in the backbone of a *trans*-5-oxocene would augment the olefinic strain of the *trans*-cycloalkene, and thereby increase the reactivity in tetrazine ligation. As shown in Fig. 2, ground state calculations were carried out for the parent *trans*-oxocenes **4** and **5** as well as *trans*-cyclooctene at the M06L/6-311+G(d,p) level. Indeed, the calculated C–C=C–C dihedral angle for **4** (134.6°) and **5** (134.4°) is significantly shorter than that for *trans*-cyclooctene (137.7°). M06L/6-311+G(d,p) and CAM-B3LYP/tzvp calculations were also carried out to compare the reactivity of **4** and **5** to *trans*-cyclooctene (Fig. 2B). These calculations were carried out with diphenyl-*s*-tetrazine so that they could be benchmarked against previous calculations carried our in our labs.^30^,^31^ At the M06L/6-311+G(d,p) level, the barrier for the Diels–Alder reaction of *trans*-cyclooctene with 3,6-diphenyl-*s*-tetrazine is ΔΔ*E*^‡^ 13.3 kcal mol^–1^, Δ*E*^‡^(ZPE) 13.9 kcal mol^–1^, Δ*H*^‡^ 12.9 kcal mol^–1^. With *trans*-5-oxocene **4**, the barrier was significantly lower, with ΔΔ*E*^‡^ –1.23 kcal mol^–1^, Δ*E*^‡^(ZPE) –1.54 kcal mol^–1^ and Δ*H*^‡^ –1.44 kcal mol^–1^ relative to *trans*-cyclooctene. Interestingly, the isomeric *trans*-4-oxocene **5** is not predicted to be significantly more reactive than *trans*-cyclooctene. This computational result can be rationalized by considering the electron withdrawing nature of the allylic oxygen. Inverse electron demand Diels–Alder reactions are deactivated by electron withdrawing groups on the alkene, and the allylic oxygen of **5** is both inductively withdrawing and stereoelectronically positioned to deactivate the alkene through hyperconjugation. Thus, while the alkene of **5** is more strained than **4** (134.4° *vs.* 134.6° dihedral angle), the effect is attenuated by the electron withdrawing effect of the allylic oxygen.

**Scheme 1:**
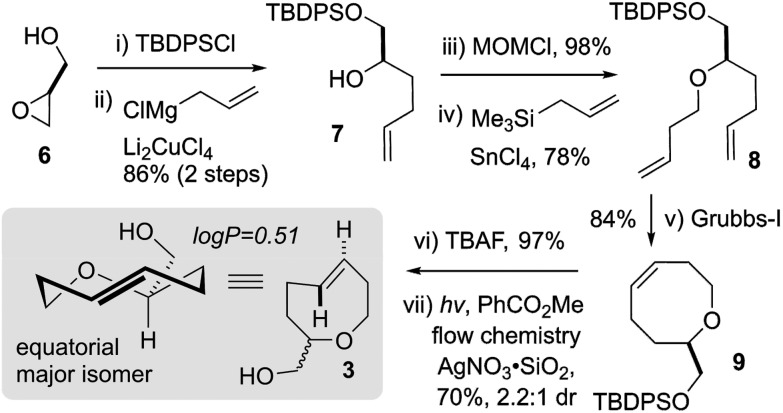
Synthesis of oxoTCO **3**.

Based on these computational predictions, we synthesized the alcohol-functionalized *trans*-5-oxocene **3** in 7 steps from commercially available glycidol **6** (Scheme 1). The synthesis began with TBS-protection and the addition of allyl magnesium chloride to provide alcohol **7**. Our attempts to access **8** directly from **7***via* Williamson etherification or Mitsunobu chemistry were unsuccessful. Fortunately, we found that the treatment of the MOM ether of **7** with Lewis acidic stannic chloride generated a putative oxocarbenium ion that could be quenched *via* Sakurai allylation to afford butenyl ether **8** in 78% yield. Ring-closing metathesis of **8** using the Grubbs first-generation catalyst proceeded efficiently to afford *cis*-oxocene **9** in 84% yield. Finally, desilylation and photoisomerization using our closed-loop flow reactor^44^ afforded a 2.2 : 1 diastereoisomeric mixture of *trans*-oxocenes in 70% yield (37% overall yield over 7 steps). Separation of the diastereomers using preparative thin layer or silica gel chromatography was unsuccessful. An analytical sample of the major diastereomer of **3** was obtained by preparative supercritical fluid chromatography, however, given the difficulty of separation we continued the majority of further studies on oxoTCO using a 2.2 : 1 mixture of diastereomers. The log *P* of **3** was experimentally determined to be 0.51 whereas equatorial 5-hydroxy-*trans*-cyclooctene and d-TCO were both determined to be more hydrophobic with log *P* = 1.11 and 0.94, respectively.^30^

The stability of oxoTCO **3** was studied under several conditions by ^1^H NMR spectroscopy. Over a 14 day period at room temperature, a 2.2 : 1 diastereoisomeric mixture of oxoTCO **3** (33 mM) showed no degradation in CD_3_OD. In D_2_O-PBS (pD = 7.4), the major, equatorial diastereomer of **3** showed less than 10% degradation after 1 week. The more reactive minor diastereomer degraded more rapidly in PBS, and decomposed with a half-life of 36 hours, with complete degradation after 9 days. oxoTCO **3** (25 mM) in the presence of mercaptoethanol (25 mM) showed only 8% isomerization in CD_3_OD over a 22 hours period while 92% was isomerized in phosphate buffered D_2_O (pD = 7.4) over the same period of time. Under similar conditions, oxoTCO stability to thiols in methanol is improved relative to d-TCO (92% isomerization after 14 h) and s-TCO (100% isomerization after 4 h).^30^ In D_2_O (pD = 7.4) containing 25 mM mercaptoethanol, the major diastereomer isomerized with a half-life of 2.2 hours, and the minor diastereomer isomerized with a half life of 1.6 hours. Overall, the stability of the oxoTCO diastereomers is similar to that of dTCO.^30^

**Fig. 3:**
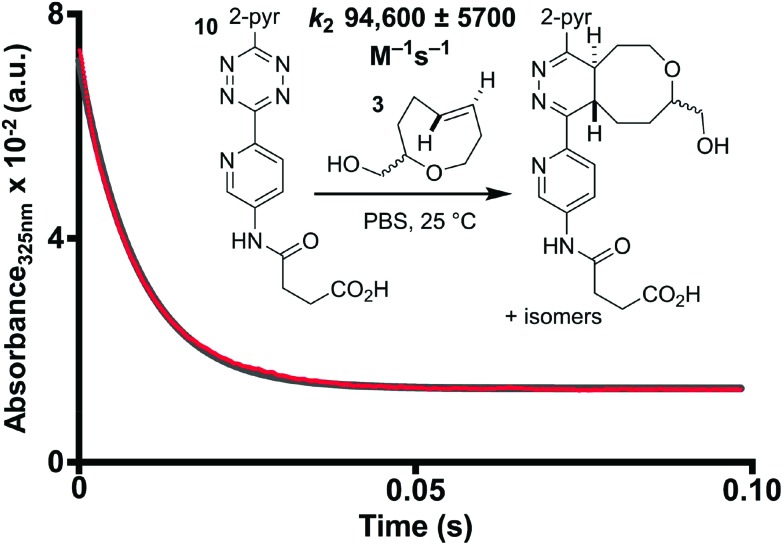
The kinetics of the cycloaddition of oxoTCO **3** with water-soluble 3,6-dipyridyl-*s*-tetrazine-mono-succinamic acid **10** in PBS buffer (pH 7.4). Second order rate constants (*k*_2_) were determined with a stopped-flow spectrophotometer under pseudo-first order conditions using *ca.* 10–30 equivalents of oxoTCO **3** (2.2 : 1 dr) by monitoring the decrease in tetrazine absorbance at 325 nm.

We next measured the rate constant for the inverse electron-demand Diels–Alder (IEDDA) cycloaddition of oxoTCO and tetrazine **10** under pseudo-first order conditions (Fig. 3). PBS was chosen as a solvent for two reasons: aqueous solvent considerably accelerates the IEDDA reaction by the hydrophobic effect and initial kinetic studies indicated tetrazine **10**, though more water soluble than 3,6-dipyridyl-*s*-tetrazine, was aggregating in unbuffered H_2_O, thus giving inconsistent first-order rates. Using a stopped-flow spectrophotometer and by following the exponential decay in tetrazine absorbance at 325 nm the second-order rate constant (*k*_2_) was determined to be 94 600 ± 5700 M^–1^ s^–1^ in PBS at 25 °C for the 2.2 : 1 diastereomeric mixture of **3**. This is faster than the reaction of a similar tetrazine with both diastereomers of 5-hydroxy-*trans*-cyclooctene (equatorial isomer 22 600 M^–1^ s^–1^; axial isomer 80 200 M^–1^ s^–1^), and is approximately ¼ as fast as a bicyclic d-TCO under comparable conditions (366 000 M^–1^ s^–1^).^30^ The diastereomerically pure equatorial isomer of **3** was obtained by SFC, and found to react with **10** with a rate constant of 44 100 ± 2600 M^–1^ s^–1^ in PBS at 25 °C. While we were unable to obtain a diasteromerically pure sample of the axial diastereomer, the rate constant can be calculated to be 310 000 M^–1^ s^–1^ based on the rates observed for the diastereomer mixture and the pure equatorial isomer. The 7-fold rate acceleration for the axial isomer is consistent with prior reports for 5-hydroxy-*trans*-cyclooctene.^11^,^30^

**Fig. 4:**
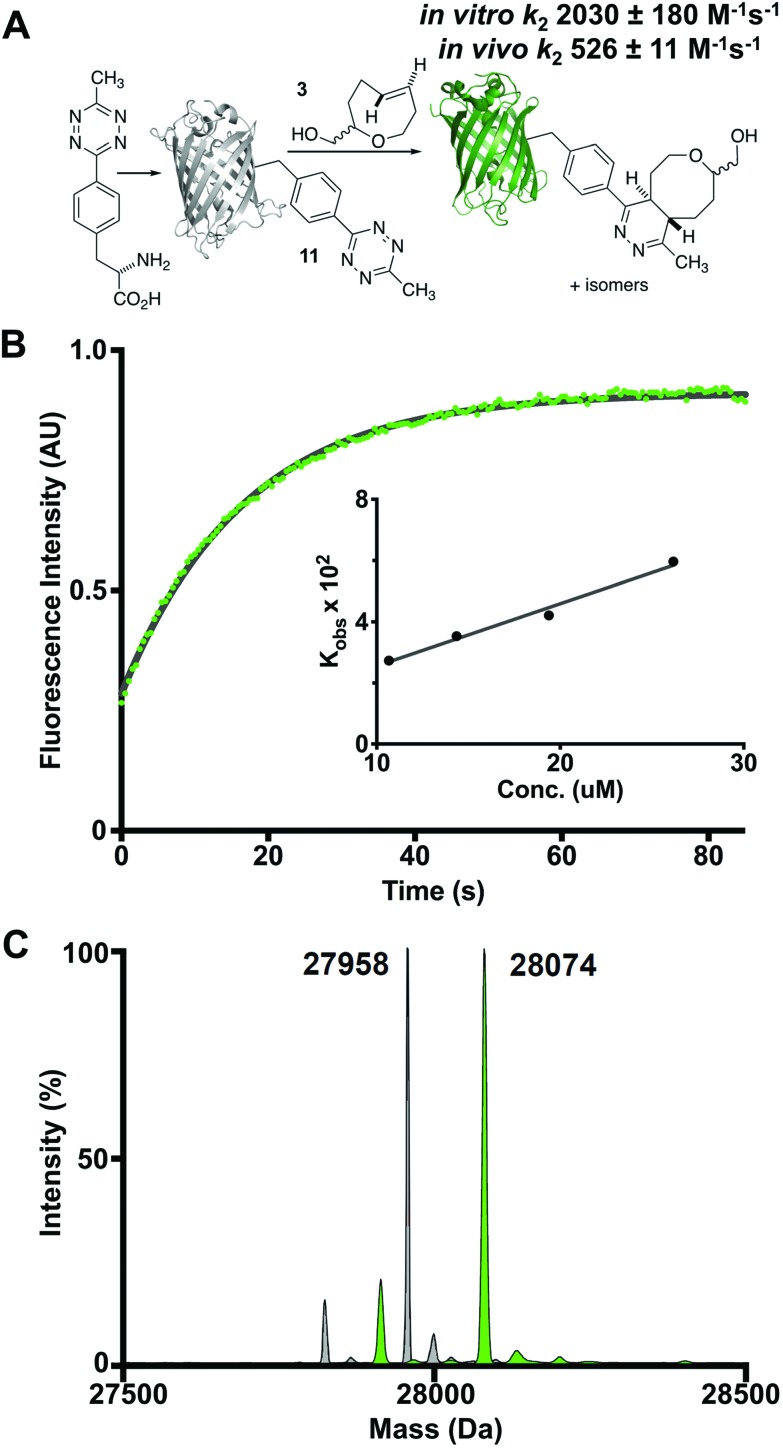
(A) The oxoTCO-tetrazine cycloaddition using a recombinant protein substrate containing a site-specifically incorporated tetrazine. Experiments were carried out both *in vitro* (PBS) and *in vivo* (*E. coli*) using a 2.2 : 1 eq/ax mixture of oxoTCO diastereomers. (B) Second order rate constants (*k*_2_) were determined under pseudo-first order conditions using *ca.* 100–260 equivalents of oxoTCO **3** by monitoring the increase in GFP fluorescence (*in vitro* study shown here). (C) Quantitative determination of the cycloadduct was confirmed by ESI-MS.

We also studied the *in vitro* cycloaddition of oxoTCO and a green fluorescent protein encoded with an unnatrual tetrazine-containing amino acid **11** (*sf*GFP-150Tet-v.2.0) *via* the procedure of Mehl and coworkers.^43^ Thus, 4-(6-methyl-*s*-tetrazin-3-yl)phenylalanine was site-specifically introduced into a C-terminally hexahistidine-tagged GFP (*sf*GFP-150TAG-His_6_) *via* orthogonal translation using the evolved aminoacyl-tRNA synthetase *Mj*RS/tRNA_CUA_ pair. Co-expression of these components in *E. coli* resulted in the amino acid-dependent synthesis of full-length recombinant GFP **11** which was purified by Ni-NTA chromatography and confirmed by ESI-MS. The tetrazine moiety of this protein quenches the fluorescence of the GFP chromophore, whereas the dihydropyridazine product of the TCO ligation does not. It is therefore possible to determine the kinetics of the reaction by monitoring the increase in GFP fluorescence (Fig. 4A). Accordingly, the second order rate constant of the reaction between oxoTCO and *sf*GFP150Tet-v.2.0 was determined to be 2030 ± 180 M^–1^ s^–1^ in phosphate buffer at room temperature (Fig. 4B). The reaction was quantitative under these conditions as determined by ESI-MS (Fig. 4C). The slower rate relative to that observed with **10** is due to the less reactive nature of the tetrazine **11** and in line with rate decreases observed with other TCOs.^30^

Finally, the small size and good hydrophilicity of oxoTCO make it an excellent candidate for labeling *in vivo*. The cycloaddition was monitored in a suspension (PBS) of *E. coli* overexpressing *sf*GFP150Tet-v.2.0 by measuring the increase in whole-cell fluorescence upon addition of **3**. At room temperature oxoTCO displays a second-order rate constant of 526 ± 11 M^–1^ s^–1^, which is approximately ¼ as fast as the *in vitro* ligation. Quantitative determination of the biorthogonal reaction was verified by ESI-MS. Cells were washed before lysis and the protein was purified *via* nickel affinity chromatography. The resulting protein mass was as expected for the cycloaddition product. This, alongside the whole-cell fluorescence experiment, provides evidence to suggest that oxoTCO crosses the bacterial cell membrane.

## Conclusions

In summary, computation was used to design a hydrophilic 5-oxo-*trans*-cyclooctene derivative with high reactivity attributed to increased angle strain. A short synthesis was developed involving Sakurai allylation, olefin metathesis and flow-enabled photoisomerization as key steps. This heterocyclic *trans*-cyclooctene displays improved hydrophilicity, with an experimental log *P* value of 0.51. Kinetic analysis revealed that oxoTCO displays faster reactivity than mono-substituted TCO dienophiles, and is less bulky than bicyclic *trans*-cyclooctenes we have described previously. Quantitative labeling of GFP containing a genetically encoded tetrazine amino acid was studied in solution and in whole bacteria cells with complete labeling within minutes at room temperature. The high reactivity and lower hydrophobicity of oxoTCO-based probes should prove useful for *in vivo* applications, and in this context is the focus of active study in our labs.

## Supplementary Material

Supplementary information
