# Correction: Computationally guided discovery of a reactive, hydrophilic *trans*-5-oxocene dienophile for bioorthogonal labeling

**DOI:** 10.1039/c7ob90144e

**Published:** 2017-08-29

**Authors:** William D. Lambert, Samuel L. Scinto, Olga Dmitrenko, Samantha J. Boyd, Ronald Magboo, Ryan A. Mehl, Jason W. Chin, Joseph M. Fox, Stephen Wallace

**Affiliations:** Brown Laboratory, Department of Chemistry & Biochemistry, University of Delaware Newark Delaware 19716 USA jmfox@udel.edu; Lotus Separations LLC Newark DE 19711 USA; Department of Biochemistry and Biophysics, Oregon State University Corvallis Oregon 97331 USA; Medical Research Council Laboratory of Molecular Biology Francis Crick Avenue Cambridge Biomedical Campus Cambridge CB2 0QH UK; Institute of Quantitative Biology, Biochemistry and Biotechnology, School of Biological Sciences, University of Edinburgh UK

## Abstract

Correction for ‘Computationally guided discovery of a reactive, hydrophilic *trans*-5-oxocene dienophile for bioorthogonal labeling’ by William D. Lambert *et al.*, *Org. Biomol. Chem.*, 2017, **15**, 6640–6644.

The authors regret that in the Introduction in one place reference 36 was incorrectly cited instead of reference 35. The correct sentence is shown below.

Very recently, Lemke, Kele and coworkers reported the genetic incorporation of dioxo-TCO **2** and demonstrated that the lower lipophilicity of this molecule resulted in improved washout times during imaging experiments. In Diels–Alder reactions with tetrazines, the reaction rate with **2** is similar to that with the parent TCO.^35,40^

The Royal Society of Chemistry apologises for these errors and any consequent inconvenience to authors and readers.

## Supplementary Material

