# Supplementary material for: Computationally guided discovery of a reactive, hydrophilic trans-5-oxocene dienophile for bioorthogonal labeling
Source: Org Biomol Chem. 2017 Jul 20;15(31):6640–4. doi: 10.1039/c7ob01707c (PMC5708333; doi:10.1039/c7ob01707c)
Supplement: Supplementary file 1 [file OB-015-C7OB01707C-s001.pdf]

Electronic Supplementary Material (ESI) for Organic & Biomolecular Chemistry.
This journal is © The Royal Society of Chemistry 2017

SUPPLEMENTARY INFORMATION

Computationally Guided Discovery of a Reactive, Hydrophilic *Trans-5*-Oxocene Dienophile for Bioorthogonal Labeling

William D. Lambert,[a] Samuel L. Scinto,[a] Olga Dmitrenko,[a] Samantha J. Boyd,[a] Ronald Magboo,[e] Ryan A. Mehl,[b] Jason W. Chin,*[c] Joseph M. Fox*[a] and Stephen Wallace*[cd]

[a] Brown Laboratory, Department of Chemistry & Biochemistry, University of Delaware, Newark, Delaware 19716, USA

[b] Department of Biochemistry and Biophysics, Oregon State University, Corvallis, Oregon 97331, United States

[c] Medical Research Council, Laboratory of Molecular Biology, Francis Crick Avenue, Cambridge Biomedical Campus, Cambridge CB2 0QH, UK

[d] Institute of Quantitative Biology, Biochemistry and Biotechnology, School of Biological Sciences, University of Edinburgh, UK

[e] Lotus Separations LLC, Newark DE 19711

Table of contents

S1. General Considerations **3**

S2. Photoisomerization apparatus **4**

S3. Silver nitrate-impregnated silica gel **4**

S4. Amber suppression experiments and protein purification **4**

S5. oxoTCO Synthesis **5-13**

S6. Separation of oxoTCO diastereomers **14-15**

S7. Tetrazine Synthesis **16**

S8. Partition Coefficient Determination **17**

S9. Stability Assays **17-19**

S10. Stopped-flow Kinetic Measurements **20-24**

S11. General Method for *in vitro* oxoTCO / GFP-Tet-v.2.0 kinetics by Fluorescence Spectroscopy **25-27**

S12. General Method for *in vivo* oxoTCO / GFP-Tet-v.2.0 kinetics by Fluorescence Spectroscopy **27-29**

S13. Computational Studies **30-48**

S14. NMR spectra **49-80**

S15. References **81**

S1. General Considerations

NMR spectra were recorded on a Bruker AV400 (^{1}H: 400 MHz, ^{13}C: 101 MHz). Chemical shifts (δ) are reported in ppm and are referenced to the residual non-deuterated solvent peak: $CDCl_3$ (7.26 ppm), d_6-DMSO (2.50 ppm), MeOD (3.31 ppm) for ^{1}H-NMR spectra; $CDCl_3$ (77.0 ppm), d_6-DMSO (39.5 ppm), MeOD (49.0 ppm) for ^{13}C-NMR spectra. ^{13}C-NMR resonances are proton decoupled. Coupling constants (J) are measured to the nearest 0.01 Hz and are presented as observed. Splitting patterns are designated as follows: s, singlet; d, doublet; t, triplet; q, quartet; quin, quintet; sext, sextet; m, multiplet. When an APT pulse sequence was used to distinguish ^{13}C NMR multiplicities, quaternary and methylene carbons appear 'up' (C or CH_2) and methane and methyl carbons appear 'down' (CH or CH_3). Mass spectrometry was carried out on a Waters GCT Premier and Thermo Q-Exactive Orbitrap. Thin layer chromatography was performed on Merck/Millipore Silica Gel 60, F_{254}. Normal phase flash chromatography was performed on Silicycle 40-63D, 60Å. Stopped-Flow kinetics experiments were performed using an Applied Photophysics SX18MV-R stopped-flow spectrophotometer with temperature control (25°C). Kinetic experiments involving fluorescence detection were performed using an Aminco-Bowman Series 2 Luminescence Spectrometer (Sim-Aminco Spectronic Instruments) at room temperature (25 °C). Anhydrous dichloromethane was dried through an alumina column solvent purification system. Anhydrous tetrahydrofuran was freshly distilled from sodium/benzophenone. Phosphate buffered D_2O (pD 7.4) was prepared by dissolving sodium dihydrogen phosphate (NaH_2PO_4, 76.7 mg) and disodium hydrogen phosphate (Na_2HPO_4, 203.5 mg) in 20 mL D_2O to make a 0.1 M solution. The pD value was confirmed on a Fisher Scientific AB15 Plus pH meter. The pH readings were converted to pD by adding 0.4 units.[1] All other solvents and chemical reagents were purchased from commercial suppliers and used without further purification. All non-aqueous reactions were carried out in flame-dried glassware under an inert atmosphere of nitrogen. Brine refers to a saturated solution of sodium chloride in water.

S2. Photoisomerization apparatus

Photoisomerizations were carried out using a modified procedure previously developed in our lab.[2] Reactions were done in a quartz flask (Southern New England Ultraviolet Company) using a Southern New England Ultraviolet Company Rayonet® reactor model RPR-100 or RPR-200, equipped with 8 low-pressure mercury lamps (2537 Å). Biotage® SNAP cartridges (Biotage part No. FSK0-1107) were used to house flash silica gel (cat # R12030B, 60 Å) and the $AgNO_3$-impregnated silica gel. The bottom of the column was interfaced to PTFE tubing (1/8" OD x 0.063" ID, flanged with a thermoelectric flanging tool), equipped with flangeless nylon fittings (1/4-28 thread, IDEX part no. P-582), using a female luer (1/4-28 thread, IDEX part no. P-628). The top of the column was interfaced using a male luer (1/4-28 thread, IDEX part no. P- 675). The pump used for recirculating solvents through the photolysis apparatus was purchased from Fluid Metering, Inc. (FMI pump model RP-D equipped with pumphead FMI R405). Adapters for interfacing the FMI pump to the PTFE tubing were purchased from IDEX (part no. U-510).

S3. Silver nitrate-impregnated silica gel

Flash silica gel (90 g, Silicycle cat # R12030B, 60 Å) was suspended in 100 mL of water in a 2 L round bottom flask. The flask was covered with aluminum foil and a silver nitrate (10 g) solution in water (10 mL) was added. The resulting mixture was thoroughly mixed. Water was evaporated under reduced pressure on the rotavap (bath temperature ~ 65 °C) using a bump trap with a coarse fritted disk. To remove the remaining traces of water, toluene (2 x 200 mL) was added and subsequently evaporated by rotary evaporation. The silver nitrate impregnated silica was then dried under vacuum overnight at room temperature.

S4. Amber suppression experiments and protein purification

Using a reported procedure, recombinant sfGFP-150Tet-v.2.0 was overproduced in *E. coli* DH10b cells using the plasmids psfGFP150TAG-His_6/PylT and pDule_tet2.0, and the full-length protein was purified by Ni-NTA chromatography.[3]

S5. oxoTCO Synthesis

tert-butyl(oxiran-2-ylmethoxy)diphenylsilane

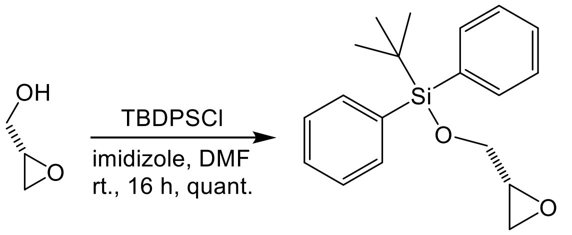

tert-Butylchlorodiphenylsilane (12.0 mL, 46.1 mmol) was added dropwise over 30 min to a stirring solution of glycidol (2.28 g, 30.8 mmol) and imidazole (4.61 g, 67.7 mmol) in DMF (20 mL) at 0 °C. The mixture was warmed to room temperature, stirred for 16 h, diluted with Et_2O (300 mL) and water (200 mL) was added slowly. The phases were separated and the aqueous phase extracted with Et_2O (3 x 200 mL). The combined organics were washed with sodium bicarbonate (aq. sat., 300 mL), brine (300 mL), dried over sodium sulfate, filtered and concentrated under reduced pressure. The crude material was purified by silica gel chromatography (0-2% EtOAc in hexane) to yield the silyl epoxide as a pale yellow oil (9.58 g, quant.). ^{1}H NMR (400 MHz, $CDCl_3$) δ 7.73-7.70 (4H, m), 7.48-7.40 (6H, m), 3.88 (1H, dd, *J*=12.0, 3.2 Hz), 3.73 (1H, dd, *J*=12.0, 4.8 Hz), 3.18-3.14 (1H, m), 2.78 (1h, dd, *J*=5.2, 4.0 Hz), 2.65 (1H, dd, *J*=5.2, 2.8 Hz), 1.08 (9H, s); ^{13}C NMR (100 MHz, $CDCl_3$) δ [135.65, 135.59][a], [133.27, 133.26][a], 129.78, 127.74, 64.28, 52.32, 44.49, 26.76, 19.26. IR (dry film) ν_{max} (cm^{-1}): 3071, 3050, 2930, 2858, 1589, 1472, 1427, 1113. HRMS (LIFDI-TOF) *m/z* : [M - t-butyl]$^+$, Calcd for $C_{15}H_{15}O_2Si^+$ 255.0836; found 255.0848

[a] Diastereotopic carbons

1-((*tert*-butyldiphenylsilyl)oxy)hex-5-en-2-ol (7)

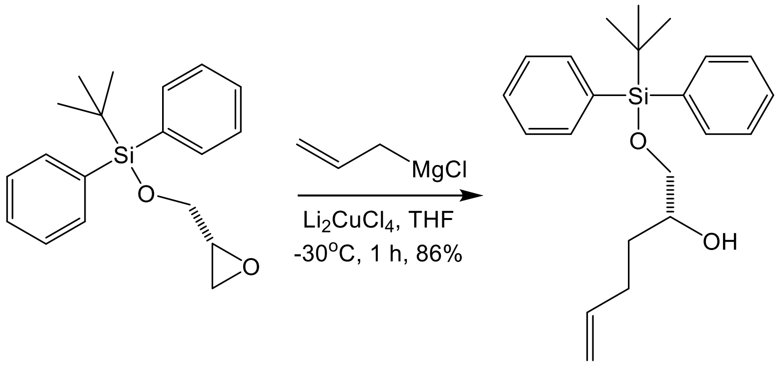

Allylmagnesium chloride (1M solution in THF, 4.54 mL, 9.09 mmol) was added dropwise over 30 min to a stirring solution of silyl epoxide (2.34 g, 7.57 mmol) and dilithium tetrachlorocuprate (0.1 M solution in THF, 7.57 mL, 0.76 mmol) in THF (11 mL) at −30 °C. The mixture was stirred at −30 °C for an additional 30 min, brought to room temperature, then diluted with Et_2O (70 mL) and ammonium chloride (aq. sat., 70 mL) which was added slowly. The phases were separated and the aqueous phase extracted with Et_2O (3 x 70 mL). The combined organics were washed with water (70 mL), brine (70 mL), dried over sodium sulfate and concentrated under reduced pressure. The crude material was purified by silica gel chromatography (0-3% EtOAc in hexane) to yield alcohol **7**. (2.29 g, 86%). ^{1}H NMR (400 MHz, $CDCl_3$) δ 7.70-7.64 (4H, m), 7.49-7.40 (6H, m), 5.82 (1H, ddt, *J*=16.9, 10.2, 6.6 Hz), 5.02 (1H, dd, *J*=17.2, 1.6), 4.98-4.96 (1H, m), 3.79-3-73 (1H, m), 3.69 (1H, dd, *J*=10.2, 3.2 Hz), 3.52 (1H, dd, *J*=10.2, 7.2 Hz), 2.52 (1H, br s), 2.24-2.06 (2H, m), 1.61-1.44 (2H, m), 1.09 (9H, s); ^{13}C NMR (100 MHz, $CDCl_3$) δ 138.32, [135.57, 135.55][a], [133.13, 133.11][a], 129.85, 127.81, 114.79, 71.35, 67.90, 31.90, 29.76, 26.85, 19.27. IR (dry film) ν_{max} (cm^{-1}): 3575, 3455, 3072, 3050, 2931, 2858, 1641, 1589, 1472, 1428, 1113. HRMS (LIFDI-TOF) *m/z* : [M - t-butyl]$^+$, Calcd for $C_{18}H_{21}O_2Si^+$ 297.1305; found 297.1292

[a] Diastereotopic carbons

5-(but-3-en-1-yl)-9,9-dimethyl-8,8-diphenyl-2,4,7-trioxa-8-siladecane

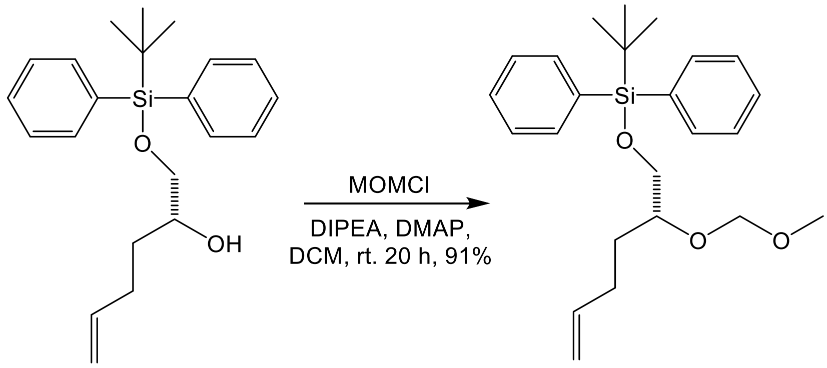

Chloromethyl methyl ether (0.61 mL, 8.0 mmol) was added dropwise over 30 min to a stirring solution of alcohol **7** (1.42 g, 4.0 mmol), DIPEA (2.1 mL, 12.0 mmol) and DMAP (10 mg, 0.08 mmol) in DCM (10 mL) at 0 °C. The mixture was warmed to room temperature and stirred for 20 h, diluted with DCM (50 mL) and water (50 mL) was added. The phases were separated and the aqueous phase extracted with DCM (3 x 50 mL). The combined organics were washed with brine (25 mL), dried over sodium sulfate and concentrated under reduced pressure. The crude material was purified by silica gel chromatography (0-1.5% EtOAc in hexane) to yield the methoxylmethyl silyl ether as a pale yellow oil (1.57 g, 98%). ^{1}H NMR (400 MHz, $CDCl_3$) δ 7.71-7.69 (4H, m), 7.48-7.39 (6H, m), 5.84 (1H, ddt, *J*=17.2, 10.4, 6.4 Hz), 5.04 (1H, dd, *J*=17.2, 1.6), 5.00-4.97 (1H, m), 4.79 (1H, d, *J*=6.8 Hz), 4.66 (1H, d, *J*=6.8 Hz), 3.74-3.61 (3H, m), 3.38 (3H, s), 2.23-2.07 (2H, m), 1.74-1.60 (2H, m), 1.08 (9H, s); ^{13}C NMR (100 MHz, MeOD) δ 138.19, [135.34, 135.31][a], [133.14, 133.13][a], 129.56, 127.44, 113.79, 95.86, 77.41, 65.83, 54.45, 30.77, 29.26, 25.93, 18.63. IR (dry film) ν_{max} (cm^{-1}): 3072, 3050, 2931, 2858, 1641, 1589, 1472, 1428, 1113, 1037. HRMS (LIFDI-TOF) *m/z* : [M - t-butyl]$^+$, Calcd for $C_{20}H_{25}O_3Si^+$ 341.1567; found 341.1597

[a] Diastereotopic carbons

((2-(but-3-en-1-yloxy)hex-5-en-1-yl)oxy)(*tert*-butyl)diphenylsilane (8)

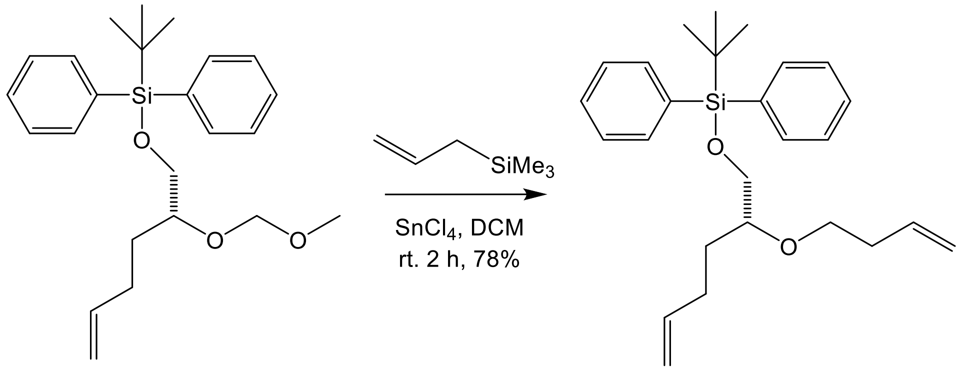

Tin(IV) chloride (1.0 M solution in DCM, 3.0 mL, 3.0 mmol) was added dropwise over 30 min to a stirring solution of methoxylmethyl silyl ether (1.44 g, 3.62 mmol) and allyltrimethyl silane (1.15 mL, 7.24 mmol) in DCM (29 mL) at 0 °C. The mixture was warmed to room temperature and stirred for 2 h, cooled again to 0 °C, diluted with DCM (30 mL) and water (50 mL) was added slowly. The phases were separated and the aqueous phase extracted with DCM (3 x 30 mL). The combined organics were washed with brine (50 mL), dried over sodium sulfate and concentrated under reduced pressure. The crude material was purified by silica gel chromatography (5-20% toluene in hexane) to yield butenyl ether **8** as a colorless oil (1.15 g, 78%).^{1}H NMR (400 MHz, $CDCl_3$) δ 7.73-7.70 (4H, m), 7.48-7.40 (6H, m), 5.83 (2H, ddt, *J*=17.0, 10.2, 6.6 Hz), 5.12-4.97 (4H, m), 3.73-3.59 (3H, m), 3.47 (1H, dt, *J*=9.2, 7.2 Hz), 3.40-3.37 (1H, m), 2.35-2.30 (2H, m), 2.20-2.10 (2H, m), 1.68-1.57 (2H, m), 1.08 (9H, s); ^{13}C NMR (100 MHz, $CDCl_3$) δ 138.74, [135.65, 135.46][a], [133.61, 133.58][a], 129.66, 127.68, 116.22, 114.57, 79.66, 69.76, 66.12, 34.72, 30.96, 29.69, 26.83, 19.22. IR (dry film) ν_{max} (cm^{-1}): 3072, 3051, 2931, 2858, 1641, 1473, 1428, 1113. HRMS (LIFDI-TOF) *m/z* : [M - t-butyl]$^+$, Calcd for $C_{22}H_{27}O_2Si^+$ 351.1775; found 351.1627

[a] Diastereotopic carbons

(*Z*)-*tert*-butyldiphenyl((3,4,7,8-tetrahydro-2*H*-oxocin-2-yl)methoxy)silane (9)

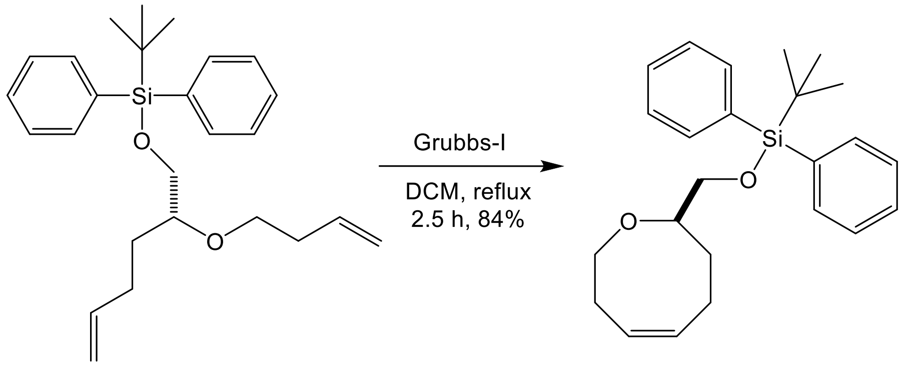

Grubbs first-generation catalyst (528 mg, 0.64 mmol) was added to a solution of butenyl ether **8** (2.62 g, 6.42 mmol) in DCM (1.28 L). The mixture was refluxed at 50 °C for 2.5 h, cooled to room temperature and concentrated under reduced pressure. The crude material was purified by silica gel chromatography (40-80% toluene in hexane) to yield *cis*-oxocine **9** as a colorless oil (2.04 g, 84%). ^{1}H NMR (400 MHz, $CDCl_3$) δ 7.73-7.68 (4H, m), 7.46-7.38 (6H, m), 5.85-5.75 (2H, m), 4.04-3.99 (1H, m), 3.64-3.61 (1H, m), 3.57-3.44 (3H, m), 2.51-2.45 (2H, m), 2.15-2.11 (1H, m), 2.07-2.02 (1H, m), 1.57-1.45 (2H, m), 1.08 (9H, s); ^{13}C NMR (100 MHz, $CDCl_3$) δ [135.65, 135.59][a], [133.89, 133.68][a], 131.50, 129.57, 129.55, 128.75, 127.63, 80.70, 71.54, 67.86, 31.71, 29.46, 26.82, 23.27, 19.24. IR (dry film) ν_{max} (cm^{-1}): 3071, 3049, 3016, 2930, 2858, 1652, 1589, 1472, 1458, 1428, 1133, 1113, 1080. HRMS (LIFDI-TOF) *m/z* : [M - t-butyl]$^+$, Calcd for $C_{20}H_{23}O_2Si^+$ 323.1462; found 323.1467

[a] Diastereotopic carbons

(*Z*)-(3,4,7,8-tetrahydro-2*H*-oxocin-2-yl)methanol

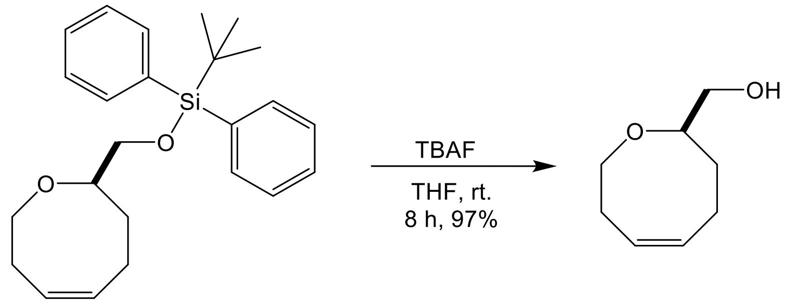

Tetrabutylammonium fluoride (1.0 M solution in THF, 7.9 mL, 7.9 mmol) was added to a stirring solution of *cis*-oxocine **9** (597 mg, 1.57 mmol) at 0 °C in THF (0.7 mL). The mixture was warmed to room temperature, stirred for 8 h, diluted with Et_2O (15 mL) and quenched slowly with ammonium chloride solution (aq. sat., 15 mL). The phases were separated and the aqueous phase extracted with Et_2O (3 x 15 mL). The combined organic extracts were washed with brine (30 mL), dried over sodium sulfate, filtered and concentrated carefully under reduced pressure (20 °C, p>200 mBar). The crude material was purified by silica gel chromatography (50% Et_2O in hexane) to yield *cis*-oxocene as a clear oil (215 mg, 97%). ^{1}H NMR (400 MHz, $CDCl_3$) δ 5.87 (2H, m), 4.09-4.03 (1H, m), 3.58-3.40 (4H, m), 2.50-2.42 (2H, m), 2.22-2.15 (1H, m), 2.12-2.03 (2H, m), 1.63-1.46 (2H, m); ^{13}C NMR (100 MHz, $CDCl_3$) δ 131.58, 128.53, 80.45, 71.99, 66.60, 31.56, 29.26, 23.16. IR (dry film) ν_{max} (cm^{-1}): 3424, 3016, 2930, 2863, 1647, 1458, 1112, 1073, 1039. HRMS (ESI) *m/z* : $[M+H]^+$, Calcd for $C_8H_{15}O_2^+$ 143.1072; found 143.1065

(*E*)-(3,4,7,8-tetrahydro-2*H*-oxocin-2-yl)methanol (oxo-TCO, 3)

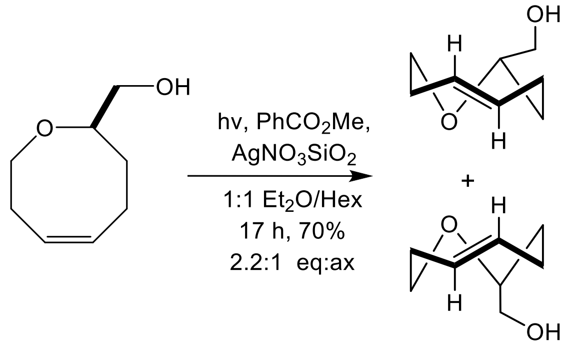

cis-oxocene was isomerized photochemically using the continuous flow apparatus. A Biotage SNAP cartridge (Biotage SNAP cartridge KP-Si 10g) was used to house the silica gel and $AgNO_3$ impregnated silica gel. A SNAP cartridge that contained a bed of unmodified silica gel (~1 in.) was topped with 5.14 g of silica gel which was impregnated with $AgNO_3$ (0.54 g, 3.18 mmol). Methyl benzoate (329 mg, 2.42 mmol) was added to a solution of *cis*-oxocene (200 mg, 1.20 mmol) in Et_2O:hexane (1:1, 100 mL) within a quartz reaction flask. The solution was equilibrated through the continuous flow system at a 100 mL/min flow rate. The solution in the quartz flask was then irradiated (254 nm) under continuous flow conditions (100 mL/min) for 17 hours. The SNAP cartridges were flushed with 100 mL of 1:1 Et_2O/hexanes and then dried with compressed air. The dried silica gel was transferred to an Erlenmeyer flask. Concentrated aqueous NH_4OH (100 mL) and DCM (100 mL) were sequentially added to the flask. The resulting biphasic mixture was stirred vigorously for 15 min, and then filtered. The filter cake was washed with additional DCM (25 mL) and ammonium hydroxide (25 mL). This was repeated three times. The combined filtrates were partitioned and the aqueous phase extracted with DCM (3 x 25 mL). The combined organics were dried over sodium sulfate, filtered and concentrated under reduced pressure (20 °C, p>200 mBar). The crude material was purified by silica gel chromatography (60-100% Et_2O) to yield oxoTCO as an inseparable mixture of diastereomers (141 mg, 70%, d.r. 2.2:1). IR (dry film) ν_{max} (cm^{-1}): 3419, 3010, 2932, 2861, 1638, 1452, 1430, 1206, 1093, 1059. HRMS (ESI) *m/z* : $[M+H]^+$, Calcd for $C_8H_{15}O_2^+$ 143.1072; found 143.1065

Peaks due to major diastereomer ^{1}H NMR (400 MHz C_6D_6) δ 5.76 (1H, ddd, J=16.0, 11.2, 3.2 Hz), 5.14 (1H, ddd, J=15.6, 11.2, 4.0 Hz), 3.83-3.79 (1H, m), 3.25 (2H, d, J=5.2 Hz), 2.88 (1H, td, J=11.6, 3.2 Hz), 2.66 (1H, dt, J=10.0, 6.0 Hz), 2.31-2.18 (2H, m), 1.94-1.85 (2H, m), 1.80-1.72 (1H, m), 1.65-1.53 (1H, m), 1.43-1.38 (1H, m); ^{13}C NMR (100 MHz, C_6D_6) δ 141.14, 127.01, 86.12, 74.33, 66.40, 38.33, 37.92, 34.34

Peaks due to minor diastereomer ^{1}H NMR (400 MHz C_6D_6) δ 5.71-5.64 (1H, m), 5.61-5.53 (1H, m), 3.85-3.79 (1H, m), 3.34-3.25 (3H, m), 3.19-3.17 (1H, m), 2.31-2.18 (1H, m), 2.16-2.02 (2H, m), 1.80-1.71 (1H, m), 1.65-1.53 (2H, m), 1.47-1.45 (1H, m); ^{13}C NMR (100 MHz, C_6D_6) δ 137.09, 134.40, 84.82, 75.86, 65.82, 39.82, 32.35, 27.36

Diasteromers of *O*-Benzoyl-(*E*)-(3,4,7,8-tetrahydro-2*H*-oxocin-2-yl)methanol

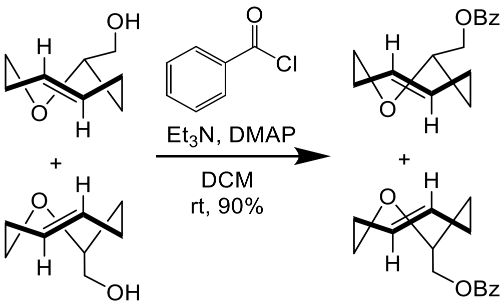

Diastereomers of oxoTCO **3** (93 mg, 0.56 mmol, 2.2:1 isomeric mixture) were added to a stirring solution of benzoyl chloride (0.080 mL, 0.69 mmol), triethylamine (0.46 mL, 3.3 mmol), and 4-dimethylaminopyridine (87.4mg, 0.72 mmol) in 4.3 mL dichloromethane. The reaction was stirred 18 hours, concentrated under reduced pressure, and then dissolved in 25 mL diethyl ether. This solution was washed with 1M HCl (2x5 mL), $NaHCO_3$ (aq. sat., 5 mL), and brine (1x5 mL). The organics were dried with $MgSO_4$, filtered, and concentrated under reduced pressure. The crude material was purified by silica gel chromatography (5-20% EtOAc in hexane) to yield the title compound, a clear oil, as a mixture of diastereomers (123 mg, 90%, d.r. 5:1). IR (dry film) ν_{max} (cm^{-1}): 3063, 3001, 2936, 2863, 1720, 1639, 1602, 1585, 1451, 1276. HRMS (LIFDI-TOF) *m/z* : [M+], Calcd for $C_{15}H_{18}O_3$ 246.1256; found 246.1256

Peaks due to major diastereomer ^{1}H NMR (400 MHz C_6D_6) δ 8.22-8.18 (2H, m), 7.13-7.04 (3H, m), 5.75 (1H, ddd, *J* = 15.3, 11.4, 3.3), 5.12 (1H, ddd, *J* = 15.5, 11.0, 3.8), 4.17 (1H, dd, *J* = 11.1, 7.8), 4.05 (1H, dd, *J* = 11.6, 4.6), 3.88 (1H, ddd, *J* = 11.8, 6.2, 1.5), 2.94-2.87 (2H, m), 2.29-2.19 (2H, m), 2.01-1.80 (2H, m), 1.65-1.55 (1H, m), 1.50-1.42 (1H, m); ^{13}C NMR (100 MHz, C_6D_6) δ 166.17, 140.89, 132.97, 131.08, 129.96, 128.64, 127.00, 82.75, 74.01, 68.19, 38.27, 37.47, 34.24,

Peaks due to minor diastereomer ^{1}H NMR (400 MHz C_6D_6) δ 8.22-8.18 (2H, m), 7.13-7.04 (3H, m), 5.70-5.62 (1H, m), 5.44 (1H, ddd, *J* = 16.3, 9.7, 5.3), 4.31 (1H, dd, *J* = 11.7, 9.0), 4.07-4.03 (1H,m), 3.77-3.66 (2H, m), 3.47 (1H, app dt, *J* = 12.0, 6.0), 2.18-2.09 (2H, m), 2.01-1.80 (2H, m), 1.74-1.66 (1H, m), 1.65-1.55 (1H, m); ^{13}C NMR (100 MHz, C_6D_6) δ 166.09, 137.48, 133.02, 132.68, 130.97, 129.94, 128.64, 79.25, 72.86, 66.69, 39.71, 34.11, 28.39

S6. Separation of oxoTCO diastereomers

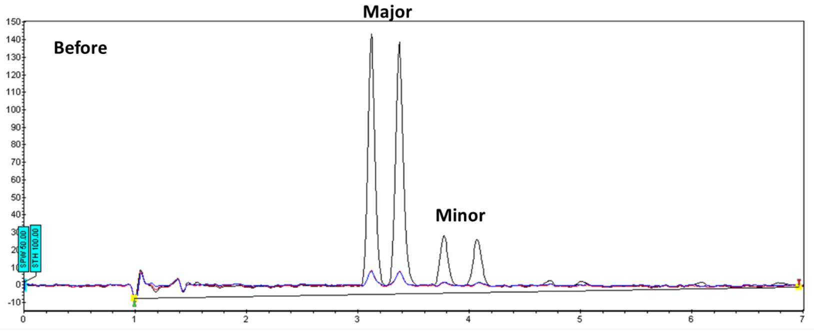

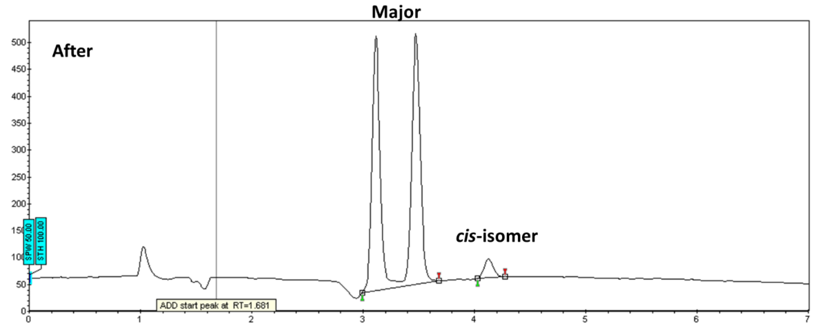

The benzoylated oxoTCO diastereomers (80 mg, 5:1 d.r.) were separated via supercritical fluid chromatography (Daicel Chiralpak AD-H column, 8% Ethanol isocratic in CO_2, 220 nm, 28 C, 100 bar at 65 ml/min. Preparative separation afforded 30 mg of the major diastereomer. Attempts to recover the minor diastereomer were unsuccessful

(*E*)-(3,4,7,8-tetrahydro-2*H*-oxocin-2-yl)methanol (3-major)

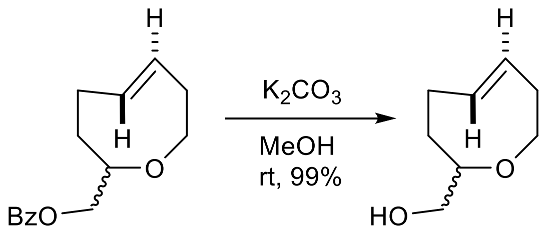

(3-major)

oxoTCO-OBz (30mg, 0.12 mmol, mixture of major isomer with 23% *cis*-isomer by ^{1}H NMR) was added to a stirring solution of K_2CO_3 (32mg, 0.23 mmol) in 7.0 mL methanol at room temperature. The reaction was stirred 5 hours and then concentrated under reduced pressure. A column was prepared with silver nitrate silica gel and hexanes (4 x ½ inches silica) and the crude mixture chromatographed (35% EtOAc in hexane) until any byproduct and *cis*-isomer has eluted. The major oxoTCO isomer was then eluted with 30 mL ammonium hydroxide and extracted with dichloromethane (5x20 mL). The solution was concentrated and the sample chromatographed through a normal-phase silica column (25% EtOAc in hexane) to yield the major diastereomer of oxoTCO as a clear oil (13 mg, 73% from crude mass, 99% yield of major diastereomer). IR (dry film) ν_{max} (cm^{-1}): 3408, 3009, 2932, 2861, 1640, 1452, 1206, 1093, 1059. ^1H NMR (400 MHz, C_6D_6) δ = 5.75 (1H, ddd, *J* = 15.3, 11.4, 3.3), 5.13 (1H, ddd, *J* = 15.4, 11.0, 3.8), 3.80 (1H, ddd, *J* = 11.7, 6.1, 1.6), 3.25 (2H, d, *J* = 6.0), 2.87 (1H, td, *J* = 11.7, 3.3), 2.65 (1H, dt, *J* = 9.8, 5.9), 2.3-2.21 (2H, m), 1.96-1.84 (2H, m), 1.71 (1H, s), 1.64-1.54 (1H, m), 1.40 (1H, dd, *J* = 14.2, 5.1). ^{13}C NMR (100 MHz, C_6D_6) δ = 141.16, 127.03, 86.15, 74.35, 66.41, 38.34, 37.92, 34.35. HRMS (ESI) *m/z* : $[M+H]^+$, Calcd for $C_8H_{15}O_2^+$ 143.1072; found 143.1065

S7. Tetrazine Synthesis

4-oxo-4-((6-(6-(pyridin-2-yl)-1,2,4,5-tetrazin-3-yl)pyridin-3-yl)amino)butanoic acid (10)

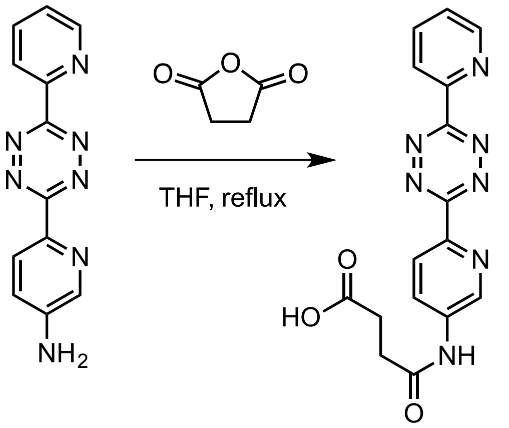

Prepared previously by our lab: see reference [4]

*sf*GFP150Tet-v.2.0 (11)

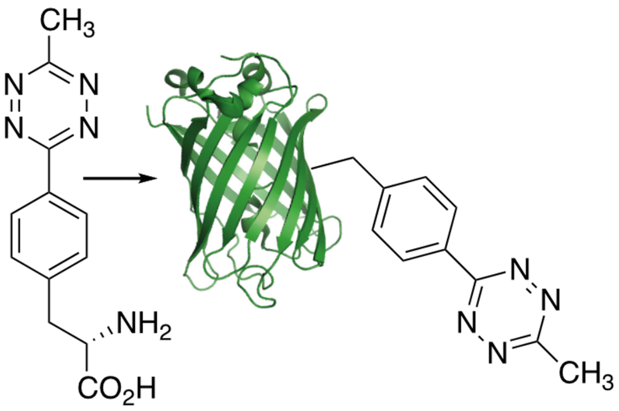

Prepared by previously developed method: see reference [3]

S8. Partition Coefficient (LogP) Determination

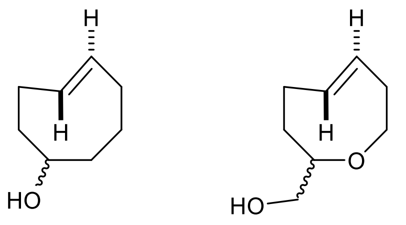

LogP = 1.1 LogP = 0.51

(E)-(3,4,7,8-tetrahydro-2H-oxocin-2-yl)methanol **(3)** (5.1 mg, 0.036 mmol, 2.2:1 diastereomer mixture) was dissolved into a 2 mL solution of 1:1 water and 1-octanol. The solution was stirred for 20 hours and the phases were separated. A 1.91 mM solution of 3,6-di-(2-pyridyl)-1,2,4,5-tetrazine was then titrated with each oxoTCO solution over multiple trials. The organic phase concentration was found to be 14.7 mM and the aqueous phase was found to be 4.5 mM. Log(P) was then calculated as 0.51.

As a comparison, the partition of equatorial 5-hydroxy-transcyclooctene was measured using the same procedure. The log(P) was calculated as 1.1

S9. Stability Assays

Stability of oxoTCO 3 in methanol-d_4

A solution of oxoTCO **3** (3.5 mg, 25 μmol, 2.5:1 d.r.) in methanol-d_4 (750 μL) was monitored by quantitative proton NMR on a 600 MHz instrument to observe the decomposition of the *trans*-isomer. t-Butyl methyl ether (3.0 μL, 25 μmol) was used as an internal standard. After 14 days there was no measureable decomposition of oxoTCO **3** (**Fig. S9-a**). A waterfall plot of the periodic ^{1}H NMR spectra is provided.

Stability of oxoTCO 3 in phosphate buffered D_2O (pD 7.4)

A solution of oxoTCO **3** (3.4 mg, 24 μmol, 2.5:1 d.r.) in phosphate buffered D_2O (pD 7.4, 750 μL) was monitored by quantitative proton NMR on a 600 MHz instrument to observe the decomposition of the *trans*-isomer. t-Butyl methyl ether (2.8 μL, 24 μmol) was used as an internal standard. After 7 days >90% of

the major isomer remained, and after 14 days 82% of the major diastereomer remained. The minor diastereomer had completely decomposed after 9 days with a 1st order exponential decay ($t_{1/2}$ = 1.5 days)(**Fig. S9-a**). A waterfall plot of the periodic ^{1}H NMR spectra is provided.

Fig. S9-a) Long-term stability of oxoTCO **3** in methanol-d_4 and phosphate buffered D_2O (pD 7.4)

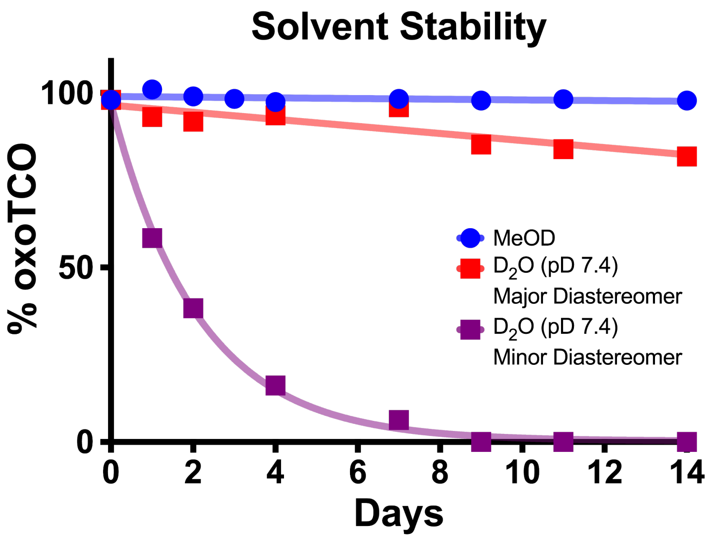

Stability of oxoTCO 3 in methanol-d_4 and mercaptoethanol

A solution of oxoTCO **3** (2.6 mg, 18 μmol, 25 mM, 2.5:1 d.r.) and mercaptoethanol (1.3 μL, 18 μmol, 25 mM) in methanol-d_4 (750 μL) was monitored by quantitative proton NMR every 2 hours for 22 hours total on a 600 MHz instrument to observe the decomposition of the *trans*-isomer. t-Butyl methyl ether (2.1 μL, 18 μmol) was used as an internal standard. After 22 hours 92% of the total oxoTCO (sum of both diastereomers) remained (**Fig. S9-b**). A waterfall plot of the periodic ^{1}H NMR spectra is provided.

Stability of oxoTCO 3 in phosphate buffered D_2O (pD 7.4) and mercaptoethanol

A solution of oxoTCO **3** (2.6 mg, 18 μmol, 25 mM 2.5:1 d.r.) and mercaptoethanol (1.3 μL, 18 μmol, 25 mM) in phosphate buffered D_2O (pD 7.4) was monitored by quantitative proton NMR every 2 hours for 22 hours total on a 600 MHz

instrument to observe the decomposition of the *trans*-isomer. t-Butyl methyl ether (2.1 μL, 18 μmol) was used as an internal standard. After 22 hours 7% of the major diastereomer remained with a 1st order exponential decay ($t_{1/2}$ = 2.2 hours) The minor diastereomer was completely isomerized after 10 hours ($t_{1/2}$ = 1.6 hours) (**Fig. S9-b**). A waterfall plot of the periodic ^{1}H NMR spectra is provided.

Fig. S9-b) Stability of oxoTCO **3** in methanol-d_4 and phosphate buffered D_2O (pD 7.4) in the presence of mercaptoethanol

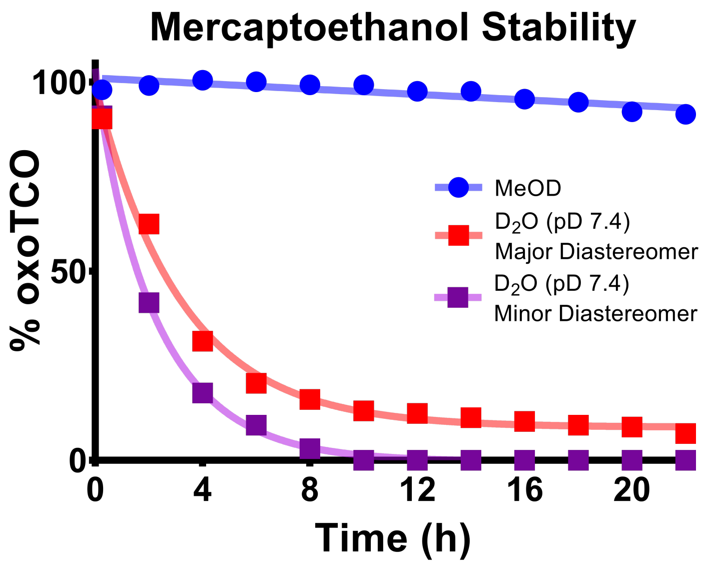

S10. Stopped-flow kinetic measurements

Kinetics of the diastereomer mixture

The reaction between oxoTCO **3** and a tetrazine succinate **10** was measured under pseudo-first order conditions using a SX 18MV-R stopped-flow spectrophotometer (Applied Photophysics Ltd.). A 0.1 mM solution of tetrazine succinate was fleshly prepared in PBS and oxoTCO solutions (2.2:1 diastereomer mixture; 0.98, 1.46, 1.95, 2.63 mM) were also prepared in PBS. The oxoTCO and tetrazine solutions were injected as equal volumes via syringe into the stopped-flow instrument at 25 °C, resulting in final concentrations of 0.0500 mM tetrazine and 0.490, 0.730, 0.970, 1.32 mM oxoTCO respectively. The reaction was monitored by UV-vis absorbance spectroscopy at 325 nm. Data points were collected every 0.1 ms for 0.1 s. Four runs were conducted for each oxoTCO solution and the data was compiled using Prism software. The observed rate of each solution, k_{obs} (**Fig. S10-a**), was determined by nonlinear regression analysis resulting in rate constants of 37.0, 59.0, 81.2, 115 s^{-1} respectively and were then plotted against final concentration to determine the bimolecular rate constant k_2 (**Fig. S10-b**) of 94,600 (+/- 5700) $M^{-1}s^{-1}$ from the slope of the plot.

Fig. S10-a) Observed rate of four oxoTCO **3** solutions reacted with tetrazine **10**

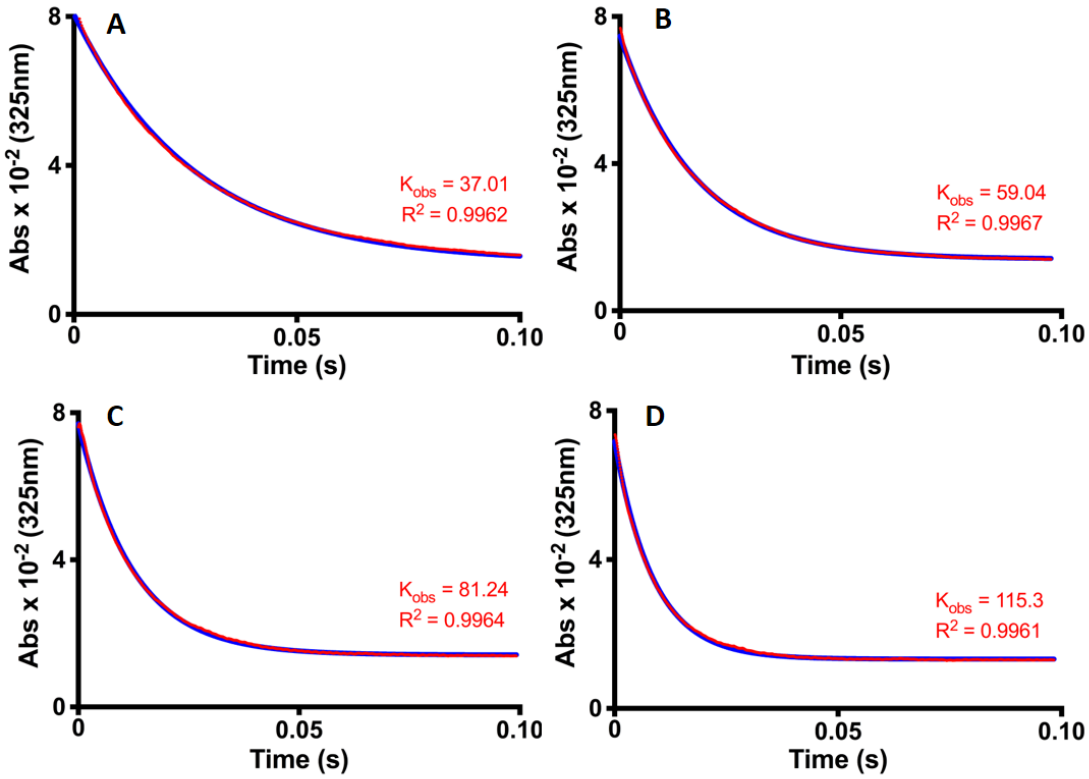

Figure S10-a: Stopped-flow kinetics of oxoTCO **3** (2.2:1 diastereomer mixture) and tetrazine succinate **10**. After injection, the final tetrazine concentration was 0.05mM and the final oxoTCO concentrations are as follows; **A**=0.49mM, **B**=0.73mM, **C**=0.97mM, **D**=1.32mM. Four trials for each concentration were obtained and averaged (shown in red) and the best fit curve was calculated on Prism software (shown in blue).

Fig. S10-b) Bimolecular rate determination of four oxoTCO **3** solutions reacted with tetrazine **10** by plotting k_{obs} vs final oxoTCO concentration

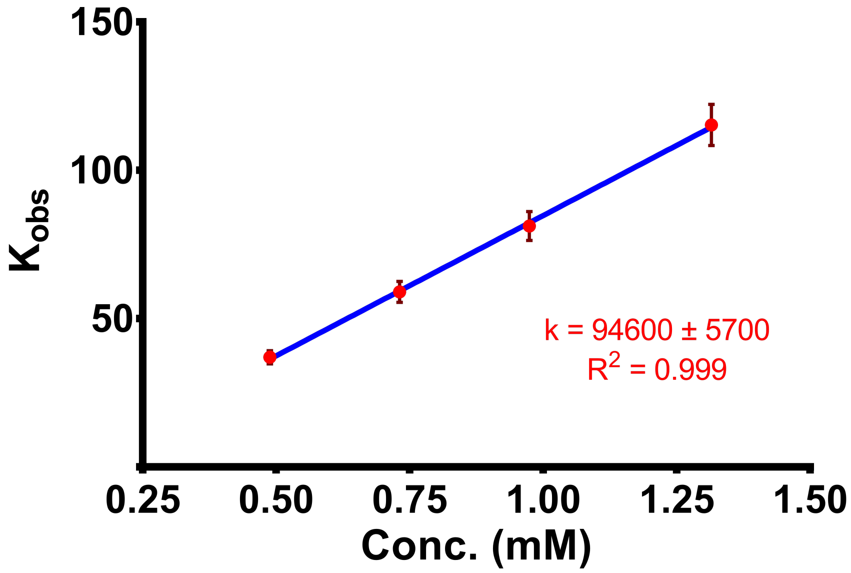

Kinetics of the major diastereomer

The reaction between the major diastereomer of oxoTCO **3-major** and a tetrazine succinate **10** was measured under pseudo-first order conditions using a SX 18MV-R stopped-flow spectrophotometer (Applied Photophysics Ltd.). A 0.1 mM solution of tetrazine succinate was fleshly prepared in PBS and oxoTCO solutions (0.96, 1.92, 2.79, 3.76 mM) were also prepared in PBS from a 3.76 ± 0.05 mM stock solution. The oxoTCO and tetrazine solutions were injected as equal volumes via syringe into the stopped-flow instrument at 25 °C, resulting in final concentrations of 0.0500 mM tetrazine and 0.480, 0.960, 1.34, 1.88 mM oxoTCO respectively. The reaction was monitored by UV-vis absorbance spectroscopy at 325 nm. Data points were collected every 0.1 ms for 0.2 s. Five runs were conducted for each oxoTCO solution and the data was compiled using Prism software. The observed rate of each solution, k_{obs} (**Fig. S10-c**), was determined by nonlinear regression analysis resulting in rate constants of 20.6, 41.8, 61.1, 82.3 s^{-1} respectively and were then plotted against final concentration to determine the bimolecular rate constant k_2 (**Fig. S10-d**) of 44,100 (+/- 2600) $M^{-1}s^{-1}$ from the slope of the plot.

Fig. S10-c) Observed rate of four solutions of the major diastereomer of oxoTCO **3** reacted with tetrazine **10**

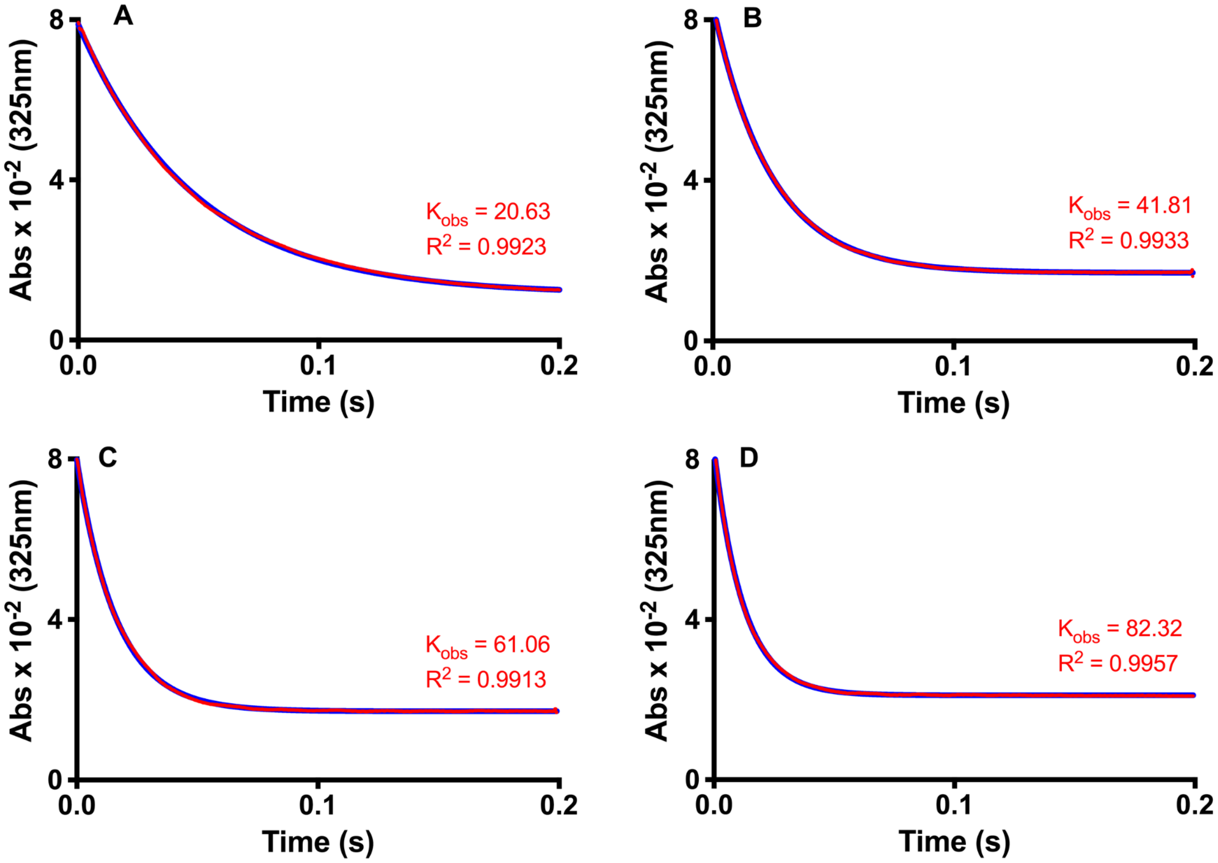

Figure S10-c: Stopped-flow kinetics of the major diastereomer of oxoTCO **3** and tetrazine succinate **10**. After injection, the final tetrazine concentration was 0.05mM and the final oxoTCO concentrations are as follows; **A**=0.480mM, **B**=0.960mM, **C**=1.34mM, **D**=1.88mM. Five trials for each concentration were obtained and averaged (shown in red) and the best fit curve was calculated on Prism software (shown in blue).

Fig. S10-d) Bimolecular rate determination of four solutions of the major diastereomer of oxoTCO **3** reacted with tetrazine **10** by plotting k_{obs} vs final oxoTCO concentration

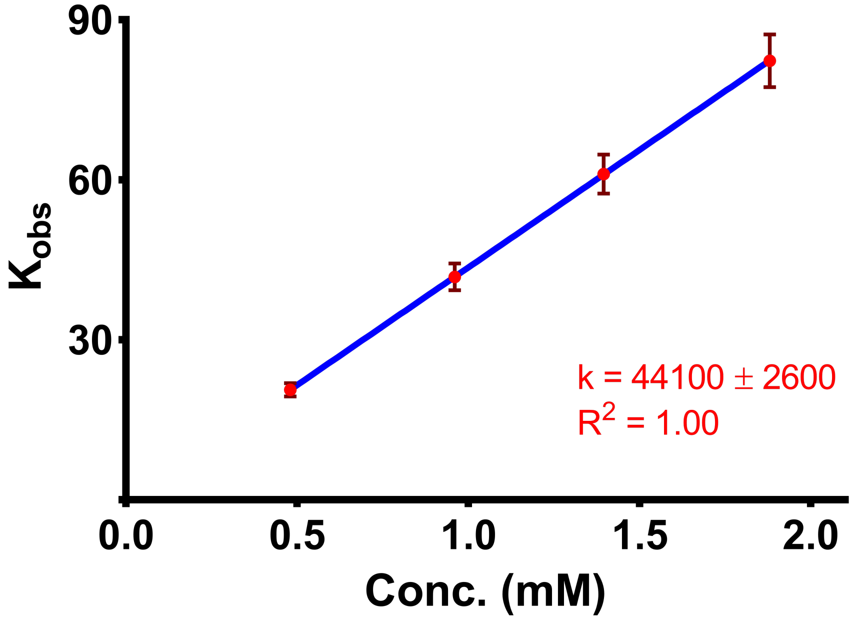

S11. General Method for *in vitro* oxoTCO / GFP-Tet-v.2.0 kinetics by Fluorescence Spectroscopy

The reaction between oxoTCO **3** and a GFP-tetrazine 2.0 **11** was measured under pseudo-first order conditions using an Aminco-Bowman Series 2 Luminescence Spectrometer (Sim-Aminco Spectronic Instruments). A 30 µL sample of 8.4 µM GFP-tetrazine 2.0 in PBS buffer was diluted in 2.5 mL additional PBS buffer in a 4 mL glass cuvette w/stir bar for a final concentration of 0.100 µM. oxoTCO solutions (0.550, 0.750, 1.00, 1.35 mM) were prepared in methanol from a stock 7.86 ± 0.2 mM solution. Fluorescence data collection began on the GFP-tetrazine solution at 25 °C (excitation wavelength of 488 nm w/2 nm slit, emission wavelength of 506 nm w/5 nm slit) after which 50 µL of the oxoTCO solution was injected rapidly via syringe resulting in final oxoTCO concentrations of 10.7, 14.4, 19.4, 26.2 µM respectively. Data collection was taken in 0.5 sec increments for about 2 minutes for each trial. The observed rate of each solution, k_{obs} (**Fig. S11-a**), was determined by nonlinear regression analysis using Prism software resulting in rates constants of 0.0273, 0.0353, 0.0421 and 0.0597 s^{-1} respectively and were then plotted against final concentration to determine the bimolecular rate constant k_2 (**Fig. S11-b**) of 2030 (+/- 180) $M^{-1}s^{-1}$ from the slope of the plot.

Fig. S11-a) *in vitro* observed rate of four oxoTCO **3** solutions conjugated with GFP-tetrazine 2.0 **11**

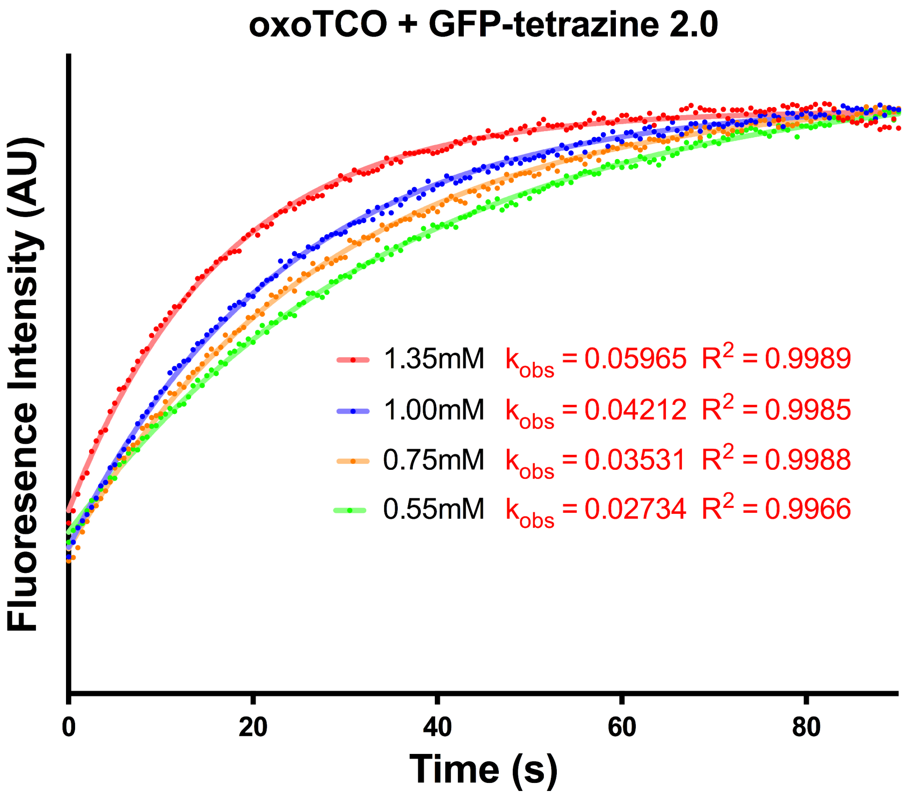

Figure S11-a: Fluorescence kinetics of oxoTCO **3** (2.2:1 diastereomer mixture) and GFP-tetrazine 2.0 **11.** After injection, the final tetrazine concentration was 0.1μM and the final oxoTCO concentrations are as follows; 10.66, 14.35, 19.38, 26.16 μM. Raw data points and the fit curve for a pseudo first order rate equation calculated on Prism software are shown.

Fig. S11-b) *in vitro* bimolecular rate determination of four oxoTCO **3** solutions reacted with GFP-tetrazine 2.0 **11** by plotting k_{obs} vs final oxoTCO concentration

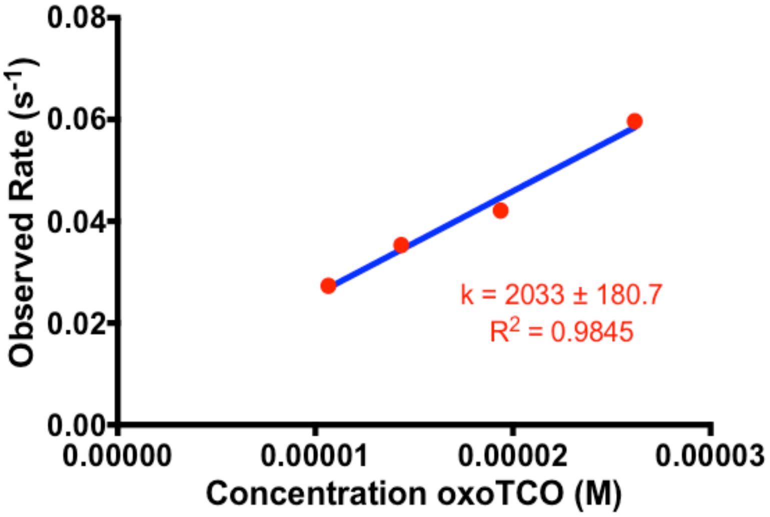

S12. General Method for *in vivo* oxoTCO / GFP-Tet-v.2.0 kinetics by Fluorescence Spectroscopy

A 50 mL culture of *E. coli* overexpressing GFP-TetV2.0 **11** was resuspended in PBS buffer and washed three times. Three pseudo-first order kinetic trials containing 500 μL of cell solution in 2 mL of PBS were initiated by adding 50 μL of stock oxoTCO **3** solutions (2.0, 1.0, and 0.75 mM). Fluorescence increase was monitored, indicating conjugation of oxoTCO to GFP-TetV2.0 (Excitation 488 nm, Emission 506 nm, and 1s increments). For each trial, fluorescence was measured until a constant emission intensity was observed. Unimolecular rate constants were calculated for each concentration using Prism software (0.0244, 0.0137, 0.0113 s^{-1} respectively) (**Fig. S12-a**) and a bimolecular constant was calculated by plotting each unimolecular rate constants against oxoTCO concentration (526 (+/- 11) $M^{-1}s^{-1}$) (**Fig. S12-b**).

In Vivo Mass Spec Analysis

A 50 mL culture of *E. coli* overexpressing GFP-TetV2.0 **11** was resuspended in 15 mL PBS buffer and washed three times. Excess oxoTCO **3** was added to a 500 μL cell suspension at a final concentration of 400 μM and allowed to react at room

temperature for one hour. The 500 μL cell suspension was spun down and washed 3 times with PBS. The cell pellet was resuspended in lysis buffer (50 mM Tris, NaCl 150 mM, sodium deoxycholate 0.5%, Triton X-100 1%, pH 7.5) and rocked for 30 minutes at room temperature. The cell solution was spun down, the supernatant was purified by nickel affinity chromatography. The eluted fractions were applied to a 10 mL Amicon centrifugal filter (10,000 MWCO, EMD Millipore) and underwent three rounds of buffer exchange with 50 mM KPi, pH 7.5, 0.1mM EDTA. The cell lysis solution was analyzed by LC-ESI-MS (**Fig. S12-c**).

Fig. S12-a) *in vivo* observed rate of three oxoTCO **3** solutions reacted with GFP-tetrazine 2.0 **11**

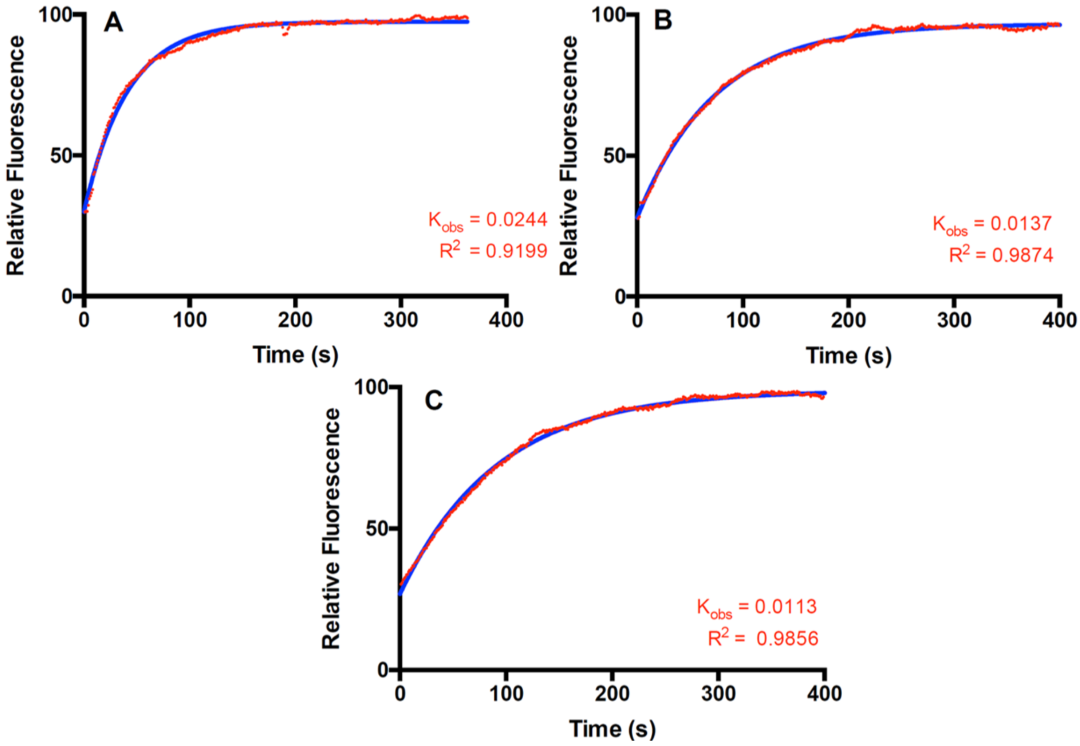

Figure S12-a: Fluorescence kinetics of oxoTCO **3** and GFP-tetrazine 2.0 **11**. After injection, the final oxoTCO concentrations are as follows; **A**=0.182mM, **B**=0.091mM, **C**=0.068mM. Raw data points (red) and the fit curve (blue) for a pseudo first order rate equation calculated on Prism software are shown.

Fig. S12-b) *in vivo* bimolecular rate determination of three oxoTCO **3** solutions reacted with GFP-tetrazine 2.0 **11** by plotting k_{obs} vs final oxoTCO concentration

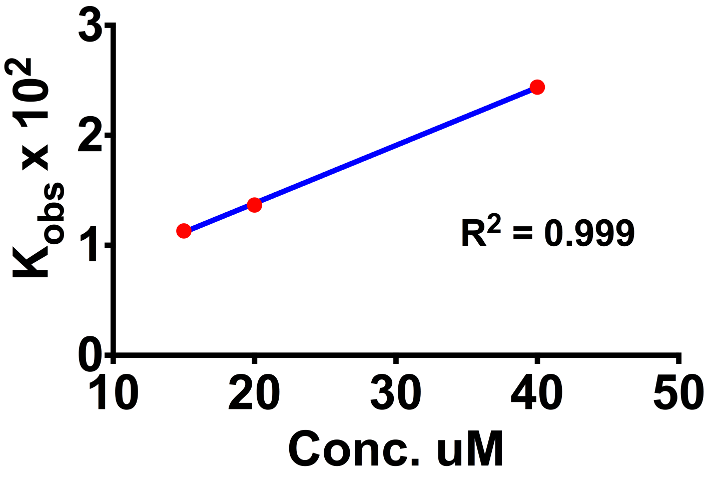

Fig. S12-c) ESI mass spectrum of *in vivo* oxoTCO and GFPtet-2.0 cycloaddition product

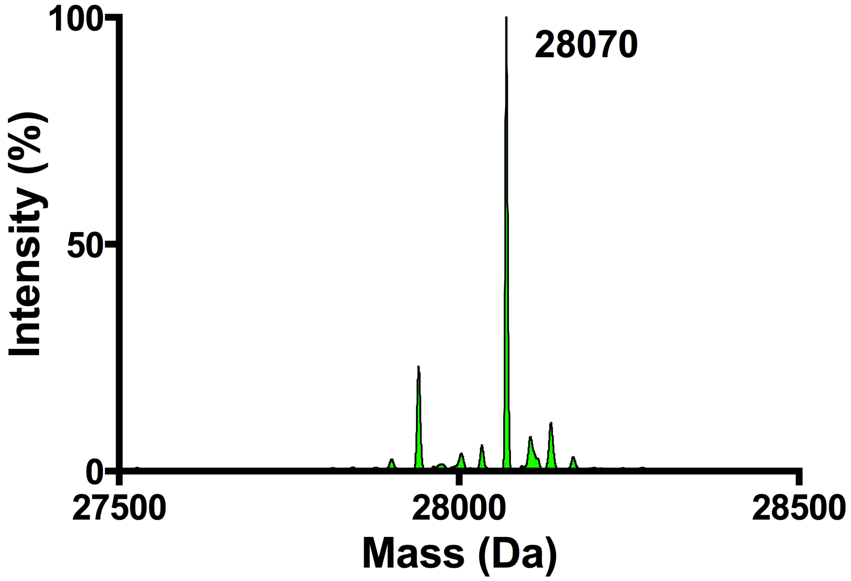

Figure S12-c: Deconvoluted mass spectrum with a major peak at 28070 ± 1 Da (28070 expected) indicating successful *in vivo* conjugation of GFP-TetV2.0 with oxoTCO. Analysis was performed on the Xevo GS-2 QTof (Waters Corp.) system and MaxEnt was used for deconvolution of spectra.

S13. Computational Studies

Pre-reaction complex of trans-cyclooctene with 3,6-diphenyl-s-tetrazine
M06L/6-311+G(d,p)

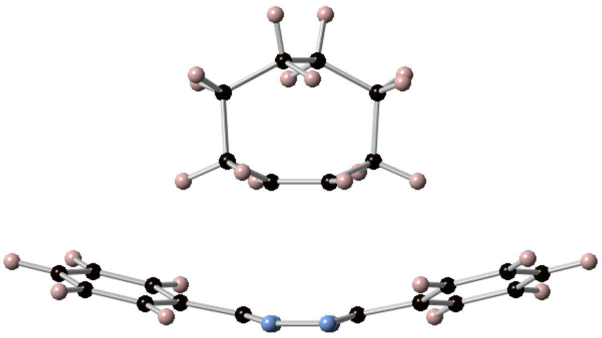

Electronic Energy-1071.83471322 au
Sum of electronic and zero-point Energies -1071.417219 au
Sum of electronic and thermal Energies -1071.394458 au
Sum of electronic and thermal Enthalpies -1071.393514 au
Sum of electronic and thermal Free Energies -1071.470561 au

Atomic Coordinates in Angstroms.

Atomic Number	X	Y	Z
6	-0.362610	3.919674	0.687390
6	0.362405	3.919724	-0.687105
6	-1.622304	1.656411	0.928082
6	1.622247	1.656563	-0.927932
6	-0.660249	1.200787	-0.108721
6	0.660217	1.200812	0.108836
6	-1.715074	3.194865	0.772151
6	1.714918	3.195011	-0.771898
1	-0.533840	4.960322	0.983711
1	0.533567	4.960397	-0.983384
1	-1.243578	1.413660	1.929022
1	1.243539	1.413857	-1.928890
1	-1.011492	1.220711	-1.144037
1	1.011458	1.220676	1.144153
1	0.308656	3.515095	1.455859
1	-0.308834	3.515133	-1.455591
1	-2.620560	1.216194	0.836712
1	2.620530	1.216400	-0.836588
1	-2.294937	3.596895	1.611212
1	2.294760	3.597131	-1.610930
1	-2.298775	3.423037	-0.129421
1	2.298597	3.423165	0.129694
7	0.668630	-1.982176	-1.170423
7	-0.668557	-1.982342	1.170202
6	1.277901	-1.783032	0.015315
6	-1.277837	-1.783080	-0.015512
7	0.640008	-1.987502	1.188272
7	-0.639935	-1.987386	-1.188493
6	2.712205	-1.497509	0.030578
6	3.340626	-1.130039	1.226341
6	3.456605	-1.534542	-1.154912

6	4.688684	-0.802207	1.232529
1	2.756662	-1.100195	2.139841
6	4.806018	-1.213071	-1.140018
1	2.959518	-1.814032	-2.077406
6	5.425128	-0.843028	0.051114
1	5.168590	-0.511683	2.161703
1	5.378212	-1.247400	-2.061405
1	6.479311	-0.584833	0.058644
6	-2.712151	-1.497612	-0.030738
6	-3.340592	-1.130031	-1.226457
6	-3.456545	-1.534811	1.154750
6	-4.688663	-0.802251	-1.232601
1	-2.756634	-1.100060	-2.139956
6	-4.805970	-1.213391	1.139900
1	-2.959443	-1.814388	2.077210
6	-5.425100	-0.843236	-0.051188
1	-5.168585	-0.511640	-2.161740
1	-5.378159	-1.247848	2.061285
1	-6.479293	-0.585080	-0.058683

CAM-B3LYP/tzvp
Electronic Energy-1071.43961170 au
Sum of electronic and zero-point Energies -1071.018962 au
Sum of electronic and thermal Energies -1070.995576 au
Sum of electronic and thermal Enthalpies -1070.994631 au
Sum of electronic and thermal Free Energies -1071.078672 au

Atomic Coordinates in Angstroms.

Atomic Number	*X*	*Y*	*Z*
6	0.526034	4.459614	-0.573861
6	-0.525999	4.459620	0.573829
6	1.787126	2.172388	-0.538495
6	-1.787101	2.172399	0.538476
6	0.612124	1.716684	0.254019
6	-0.612101	1.716684	-0.254035
6	1.851877	3.707633	-0.343317
6	-1.851845	3.707643	0.343290
1	0.791123	5.499556	-0.777516
1	-0.791084	5.499565	0.777476
1	1.642091	1.947137	-1.597884
1	-1.642067	1.947152	1.597866
1	0.725589	1.735048	1.336056
1	-0.725567	1.735042	-1.336072
1	0.063658	4.094778	-1.494614
1	-0.063624	4.094789	1.494584
1	2.730843	1.720284	-0.227436
1	-2.730820	1.720298	0.227420
1	2.609824	4.111982	-1.019583
1	-2.609790	4.111999	1.019554
1	2.206199	3.920744	0.669705
1	-2.206166	3.920749	-0.669733
7	-0.659351	-1.726103	1.168934
7	0.659335	-1.726129	-1.168922
6	-1.283577	-1.680629	-0.010855
6	1.283561	-1.680637	0.010866
7	-0.638941	-1.724396	-1.179907
7	0.638925	-1.724378	1.179919
6	-2.755755	-1.613183	-0.023499
6	-3.445761	-1.565021	-1.232382
6	-3.467741	-1.589973	1.173265

6	*-4.827237*	*-1.495171*	*-1.240948*
1	*-2.888946*	*-1.584297*	*-2.158330*
6	*-4.849140*	*-1.520481*	*1.158106*
1	*-2.927791*	*-1.627465*	*2.108548*
6	*-5.532340*	*-1.472889*	*-0.047379*
1	*-5.356379*	*-1.458855*	*-2.184397*
1	*-5.395357*	*-1.503927*	*2.092328*
1	*-6.613494*	*-1.418800*	*-0.056707*
6	*2.755739*	*-1.613198*	*0.023509*
6	*3.445747*	*-1.565027*	*1.232391*
6	*3.467725*	*-1.590002*	*-1.173256*
6	*4.827223*	*-1.495183*	*1.240955*
1	*2.888932*	*-1.584293*	*2.158340*
6	*4.849124*	*-1.520515*	*-1.158099*
1	*2.927774*	*-1.627501*	*-2.108538*
6	*5.532325*	*-1.472915*	*0.047386*
1	*5.356365*	*-1.458861*	*2.184404*
1	*5.395341*	*-1.503972*	*-2.092320*
1	*6.613480*	*-1.418830*	*0.056713*

TS for the reaction of trans-cyclooctene with 3,6-diphenyl-s-tetrazine
M06L/6-311+G(d,p)

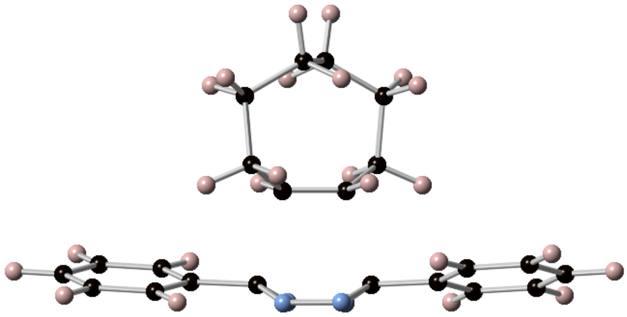

Electronic Energy-1071.81350424 au
Sum of electronic and zero-point Energies -1071.395018 au
Sum of electronic and thermal Energies -1071.373860 au
Sum of electronic and thermal Enthalpies -1071.372916 au
Sum of electronic and thermal Free Energies -1071.444919 au
Imaginary Frequency 513.5374i cm-1

Atomic Coordinates in Angstroms.

Atomic Number	X	Y	Z
6	2.983914	-0.600357	-0.489833
6	2.983882	0.600462	0.489979
6	0.709077	-1.835888	-0.240033
6	0.709013	1.835903	0.240014
6	0.152327	-0.589794	0.370744
6	0.152307	0.589806	-0.370791
6	2.234493	-1.859072	-0.040335
6	2.234451	1.859157	0.040471
1	4.023552	-0.889366	-0.674798
1	4.023503	0.889513	0.674968
1	0.474766	-1.850858	-1.311428
1	0.474613	1.850877	1.311389
1	0.319527	-0.480050	1.443788
1	0.319546	0.480060	-1.443835
1	2.605954	-0.280728	-1.470436

1	2.605886	0.280827	1.470566
1	0.279444	-2.741911	0.198584
1	0.279369	2.741897	-0.198650
1	2.627836	-2.729313	-0.577283
1	2.627698	2.729367	0.577541
1	2.450576	-2.043499	1.020175
1	2.450661	2.043673	-1.019995
7	-2.315259	1.207313	0.583782
7	-2.315251	-1.207451	-0.583862
6	-1.915484	1.021933	-0.728825
6	-1.915439	-1.022048	0.728720
7	-2.306113	-0.167996	-1.330339
7	-2.306086	0.167856	1.330259
6	-1.855206	2.208895	-1.602095
6	-1.556649	2.054010	-2.958721
6	-2.064616	3.492839	-1.089709
6	-1.473219	3.162724	-3.789689
1	-1.406988	1.054057	-3.354229
6	-1.983492	4.599505	-1.926097
1	-2.303172	3.605919	-0.037479
6	-1.687423	4.439008	-3.275993
1	-1.245247	3.031928	-4.842873
1	-2.155058	5.592235	-1.521838
1	-1.625123	5.305421	-3.926686
6	-1.855076	-2.208972	1.602001
6	-1.556350	-2.054018	2.958580
6	-2.064580	-3.492931	1.089699
6	-1.472797	-3.162692	3.789586
1	-1.406655	-1.054035	3.354010
6	-1.983336	-4.599558	1.926129
1	-2.303289	-3.606060	0.037510
6	-1.687065	-4.439001	3.275973
1	-1.244679	-3.031846	4.842733
1	-2.154965	-5.592306	1.521940
1	-1.624677	-5.305385	3.926697

CAM-B3LYP/tzvp
Electronic Energy-1071.41376620 au
Sum of electronic and zero-point Energies -1070.990424 au
Sum of electronic and thermal Energies -1070.969471 au
Sum of electronic and thermal Enthalpies -1070.968527 au
Sum of electronic and thermal Free Energies -1071.040307 au
Imaginary Frequency 447.6844i cm-1

Atomic Coordinates in Angstroms.

Atomic Number	*X*	*Y*	*Z*
6	-0.257934	3.793987	0.732322
6	0.251509	3.794408	-0.731896
6	-1.403512	1.492768	1.203188
6	1.400516	1.494879	-1.202656
6	-0.692231	0.924673	0.013495
6	0.690006	0.925818	-0.013016
6	-1.546889	3.015571	1.029646
6	1.541630	3.017925	-1.029209
1	-0.445419	4.830991	1.017336
1	0.437446	4.831706	-1.016855
1	-0.833219	1.277487	2.109522
1	0.830605	1.278693	-2.109011
1	-1.191900	1.080117	-0.937325
1	1.189478	1.081818	0.937835

1	*0.537612*	*3.453534*	*1.401434*
1	*-0.543512*	*3.452791*	*-1.401035*
1	*-2.397108*	*1.063108*	*1.329758*
1	*2.394743*	*1.066618*	*-1.329071*
1	*-1.992848*	*3.409016*	*1.946465*
1	*1.987000*	*3.411996*	*-1.946046*
1	*-2.271411*	*3.214595*	*0.234286*
1	*2.265843*	*3.218084*	*-0.233853*
7	*0.670606*	*-1.529015*	*-1.154359*
7	*-0.669097*	*-1.529908*	*1.154984*
6	*1.250720*	*-1.167384*	*0.036027*
6	*-1.249679*	*-1.168873*	*-0.035387*
7	*0.598093*	*-1.524584*	*1.193380*
7	*-0.596586*	*-1.525300*	*-1.192763*
6	*2.729846*	*-1.140123*	*0.096273*
6	*3.370197*	*-0.908133*	*1.309786*
6	*3.493250*	*-1.336636*	*-1.050221*
6	*4.751771*	*-0.870870*	*1.374255*
1	*2.776628*	*-0.778135*	*2.204425*
6	*4.875901*	*-1.301764*	*-0.980605*
1	*2.993317*	*-1.532057*	*-1.988412*
6	*5.509082*	*-1.066530*	*0.229262*
1	*5.240173*	*-0.693559*	*2.323869*
1	*5.461124*	*-1.462976*	*-1.876856*
1	*6.590011*	*-1.040033*	*0.281353*
6	*-2.728861*	*-1.143844*	*-0.095799*
6	*-3.369369*	*-0.912422*	*-1.309331*
6	*-3.492140*	*-1.341927*	*1.050505*
6	*-4.750989*	*-0.877257*	*-1.374020*
1	*-2.775859*	*-0.781215*	*-2.203836*
6	*-4.874831*	*-1.309158*	*0.980671*
1	*-2.992058*	*-1.536895*	*1.988709*
6	*-5.508180*	*-1.074480*	*-0.229218*
1	*-5.239508*	*-0.700373*	*-2.323654*
1	*-5.459950*	*-1.471582*	*1.876772*
1	*-6.589140*	*-1.049628*	*-0.281475*

Product of the reaction of trans-cyclooctene with 3,6-diphenyl-s-tetrazine
M06L/6-311+G(d,p)

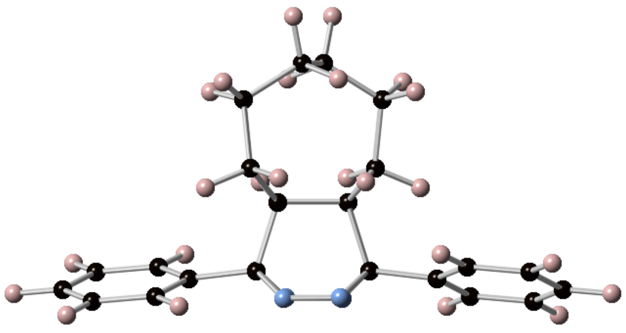

Electronic Energy-1071.85389431 au
Sum of electronic and zero-point Energies -1071.431939 au
Sum of electronic and thermal Energies -1071.411322 au
Sum of electronic and thermal Enthalpies -1071.410377 au
Sum of electronic and thermal Free Energies -1071.480812 au

Atomic Coordinates in Angstroms.

Atomic Number	X	Y	Z
6	0.212501	3.633694	-0.311927
6	-0.269423	3.485299	1.145961
6	1.367779	1.410864	-1.026075
6	-1.344232	1.128555	1.420794
6	0.789941	0.553243	0.100991
6	-0.740633	0.521349	0.153218
6	1.494587	2.891797	-0.690953
6	-1.523757	2.640811	1.372766
1	-0.478792	4.486224	1.536420
1	0.750872	1.280199	-1.924494
1	-0.720033	0.851270	2.280045
1	1.186728	0.904801	1.062221
1	-1.152551	1.035131	-0.725035
1	0.549642	3.107858	1.773455
1	2.363348	1.039694	-1.289402
1	-2.325267	0.679483	1.605625
1	1.941853	3.384393	-1.561496
1	-1.984987	2.943125	2.319546
1	2.226960	3.012106	0.119426
1	-2.262509	2.887391	0.597539
7	-0.570322	-1.692300	1.108465
7	0.693588	-1.443196	-1.257972
6	-1.187129	-0.981135	-0.019983
6	1.289021	-0.938299	-0.012944
7	-0.546921	-1.451761	-1.271116
7	0.669757	-1.660013	1.123886
6	-2.672988	-1.178201	-0.090905
6	-3.347140	-0.815570	-1.257733
6	-3.399261	-1.680877	0.988416
6	-4.727070	-0.951802	-1.344910
1	-2.780191	-0.444088	-2.106247
6	-4.780777	-1.817204	0.898761
1	-2.872850	-1.970846	1.891372
6	-5.448135	-1.452068	-0.265043
1	-5.240246	-0.671636	-2.259508
1	-5.336915	-2.212107	1.743040
1	-6.526028	-1.559389	-0.332566
6	2.781071	-1.093436	0.024133
6	3.446153	-0.936493	1.240891
6	3.520736	-1.355626	-1.128803
6	4.830279	-1.038726	1.305052
1	2.869505	-0.753404	2.142746
6	4.906450	-1.458378	-1.062218
1	3.001671	-1.486427	-2.072196
6	5.564641	-1.298694	0.152046
1	5.336496	-0.920261	2.257964
1	5.473127	-1.665555	-1.964433
1	6.645839	-1.379337	0.201440
1	0.385738	4.697200	-0.504175
1	-0.595057	3.354196	-1.002356

Pre-reaction complex of trans-4-oxyene with 3,6-diphenyl-s-tetrazine

M06L/6-311+G(d,p)

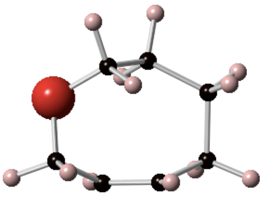

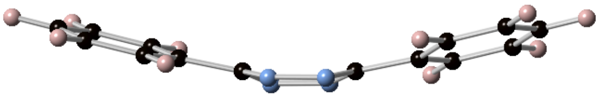

Electronic Energy-1107.73126775 au
Sum of electronic and zero-point Energies -1107.337886 au
Sum of electronic and thermal Energies -1107.315414 au
Sum of electronic and thermal Enthalpies -1107.314470 au
Sum of electronic and thermal Free Energies -1107.391356 au

Atomic Coordinates in Angstroms.

Atomic Number	X	Y	Z
6	0.458591	3.788998	-0.949021
6	1.740523	1.520318	-1.006129
6	-1.632990	1.685466	0.515059
6	0.680108	1.115602	-0.050242
6	-0.612665	1.105107	-0.395070
6	1.811430	3.063485	-0.879608
1	0.664527	4.834659	-1.203809
1	1.459362	1.244024	-2.029456
1	-1.364985	1.518980	1.570753
1	0.930247	1.191220	1.011851
1	-0.877267	1.074653	-1.453655
1	-0.138894	3.403729	-1.782475
1	2.724533	1.087716	-0.800234
1	-2.655371	1.338057	0.351712
1	2.475548	3.452501	-1.660127
1	2.298871	3.311952	0.072584
7	-0.732475	-1.879584	1.290067
7	0.630773	-2.219628	-1.010287
6	-1.323328	-1.825369	0.079661
6	1.232238	-1.881822	0.147998
7	-0.676914	-2.199029	-1.046015
7	0.575958	-1.915387	1.327606
6	-2.747855	-1.504441	0.000754
6	-3.353226	-1.318704	-1.247977
6	-3.503671	-1.325996	1.166368
6	-4.689913	-0.956791	-1.326954
1	-2.759721	-1.455321	-2.145385
6	-4.841489	-0.969121	1.079438
1	-3.024698	-1.466315	2.129245
6	-5.437453	-0.781439	-0.165045
1	-5.151691	-0.807050	-2.297537
1	-5.422646	-0.832739	1.985632
1	-6.482162	-0.494327	-0.229031
6	2.672500	-1.627512	0.143218
6	3.309391	-1.166645	1.301859
6	3.416087	-1.790736	-1.032151
6	4.665635	-0.874129	1.281589
1	2.726108	-1.040421	2.207622

6	4.772648	-1.500833	-1.043751
1	2.913544	-2.143440	-1.926058
6	5.400883	-1.040162	0.110564
1	5.152831	-0.514003	2.182108
1	5.343825	-1.632496	-1.957035
1	6.461266	-0.809365	0.097408
6	-0.432568	3.784811	0.322228
1	0.136560	3.422282	1.191470
1	-0.731558	4.811179	0.553225
8	-1.677411	3.095048	0.225448

CAM-B3LYP/tzvp
Electronic Energy-1107.34865491 au
Sum of electronic and zero-point Energies -1106.951973 au
Sum of electronic and thermal Energies -1106.928864 au
Sum of electronic and thermal Enthalpies -1106.927920 au
Sum of electronic and thermal Free Energies -1107.011640 au

Atomic Coordinates in Angstroms.

Atomic Number	*X*	*Y*	*Z*
6	0.473219	4.488310	-0.571344
6	1.795751	2.228960	-0.539854
6	-1.807941	2.226461	0.284080
6	0.577914	1.737903	0.157026
6	-0.606926	1.724344	-0.436600
6	1.798634	3.757041	-0.279558
1	0.718439	5.549722	-0.659558
1	1.728371	2.038573	-1.613084
1	-1.747992	2.010493	1.357020
1	0.614242	1.747501	1.244481
1	-0.659878	1.758413	-1.521078
1	0.082835	4.196755	-1.548129
1	2.725505	1.789249	-0.175017
1	-2.757198	1.857564	-0.099144
1	2.590717	4.212827	-0.879201
1	2.074312	3.932727	0.764849
7	-0.646340	-1.625674	1.202848
7	0.671571	-1.786357	-1.129814
6	-1.270879	-1.654190	0.022824
6	1.296881	-1.669249	0.044817
7	-0.626501	-1.778840	-1.141078
7	0.652299	-1.632552	1.213884
6	-2.741928	-1.570985	0.005171
6	-3.429400	-1.560777	-1.206210
6	-3.455278	-1.492469	1.198994
6	-4.809724	-1.473108	-1.220049
1	-2.871788	-1.622757	-2.129792
6	-4.835563	-1.405404	1.178317
1	-2.917760	-1.501270	2.136420
6	-5.516039	-1.395186	-0.029569
1	-5.336943	-1.464970	-2.165163
1	-5.382997	-1.345184	2.109967
1	-6.596227	-1.325928	-0.043174
6	2.769423	-1.607944	0.052726
6	3.461341	-1.503806	1.257112
6	3.479832	-1.646745	-1.144638
6	4.843141	-1.440163	1.260655
1	2.905919	-1.476005	2.183698
6	4.861561	-1.582737	-1.134399
1	2.938573	-1.728572	-2.076356

6	*5.546614*	*-1.479458*	*0.066531*
1	*5.373813*	*-1.361030*	*2.200605*
1	*5.406596*	*-1.614774*	*-2.068885*
1	*6.628017*	*-1.430432*	*0.071938*
6	*-0.684229*	*4.379318*	*0.459493*
1	*-0.308442*	*4.007430*	*1.418970*
1	*-1.075857*	*5.380246*	*0.639136*
8	*-1.839365*	*3.644216*	*0.062693*

TS for the reaction of trans-4-oxyene with 3,6-diphenyl-s-tetrazine
M06L/6-311+G(d,p)

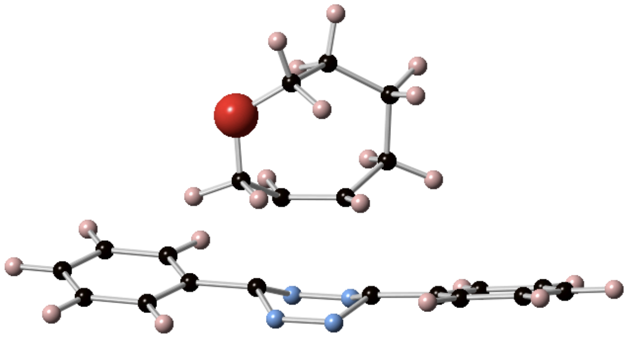

Electronic Energy-1107.70988265 au
Sum of electronic and zero-point Energies -1107.316738 au
Sum of electronic and thermal Energies -1107.295527 au
Sum of electronic and thermal Enthalpies -1107.294583 au
Sum of electronic and thermal Free Energies -1107.367078 au
Imaginary Frequency 492.1688i cm-1

Atomic Coordinates in Angstroms.

Atomic Number	X	Y	Z
6	-0.305342	3.499309	1.104830
6	1.410843	1.500567	-0.901028
6	-1.468878	1.184185	1.353345
6	0.687345	0.737208	0.174167
6	-0.703256	0.744851	0.152459
6	-1.608161	2.717490	1.299043
1	-0.489586	4.530134	1.425138
1	0.877035	1.402991	-1.860013
1	-0.932534	0.888164	2.263052
1	1.151127	0.823390	1.158100
1	-1.175269	0.955131	-0.808807
1	0.474843	3.127013	1.778814
1	2.442126	1.171748	-1.041337
1	-2.467820	0.738868	1.400337
1	-2.097251	3.046625	2.222633
1	-2.303048	2.977241	0.489328
7	-0.590217	-1.873314	1.028337
7	0.726243	-1.543636	-1.286846
6	-1.210364	-1.402320	-0.111083
6	1.300949	-1.242722	-0.060270
7	-0.553485	-1.602128	-1.311591
7	0.687592	-1.808228	1.056802
6	-2.680144	-1.375708	-0.141790
6	-3.345127	-1.024346	-1.320646
6	-3.426104	-1.659880	1.007006

6	-4.731546	-0.964378	-1.350917
1	-2.762474	-0.813263	-2.211637
6	-4.813506	-1.600581	0.970426
1	-2.903502	-1.936277	1.916688
6	-5.469892	-1.252259	-0.206067
1	-5.239553	-0.695037	-2.271450
1	-5.385235	-1.829226	1.864346
1	-6.553954	-1.207143	-0.231810
6	2.775750	-1.153827	-0.006724
6	3.411592	-0.979048	1.224777
6	3.544502	-1.221408	-1.172213
6	4.794324	-0.876584	1.291013
1	2.810943	-0.939690	2.128147
6	4.928281	-1.121704	-1.100625
1	3.045263	-1.362864	-2.124849
6	5.556684	-0.948553	0.128576
1	5.280123	-0.743497	2.252405
1	5.518834	-1.181060	-2.009377
1	6.637824	-0.869615	0.180798
6	0.277721	3.551237	-0.326194
1	0.512803	4.587978	-0.582291
1	-0.463098	3.207785	-1.064948
8	1.510489	2.864126	-0.512330

CAM-B3LYP/tzvp
Electronic Energy-1107.32321387 au
Sum of electronic and zero-point Energies -1106.924126 au
Sum of electronic and thermal Energies -1106.903400 au
Sum of electronic and thermal Enthalpies -1106.902456 au
Sum of electronic and thermal Free Energies -1106.973920 au
Imaginary Frequency 445.2463i cm-1

Atomic Coordinates in Angstroms.

Atomic Number	X	Y	Z
6	-0.354830	3.588771	1.080208
6	1.421792	1.555667	-0.862070
6	-1.476661	1.239352	1.352820
6	0.680652	0.793291	0.200439
6	-0.697700	0.777202	0.162067
6	-1.639476	2.767982	1.257854
1	-0.598918	4.618461	1.351230
1	0.910891	1.465035	-1.827356
1	-0.944981	0.977080	2.269844
1	1.142662	0.855608	1.180027
1	-1.159268	0.992795	-0.796707
1	0.410306	3.272472	1.792802
1	2.451309	1.227608	-0.975977
1	-2.466817	0.786625	1.400669
1	-2.147466	3.105576	2.164428
1	-2.320605	2.996359	0.432080
7	-0.584462	-1.807243	1.043362
7	0.718348	-1.489146	-1.266551
6	-1.198818	-1.349921	-0.093206
6	1.305882	-1.252128	-0.048573
7	-0.549704	-1.531781	-1.288848
7	0.682640	-1.765128	1.067132
6	-2.677375	-1.362511	-0.128842
6	-3.346913	-1.037291	-1.304765
6	-3.412540	-1.687757	1.007057
6	-4.730030	-1.036688	-1.342494
1	-2.774695	-0.805721	-2.192688

6	*-4.796462*	*-1.688976*	*0.963599*
1	*-2.889363*	*-1.953140*	*1.914899*
6	*-5.459105*	*-1.361599*	*-0.208658*
1	*-5.241594*	*-0.786538*	*-2.263027*
1	*-5.359578*	*-1.950626*	*1.850215*
1	*-6.541116*	*-1.363720*	*-0.240398*
6	*2.785522*	*-1.190395*	*-0.003957*
6	*3.437603*	*-1.079667*	*1.219841*
6	*3.534858*	*-1.235842*	*-1.175424*
6	*4.818654*	*-1.012584*	*1.270360*
1	*2.855566*	*-1.065146*	*2.131088*
6	*4.916663*	*-1.171578*	*-1.119800*
1	*3.025948*	*-1.335027*	*-2.123837*
6	*5.562180*	*-1.057322*	*0.101140*
1	*5.317136*	*-0.928156*	*2.227377*
1	*5.491705*	*-1.213136*	*-2.035843*
1	*6.642627*	*-1.005774*	*0.141968*
6	*0.290234*	*3.632715*	*-0.323384*
1	*0.564596*	*4.663359*	*-0.546485*
1	*-0.425543*	*3.319746*	*-1.092359*
8	*1.510135*	*2.916582*	*-0.465533*

Product of the reaction of trans-4-oxyene with 3,6-diphenyl-s-tetrazine

M06L/6-311+G(d,p)

Electronic Energy-1107.75254292 au
Sum of electronic and zero-point Energies -1107.355261 au
Sum of electronic and thermal Energies -1107.334790 au
Sum of electronic and thermal Enthalpies -1107.333846 au
Sum of electronic and thermal Free Energies -1107.404696 au

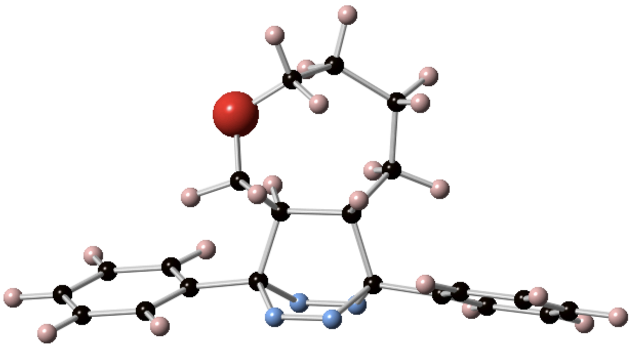

Atomic Coordinates in Angstroms.

Atomic Number	X	Y	Z
6	0.265063	3.654522	-0.257596
6	-0.352410	3.389765	1.124561
6	1.409632	1.431965	-1.006304
6	-1.380042	1.220484	1.289182
6	0.796698	0.551156	0.082593
6	-0.732410	0.526392	0.087521
6	1.544740	2.897279	-0.609546
1	-0.660080	4.341122	1.568326
1	0.814257	1.337460	-1.923517
1	-0.800043	1.006486	2.202602
1	1.164613	0.887231	1.061522
1	-1.123111	1.006022	-0.818178

1	0.385900	2.946783	1.812069
1	2.408354	1.059872	-1.255073
1	-2.392523	0.838932	1.442489
1	2.040839	3.419707	-1.434772
1	2.241742	2.967673	0.237851
7	-0.563666	-1.650770	1.136163
7	0.686979	-1.473198	-1.249689
6	-1.187150	-0.974849	-0.010371
6	1.292247	-0.941452	-0.020132
7	-0.552753	-1.484392	-1.253293
7	0.677438	-1.631047	1.137726
6	-2.673486	-1.176553	-0.066652
6	-3.354565	-0.851592	-1.240254
6	-3.391042	-1.652153	1.030614
6	-4.733817	-0.998339	-1.316534
1	-2.794734	-0.499353	-2.101373
6	-4.771994	-1.798780	0.951502
1	-2.859547	-1.912884	1.939470
6	-5.446584	-1.471750	-0.219405
1	-5.252981	-0.746069	-2.235713
1	-5.321897	-2.171602	1.809666
1	-6.524175	-1.586400	-0.278272
6	2.783860	-1.100137	0.009070
6	3.458024	-0.932964	1.219436
6	3.514334	-1.371766	-1.147571
6	4.842620	-1.035520	1.273617
1	2.888484	-0.742592	2.124243
6	4.900362	-1.474828	-1.090691
1	2.988157	-1.509812	-2.086001
6	5.567867	-1.305570	0.117221
1	5.356230	-0.909600	2.221563
1	5.459997	-1.689947	-1.995424
1	6.649322	-1.386709	0.158981
1	0.492812	4.724319	-0.313399
1	-0.510343	3.486305	-1.014709
8	-1.536835	2.606389	1.110055

Pre-reaction complex of trans-5-oxyene with 3,6-diphenyl-s-tetrazine
M06L/6-311+G(d,p)

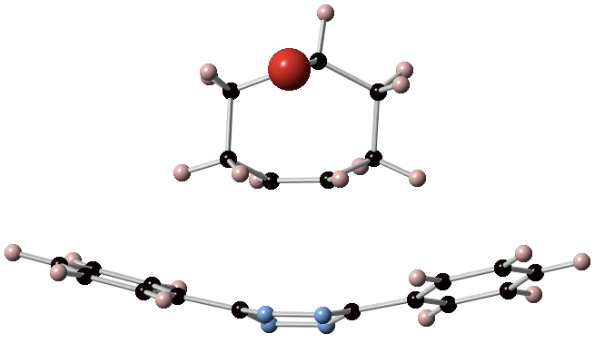

Electronic Energy-1107.73492746 au
Sum of electronic and zero-point Energies -1107.341760 au
Sum of electronic and thermal Energies -1107.319328 au
Sum of electronic and thermal Enthalpies -1107.318384 au
Sum of electronic and thermal Free Energies -1107.395001 au

Atomic Coordinates in Angstroms.

Atomic Number	X	Y	Z
6	0.372616	3.987400	-0.514951
6	1.677425	1.786004	-0.890240
6	-1.643144	1.683608	0.745536
6	0.649750	1.263515	0.044364
6	-0.653860	1.222063	-0.261963
6	1.735893	3.308068	-0.600384
6	-1.598138	3.235938	0.683440
1	0.503132	5.072549	-0.623489
1	1.374372	1.616786	-1.930934
1	-1.353962	1.358041	1.751280
1	0.917793	1.283751	1.103308
1	-0.952798	1.254857	-1.312693
1	-0.272131	3.658951	-1.342084
1	2.673011	1.349985	-0.757680
1	-2.672442	1.353340	0.566006
1	2.333349	3.811803	-1.369674
1	-2.139407	3.659645	1.535555
1	2.255201	3.475520	0.349928
1	-2.104458	3.577952	-0.230684
7	-0.714125	-1.832331	1.216692
7	0.676388	-2.024337	-1.083037
6	-1.295898	-1.731750	0.004709
6	1.257345	-1.712578	0.093269
7	-0.630894	-2.039351	-1.129741
7	0.594119	-1.828023	1.264183
6	-2.727996	-1.446030	-0.073479
6	-3.321217	-1.194414	-1.316248
6	-3.504776	-1.366657	1.088964
6	-4.666491	-0.864358	-1.392686
1	-2.711131	-1.256515	-2.211058
6	-4.851214	-1.042181	1.004676
1	-3.035716	-1.557861	2.047898
6	-5.435147	-0.787652	-0.233686
1	-5.119134	-0.665194	-2.358777
1	-5.449094	-0.984662	1.908624
1	-6.487313	-0.527974	-0.295048
6	2.688609	-1.412353	0.112338
6	3.305962	-0.997959	1.298732
6	3.442499	-1.488541	-1.065236
6	4.653161	-0.665892	1.303695
1	2.714412	-0.938445	2.206043
6	4.790236	-1.159975	-1.051747
1	2.955066	-1.805959	-1.980476
6	5.399092	-0.746369	0.130424
1	5.124822	-0.342061	2.225996
1	5.369817	-1.224857	-1.967051
1	6.452576	-0.485461	0.137367
8	-0.283233	3.758210	0.737807

CAM-B3LYP/tzvp

Electronic Energy-1107.35267638 au

Sum of electronic and zero-point Energies -1106.956197 au

Sum of electronic and thermal Energies -1106.933116 au

Sum of electronic and thermal Enthalpies -1106.932172 au

Sum of electronic and thermal Free Energies -1107.015957 au

Atomic Coordinates in Angstroms.

Atomic Number	X	Y	Z
6	0.568400	4.447893	-0.566082
6	1.861295	2.195263	-0.518540
6	-1.725842	2.229088	0.390716
6	0.648581	1.742239	0.215005
6	-0.550761	1.693345	-0.350045
6	1.895967	3.730885	-0.308750
6	-1.649299	3.768707	0.201884
1	0.769156	5.517935	-0.672793
1	1.774417	1.970785	-1.584330
1	-1.655197	1.998495	1.455009
1	0.698065	1.826912	1.297737
1	-0.625301	1.657306	-1.434939
1	0.128771	4.103712	-1.506863
1	2.791334	1.753328	-0.156223
1	-2.692648	1.875186	0.026568
1	2.654330	4.174161	-0.960732
1	-2.356501	4.259065	0.872644
1	2.198679	3.950580	0.717494
1	-1.934639	4.021424	-0.825295
7	-0.713363	-1.662659	1.196946
7	0.597652	-1.754670	-1.143122
6	-1.340456	-1.640312	0.017945
6	1.225966	-1.676251	0.032597
7	-0.700661	-1.736018	-1.150528
7	0.584639	-1.680239	1.204411
6	-2.811118	-1.544210	0.007400
6	-3.504634	-1.523996	-1.200288
6	-3.517914	-1.464652	1.204833
6	-4.884470	-1.426614	-1.207006
1	-2.951830	-1.587539	-2.126679
6	-4.897719	-1.367610	1.191537
1	-2.975308	-1.481086	2.139208
6	-5.584453	-1.348382	-0.012723
1	-5.416455	-1.413181	-2.149474
1	-5.439875	-1.307460	2.126329
1	-6.664355	-1.273147	-0.020528
6	2.698478	-1.616770	0.038881
6	3.391178	-1.510406	1.242474
6	3.408067	-1.658631	-1.158802
6	4.773021	-1.447211	1.244947
1	2.836154	-1.479197	2.169176
6	4.789844	-1.595652	-1.149720
1	2.865997	-1.741417	-2.089961
6	5.475750	-1.489767	0.050527
1	5.304228	-1.364910	2.184325
1	5.334299	-1.630536	-2.084478
1	6.557182	-1.440911	0.055134
8	-0.373149	4.304021	0.499727

TS for the reaction of trans-5-oxyene with 3,6-diphenyl-s-tetrazine

M06L/6-311+G(d,p)

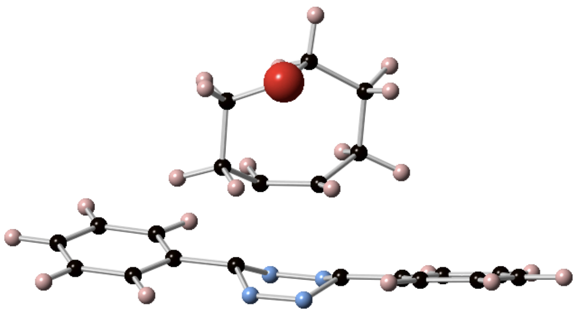

Electronic Energy-1107.71567284 au
Sum of electronic and zero-point Energies -1107.322021 au
Sum of electronic and thermal Energies -1107.301029 au
Sum of electronic and thermal Enthalpies -1107.300085 au
Sum of electronic and thermal Free Energies -1107.371819 au
Imaginary Frequency 457.5589i cm-1

Atomic Coordinates in Angstroms.

Atomic Number	X	Y	Z
6	0.222994	3.689442	-0.680594
6	-1.450496	1.456295	1.131238
6	1.406002	1.463096	-1.186463
6	-0.729488	0.839667	-0.028895
6	0.661013	0.918443	-0.015805
6	-1.427844	2.982202	0.942607
6	1.526767	2.991579	-1.025042
1	0.324097	4.770988	-0.841518
1	-0.944599	1.193356	2.066635
1	0.864396	1.227702	-2.110878
1	-1.200050	0.999602	-1.002077
1	1.131093	1.064894	0.956130
1	-0.588538	3.342814	-1.336923
1	-2.491658	1.127953	1.212517
1	2.410610	1.037848	-1.278197
1	-1.787374	3.474270	1.852189
1	1.930173	3.419648	-1.949726
1	-2.108246	3.265752	0.127091
1	2.252276	3.221321	-0.236820
7	0.668457	-1.625826	-1.137182
7	-0.682412	-1.583617	1.179821
6	1.259134	-1.254012	0.051681
6	-1.252887	-1.172335	-0.016556
7	0.598468	-1.593888	1.216670
7	-0.611747	-1.604330	-1.176414
6	2.724965	-1.166199	0.105632
6	3.361555	-0.898360	1.321769
6	3.495802	-1.308726	-1.053325
6	4.743394	-0.781841	1.378163
1	2.760364	-0.794834	2.219325
6	4.878890	-1.193391	-0.990696
1	2.996042	-1.522170	-1.992366
6	5.506486	-0.929129	0.222743
1	5.228699	-0.577119	2.327151
1	5.469839	-1.312494	-1.893440
1	6.587148	-0.839745	0.268856
6	-2.729993	-1.126987	-0.077031
6	-3.363573	-0.855476	-1.292256
6	-3.504368	-1.323414	1.069815

6	-4.748540	-0.784861	-1.361037
1	-2.758213	-0.718362	-2.183035
6	-4.890296	-1.255194	0.995903
1	-3.006370	-1.539815	2.008948
6	-5.516238	-0.985259	-0.217051
1	-5.232040	-0.578848	-2.310838
1	-5.484577	-1.416475	1.889893
1	-6.598962	-0.933381	-0.271628
8	-0.125056	3.474638	0.687807

CAM-B3LYP/tzvp
Electronic Energy-1107.32921540 au
Sum of electronic and zero-point Energies -1106.930187 au
Sum of electronic and thermal Energies -1106.909508 au
Sum of electronic and thermal Enthalpies -1106.908564 au
Sum of electronic and thermal Free Energies -1106.979908 au
Imaginary Frequency 427.8684i cm-1

Atomic Coordinates in Angstroms.

Atomic Number	X	Y	Z
6	0.274878	3.786212	-0.634125
6	-1.463610	1.519330	1.104231
6	1.417932	1.522382	-1.168792
6	-0.721503	0.908179	-0.043522
6	0.658722	0.940393	-0.015407
6	-1.453681	3.045701	0.910308
6	1.563872	3.040601	-0.950835
1	0.447703	4.859813	-0.750441
1	-0.973712	1.270898	2.046826
1	0.881954	1.330075	-2.100911
1	-1.188710	1.036752	-1.015577
1	1.114758	1.095636	0.956181
1	-0.515933	3.506672	-1.338044
1	-2.501339	1.188415	1.161911
1	2.413856	1.090739	-1.265753
1	-1.853403	3.525818	1.804995
1	2.005942	3.482668	-1.847940
1	-2.105547	3.313900	0.071849
1	2.265067	3.225572	-0.133864
7	0.656451	-1.550216	-1.150788
7	-0.677056	-1.528756	1.162334
6	1.240692	-1.184961	0.033435
6	-1.262683	-1.188987	-0.031243
7	0.591076	-1.518762	1.196554
7	-0.612112	-1.556623	-1.185493
6	2.718295	-1.141162	0.087022
6	3.362107	-0.889600	1.294883
6	3.477979	-1.339905	-1.061870
6	4.743490	-0.836435	1.351389
1	2.771801	-0.754642	2.190836
6	4.860392	-1.289123	-0.999939
1	2.975293	-1.549036	-1.995644
6	5.497032	-1.035266	0.204444
1	5.234708	-0.643117	2.296368
1	5.442796	-1.452444	-1.897652
1	6.577840	-0.996058	0.250611
6	-2.742525	-1.169485	-0.084280
6	-3.391614	-0.979901	-1.300386
6	-3.497245	-1.328101	1.073785
6	-4.773926	-0.947446	-1.356009

1	-2.804421	-0.879392	-2.202954
6	-4.880308	-1.298151	1.013068
1	-2.989995	-1.489658	2.014475
6	-5.522646	-1.105341	-0.199671
1	-5.269524	-0.803903	-2.307603
1	-5.458814	-1.430011	1.918432
1	-6.603973	-1.083013	-0.244869
8	-0.155262	3.565156	0.706465

Product of the reaction of trans-5-oxyene with 3,6-diphenyl-s-tetrazine
M06L/6-311+G(d,p)

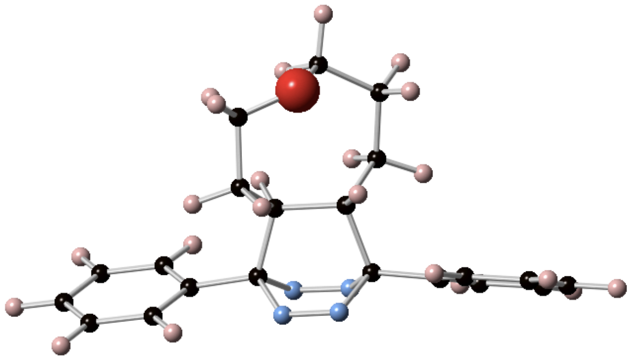

Electronic Energy-1107.75822402 au
Sum of electronic and zero-point Energies -1107.360686 au
Sum of electronic and thermal Energies -1107.340409 au
Sum of electronic and thermal Enthalpies -1107.339464 au
Sum of electronic and thermal Free Energies -1107.409189 au

Atomic Coordinates in Angstroms.

Atomic Number	X	Y	Z
6	0.290160	3.690844	-0.639344
6	1.368925	1.428331	-1.217104
6	-1.398276	1.452050	1.167692
6	0.751752	0.689976	-0.023270
6	-0.782963	0.685326	-0.001883
6	1.545520	2.928766	-1.010291
6	-1.369689	2.955667	0.956825
1	0.755575	1.240597	-2.108072
1	-0.869497	1.193434	2.092401
1	1.131356	1.128880	0.906078
1	-1.167684	1.100653	-0.943560
1	2.352385	1.001903	-1.436874
1	-2.442668	1.152531	1.310385
1	1.959089	3.363110	-1.927751
1	-1.748775	3.464161	1.850326
1	2.291814	3.108316	-0.226987
1	-2.034281	3.231526	0.124489
7	-0.655227	-1.416225	1.191559
7	0.625542	-1.432995	-1.180415
6	-1.254164	-0.816581	-0.009690
6	1.223453	-0.814669	0.010663
7	-0.614933	-1.425611	-1.201002
7	0.585028	-1.405152	1.210900
6	-2.742763	-0.996770	-0.067349
6	-3.404496	-0.778402	-1.276452

6	-3.483130	-1.341114	1.063446
6	-4.786114	-0.902411	-1.355085
1	-2.826631	-0.531130	-2.161916
6	-4.866197	-1.466072	0.982050
1	-2.966771	-1.518861	2.000675
6	-5.521072	-1.246412	-0.224751
1	-5.289643	-0.736454	-2.302271
1	-5.433352	-1.738509	1.866386
1	-6.600126	-1.345389	-0.285798
6	2.712074	-0.992231	0.073986
6	3.374197	-0.714344	1.270563
6	3.451997	-1.391263	-1.038846
6	4.756005	-0.832304	1.354043
1	2.797135	-0.421819	2.142773
6	4.835450	-1.509312	-0.952762
1	2.934952	-1.615288	-1.965681
6	5.490860	-1.228907	0.240906
1	5.260023	-0.617903	2.291203
1	5.402468	-1.823312	-1.823419
1	6.570311	-1.321416	0.305555
1	0.454934	4.770468	-0.758196
1	-0.542800	3.419214	-1.304976
8	-0.062234	3.441253	0.717116

S14. NMR spectra

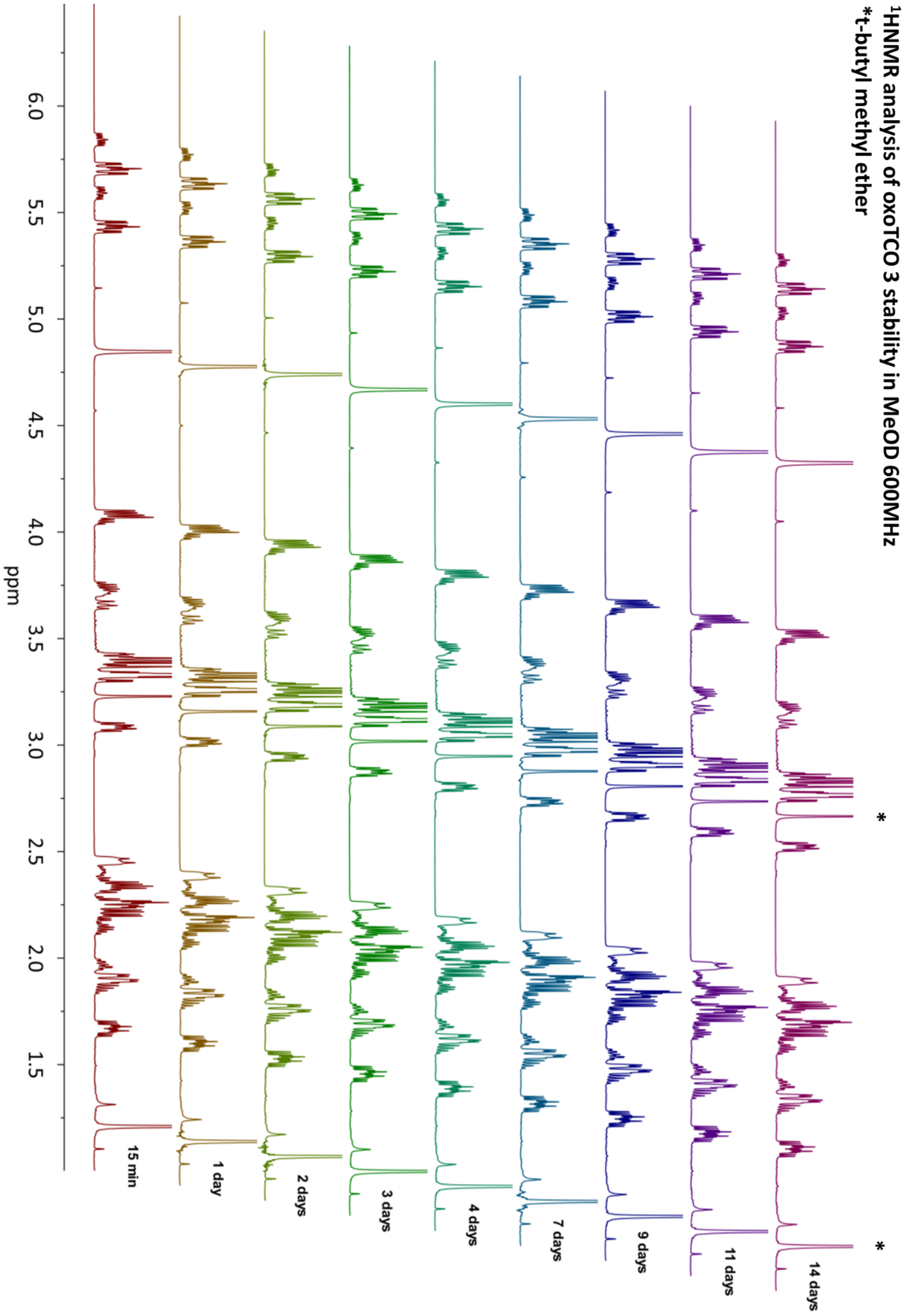

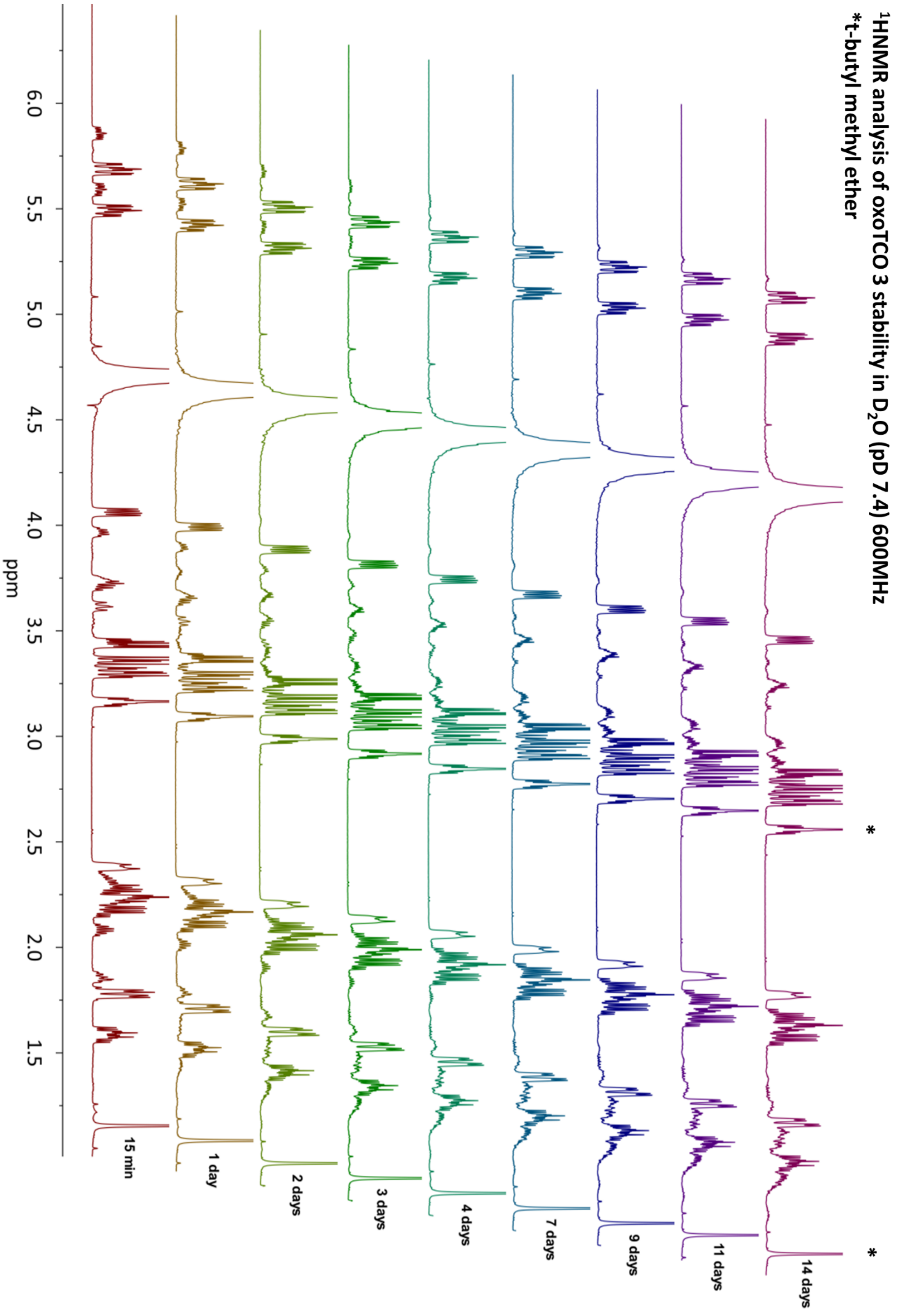

^{1}HNMR analysis of oxoTCO 3 stability in D_2O (pD 7.4) 600MHz
*t-butyl methyl ether
*
*
15 min
1 day
2 days
3 days
4 days
7 days
9 days
11 days
14 days
6.0
5.5
5.0
4.5
4.0
3.5
3.0
2.5
2.0
1.5
ppm

^{1}HNMR analysis of oxoTCO 3 stability in MeOD and mercaptoethanol 600MHz

*t-butyl methyl ether

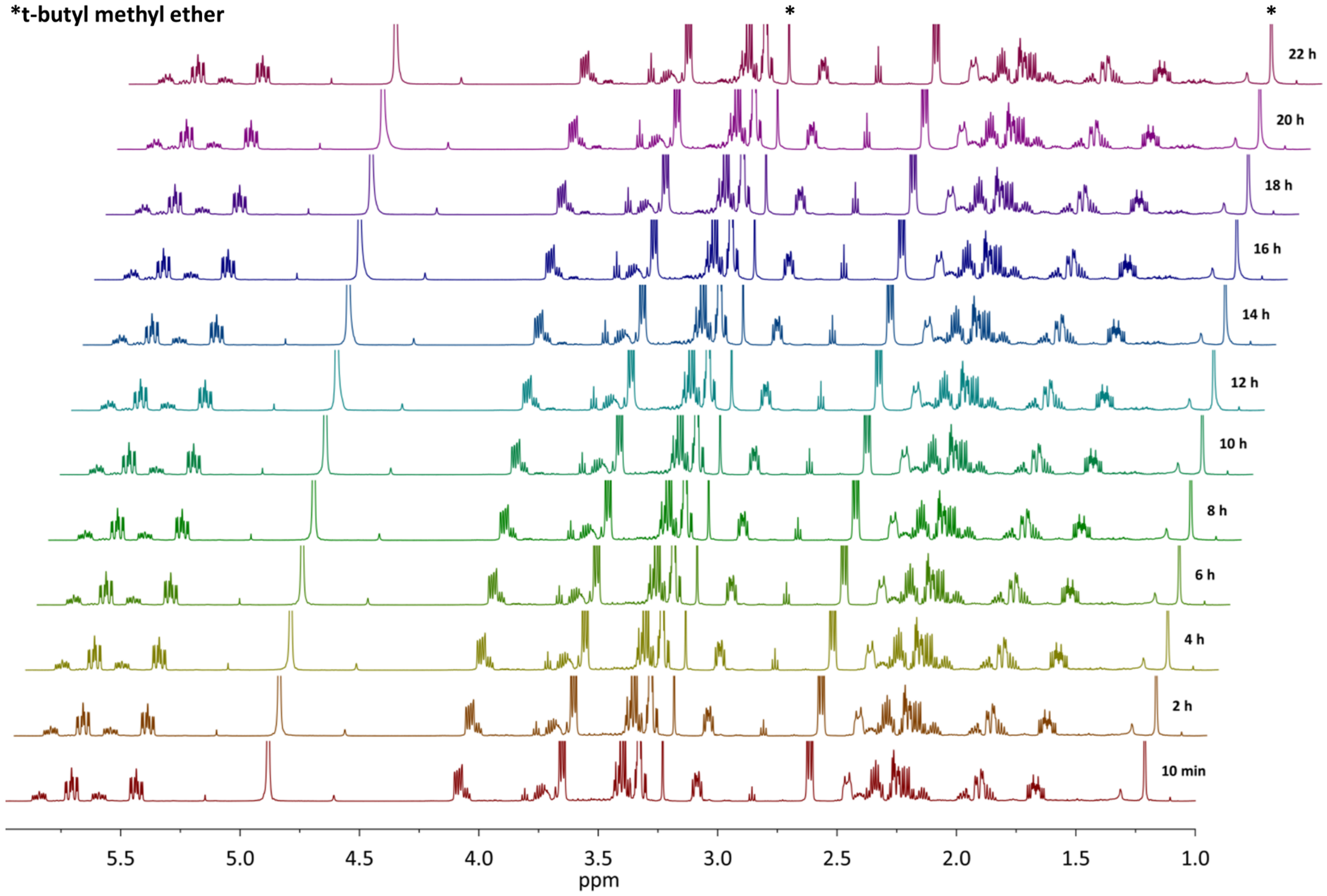

^{1}HNMR analysis of oxoTCO 3 stability in D_2O (pD 7.4) and mercaptoethanol 600MHz
*t-butyl methyl ether

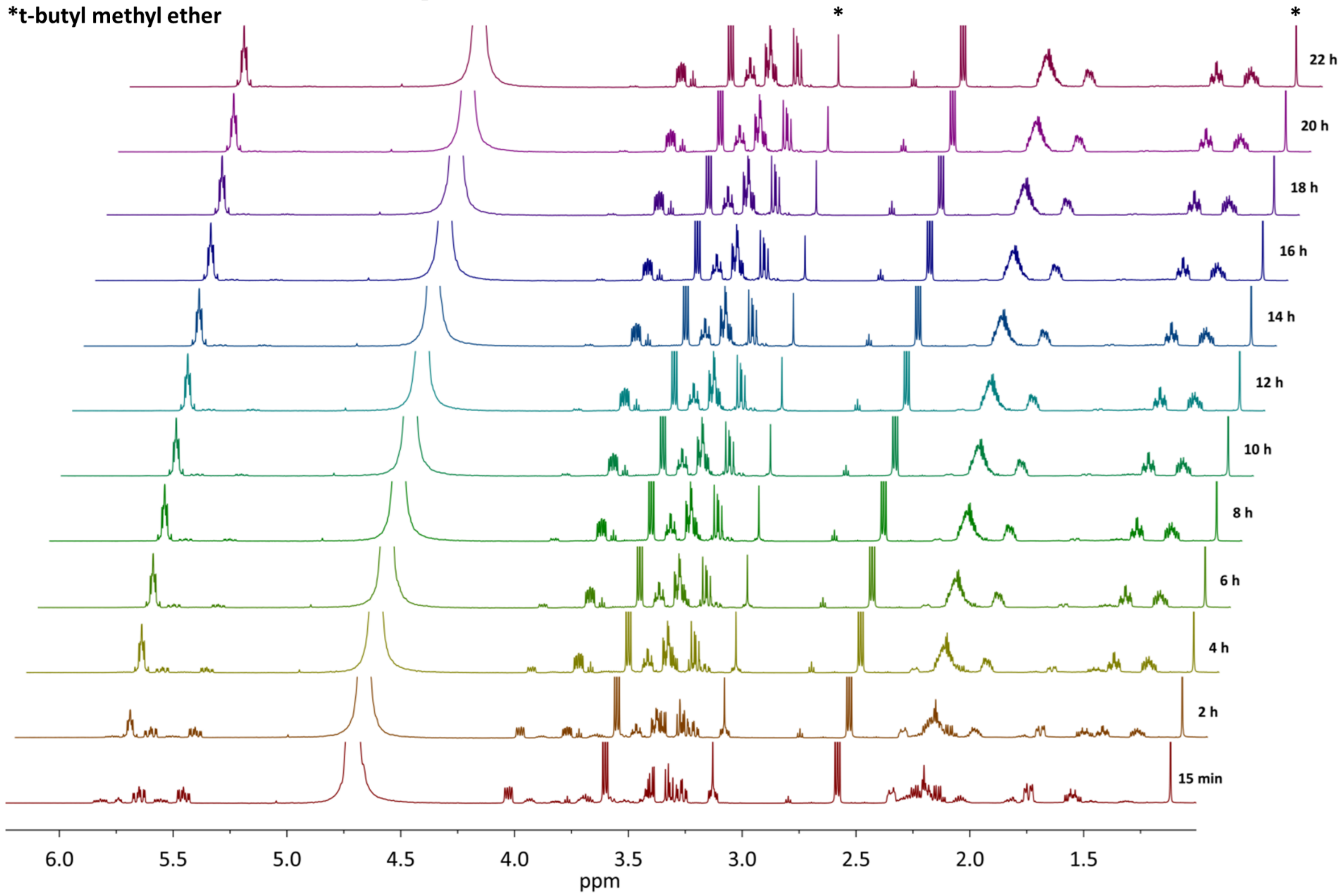

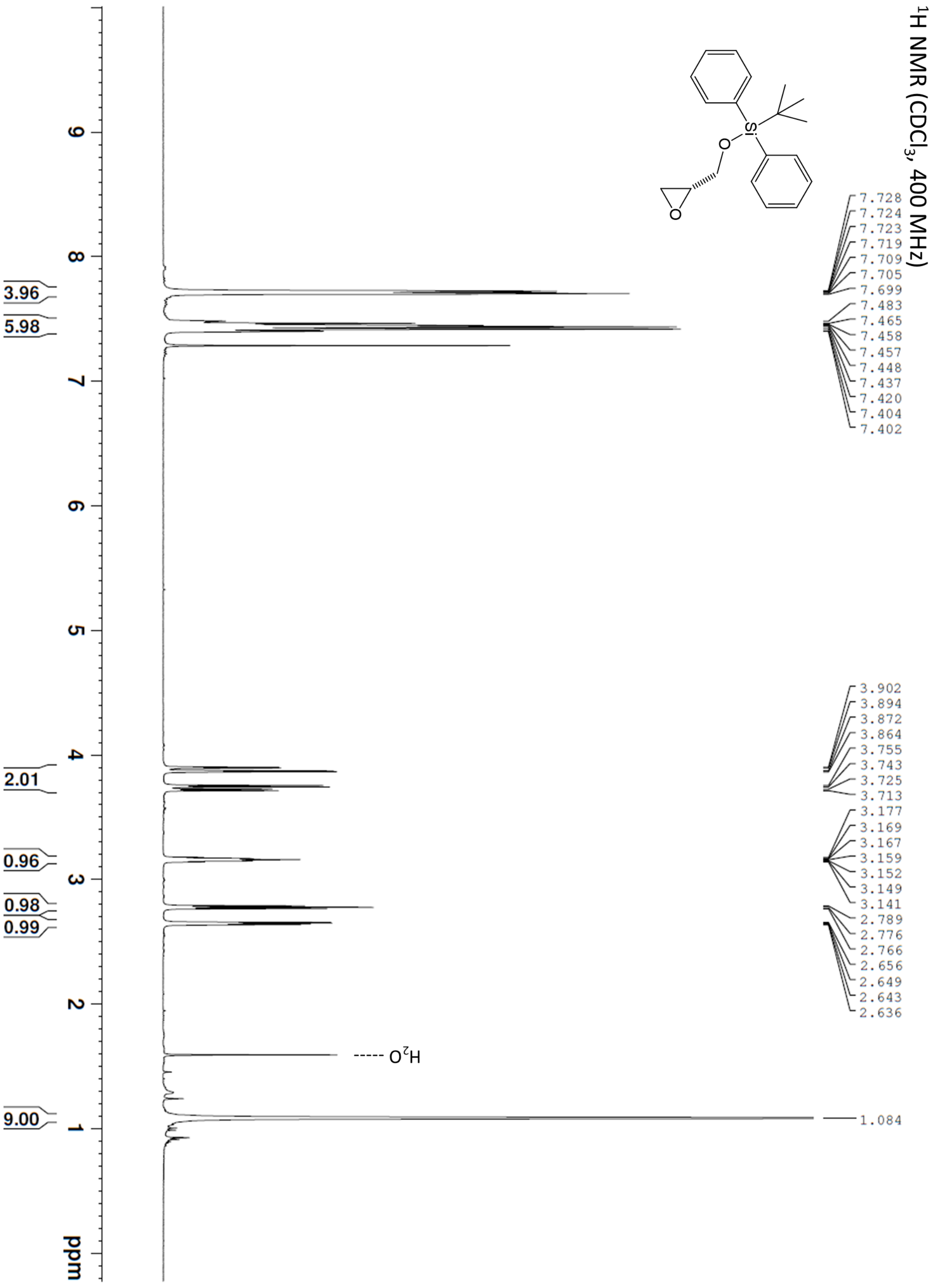

1H NMR (CDCl3, 400 MHz)
7.728
7.724
7.723
7.719
7.709
7.705
7.699
7.483
7.465
7.458
7.457
7.448
7.437
7.420
7.404
7.402
3.902
3.894
3.872
3.864
3.755
3.743
3.725
3.713
3.177
3.169
3.167
3.159
3.152
3.149
3.141
2.789
2.776
2.766
2.656
2.649
2.643
2.636
1.084
H2O
3.96
5.98
2.01
0.96
0.98
0.99
9.00
ppm

^{13}C NMR ($CDCl_3$, 100 MHz)

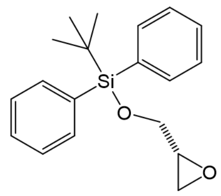

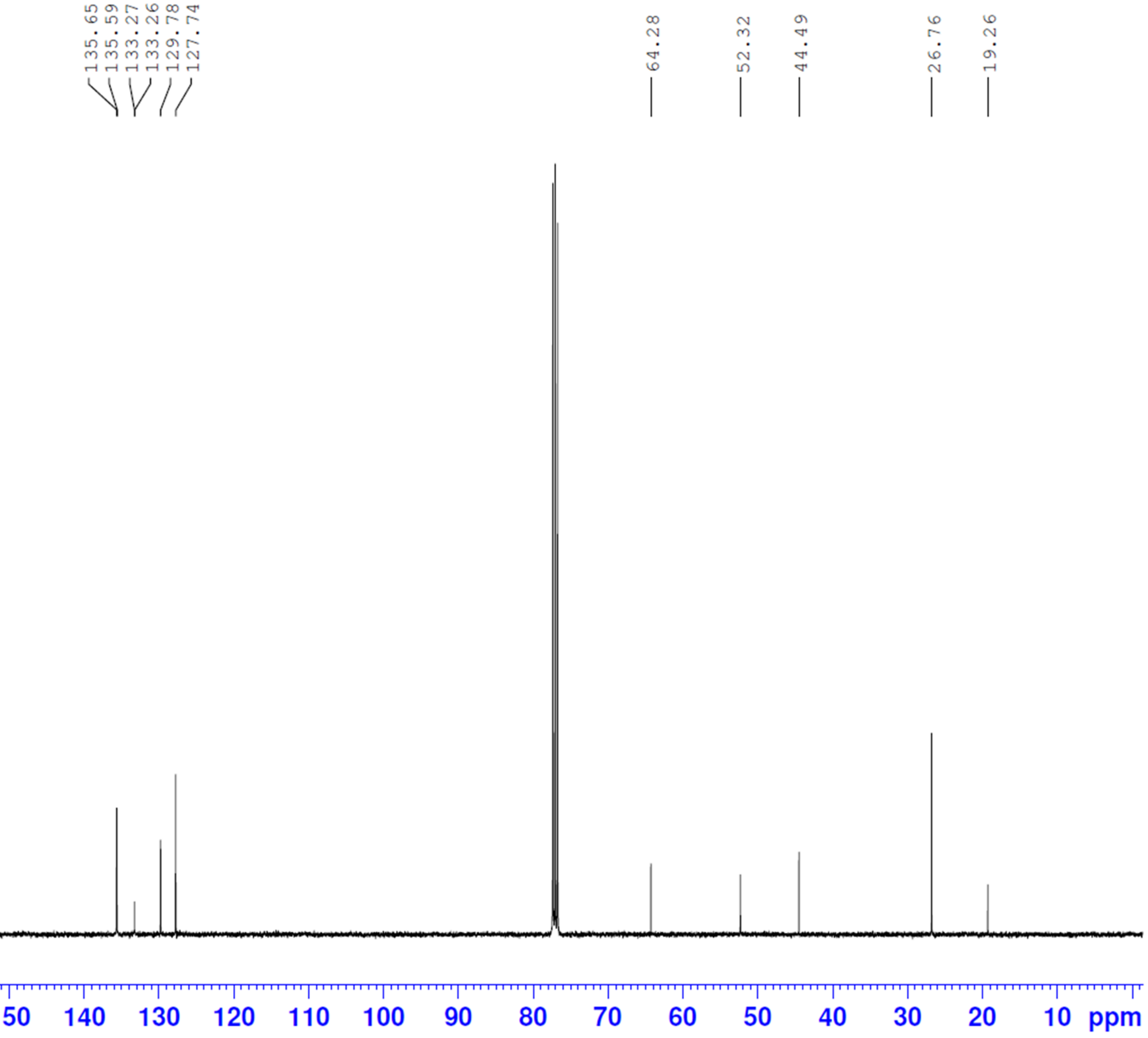

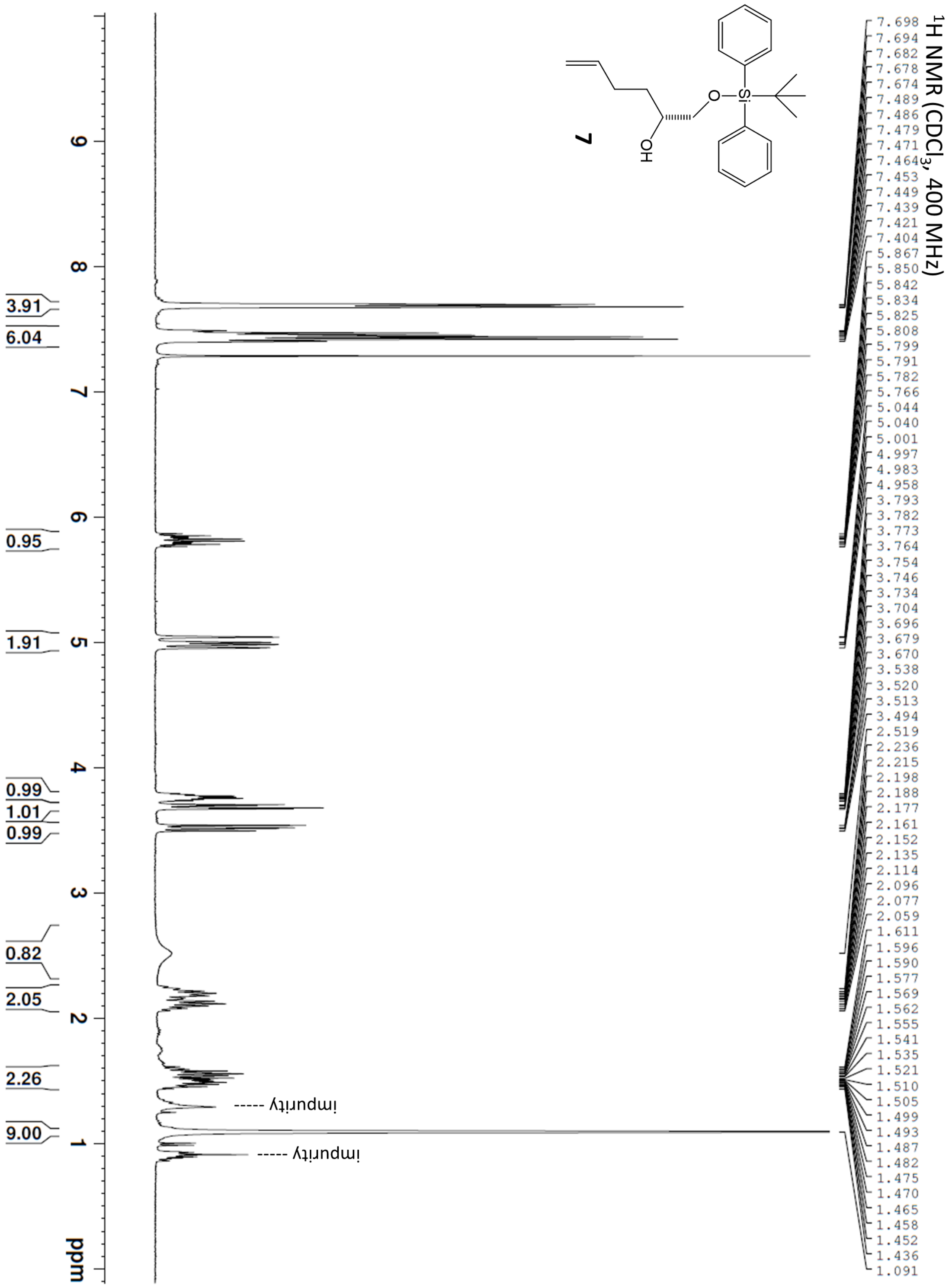

^{1}H NMR (CDCl$_3$, 400 MHz)
7
impurity
impurity
ppm

^{13}C NMR ($CDCl_3$, 100 MHz)

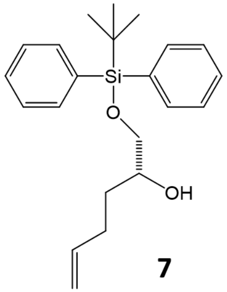

7

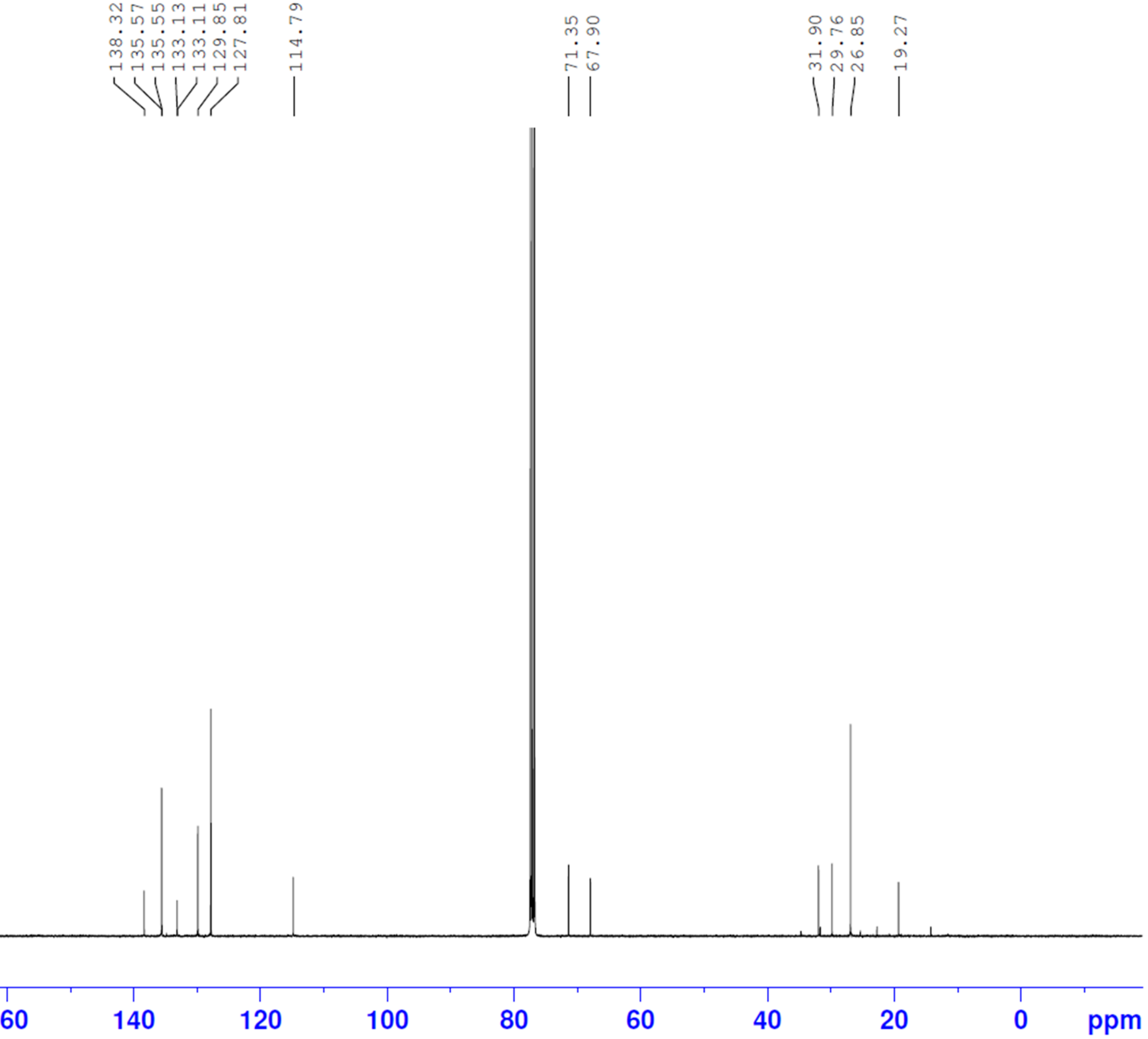

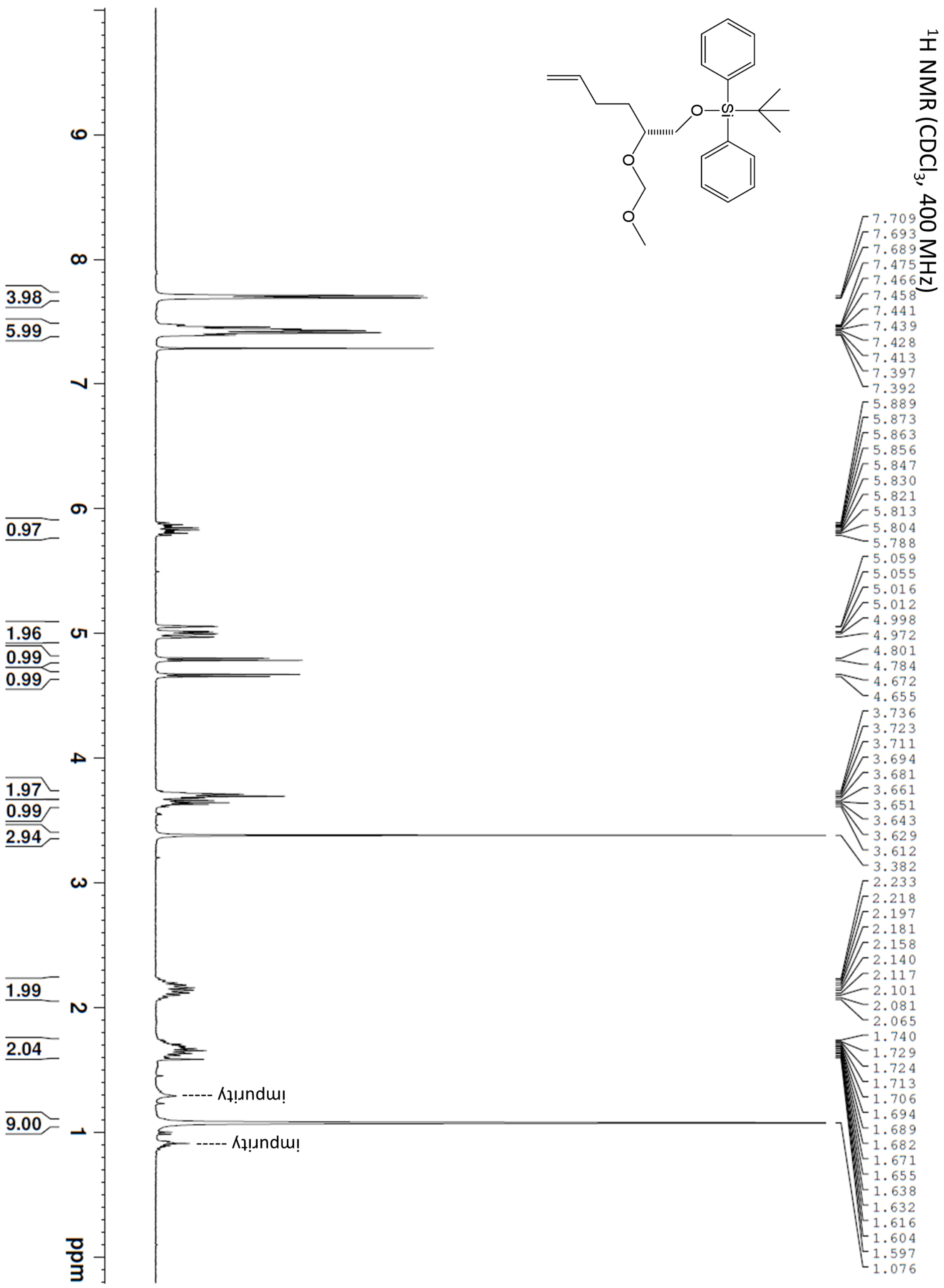
1H NMR (CDCl3, 400 MHz)
7.709
7.693
7.689
7.475
7.466
7.458
7.441
7.439
7.428
7.413
7.397
7.392
5.889
5.873
5.863
5.856
5.847
5.830
5.821
5.813
5.804
5.788
5.059
5.055
5.016
5.012
4.998
4.972
4.801
4.784
4.672
4.655
3.736
3.723
3.711
3.694
3.681
3.661
3.651
3.643
3.629
3.612
3.382
2.233
2.218
2.197
2.181
2.158
2.140
2.117
2.101
2.081
2.065
1.740
1.729
1.724
1.713
1.706
1.694
1.689
1.682
1.671
1.655
1.638
1.632
1.616
1.604
1.597
1.076
O
Si
O
O
impurity -----
impurity -----
3.98
5.99
0.97
1.96
0.99
0.99
1.97
0.99
2.94
1.99
2.04
9.00
9
8
7
6
5
4
3
2
1
ppm

^{13}C NMR (MeOD, 100 MHz)

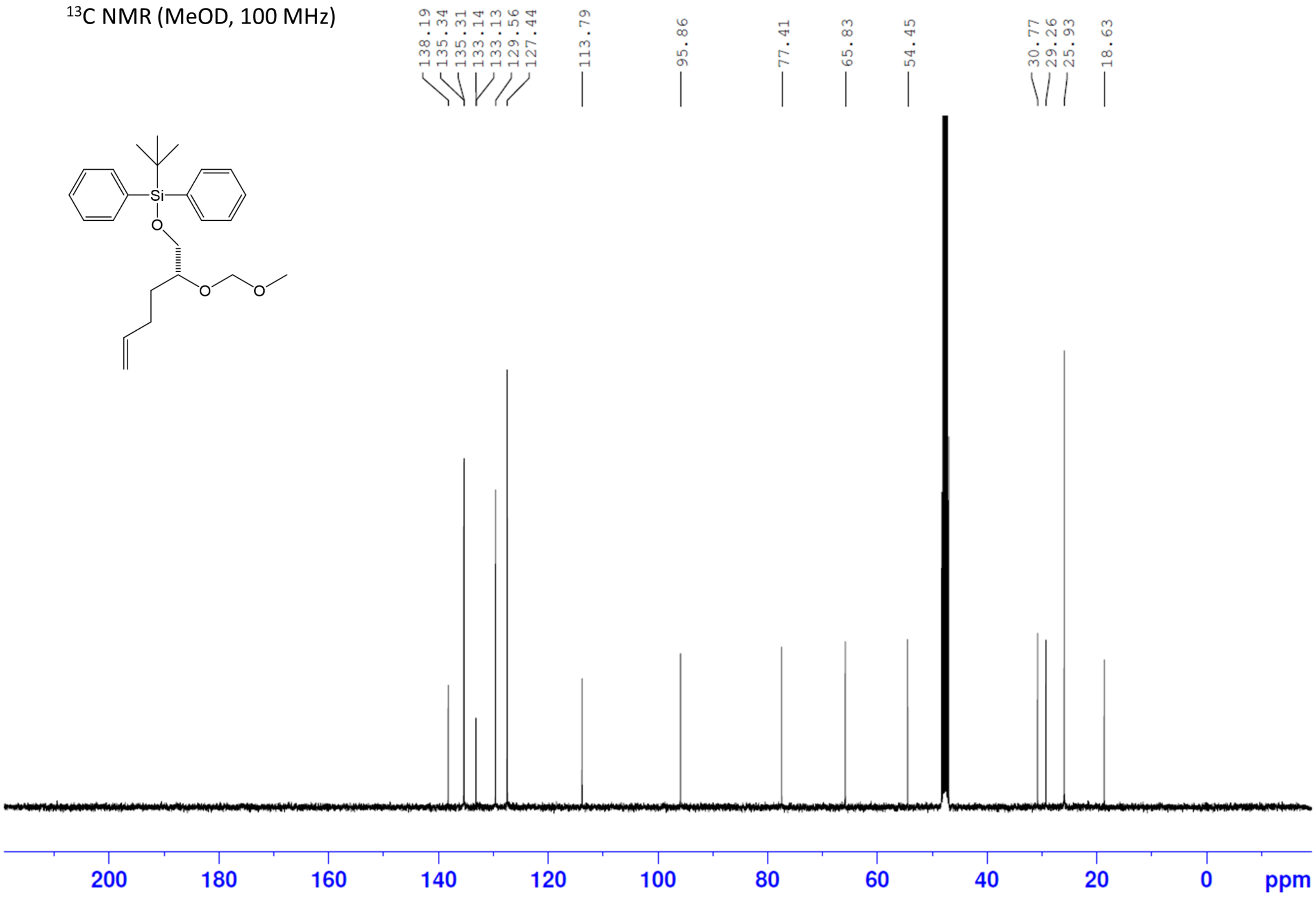

^1H NMR ($CDCl_3$, 400 MHz)

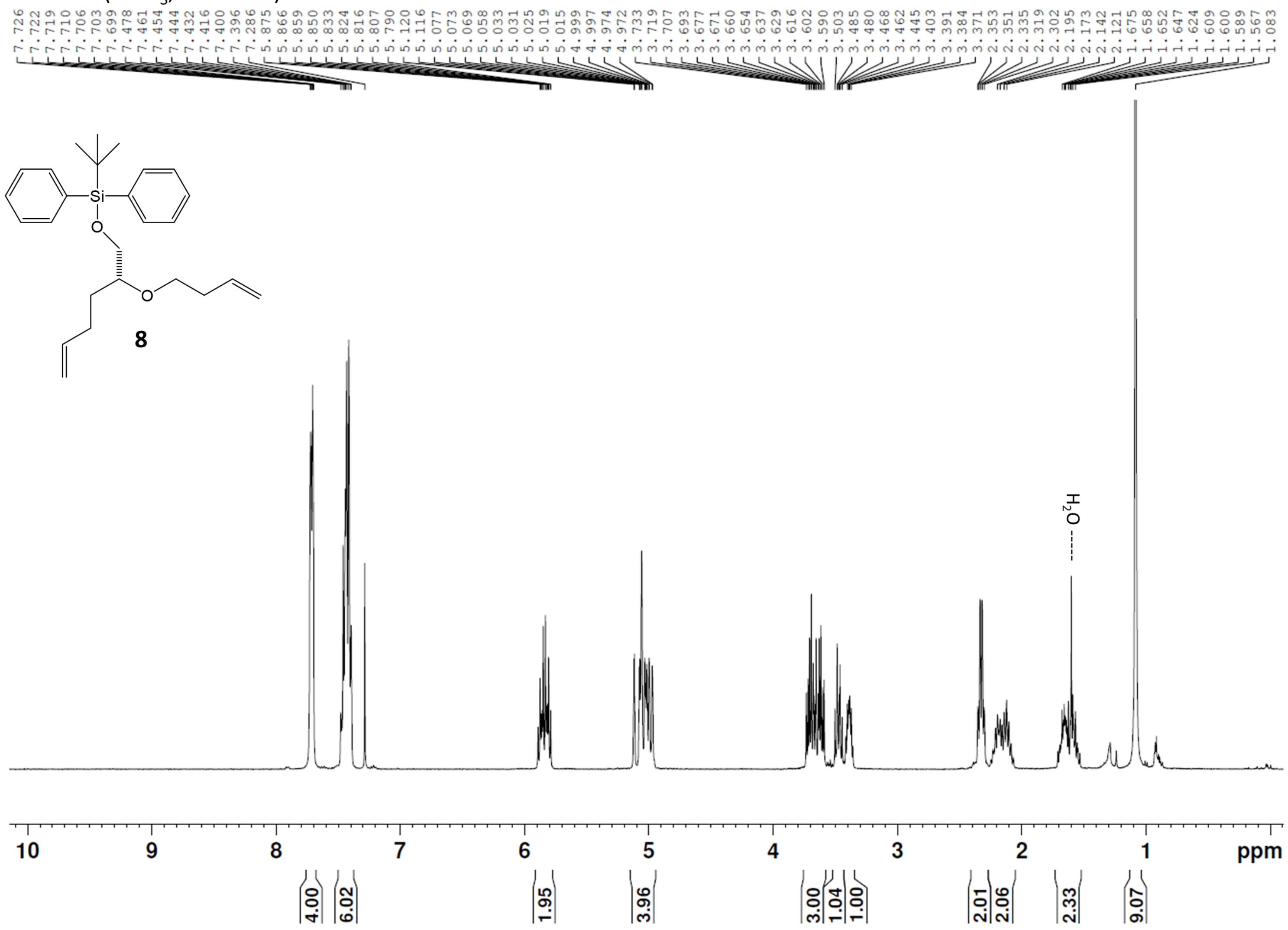

^{13}C NMR (CDCl$_3$, 100 MHz)

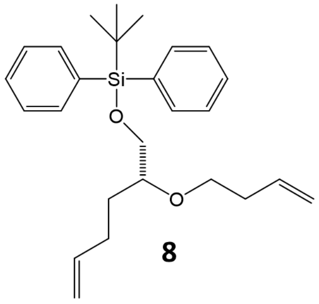

8

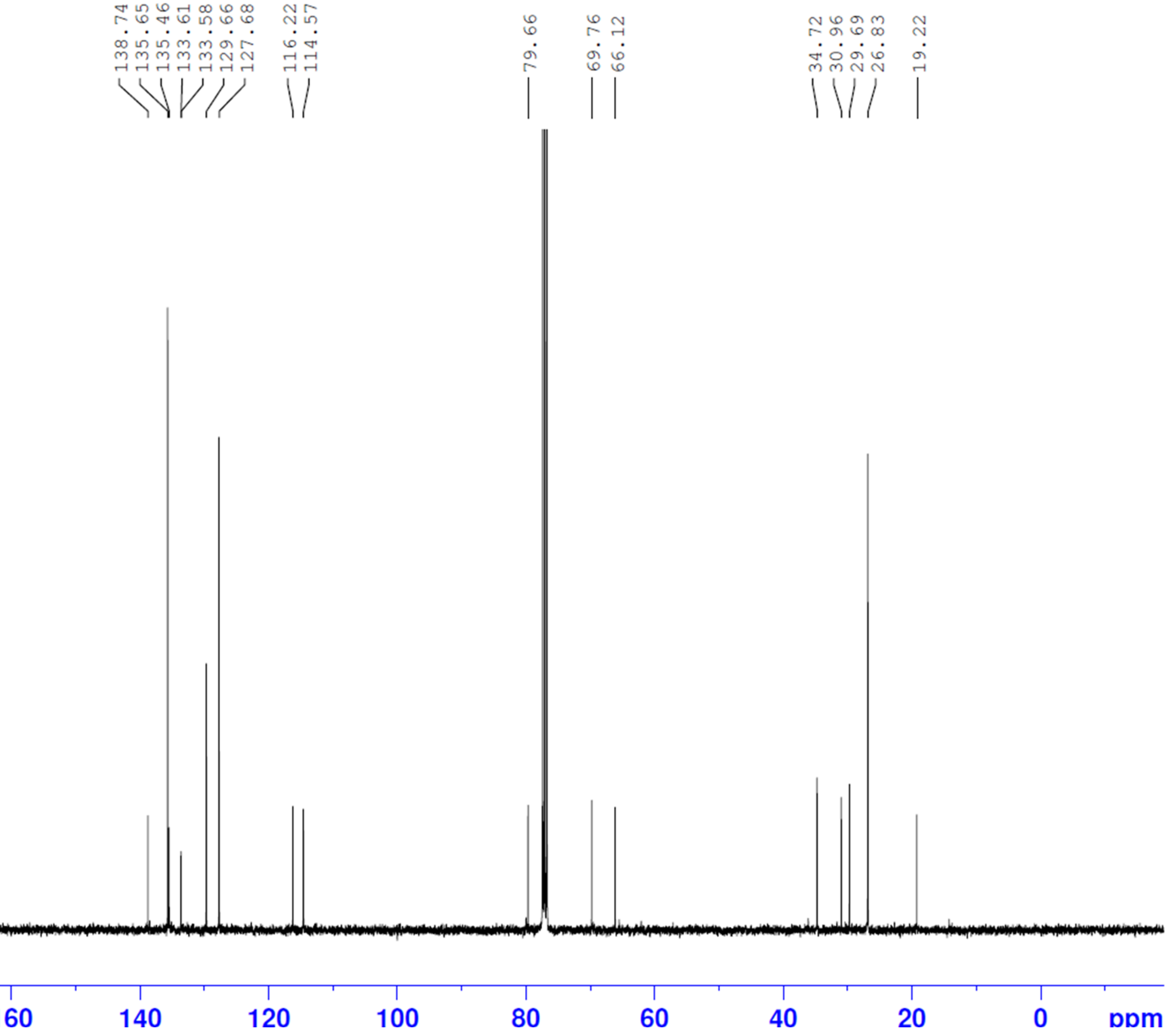

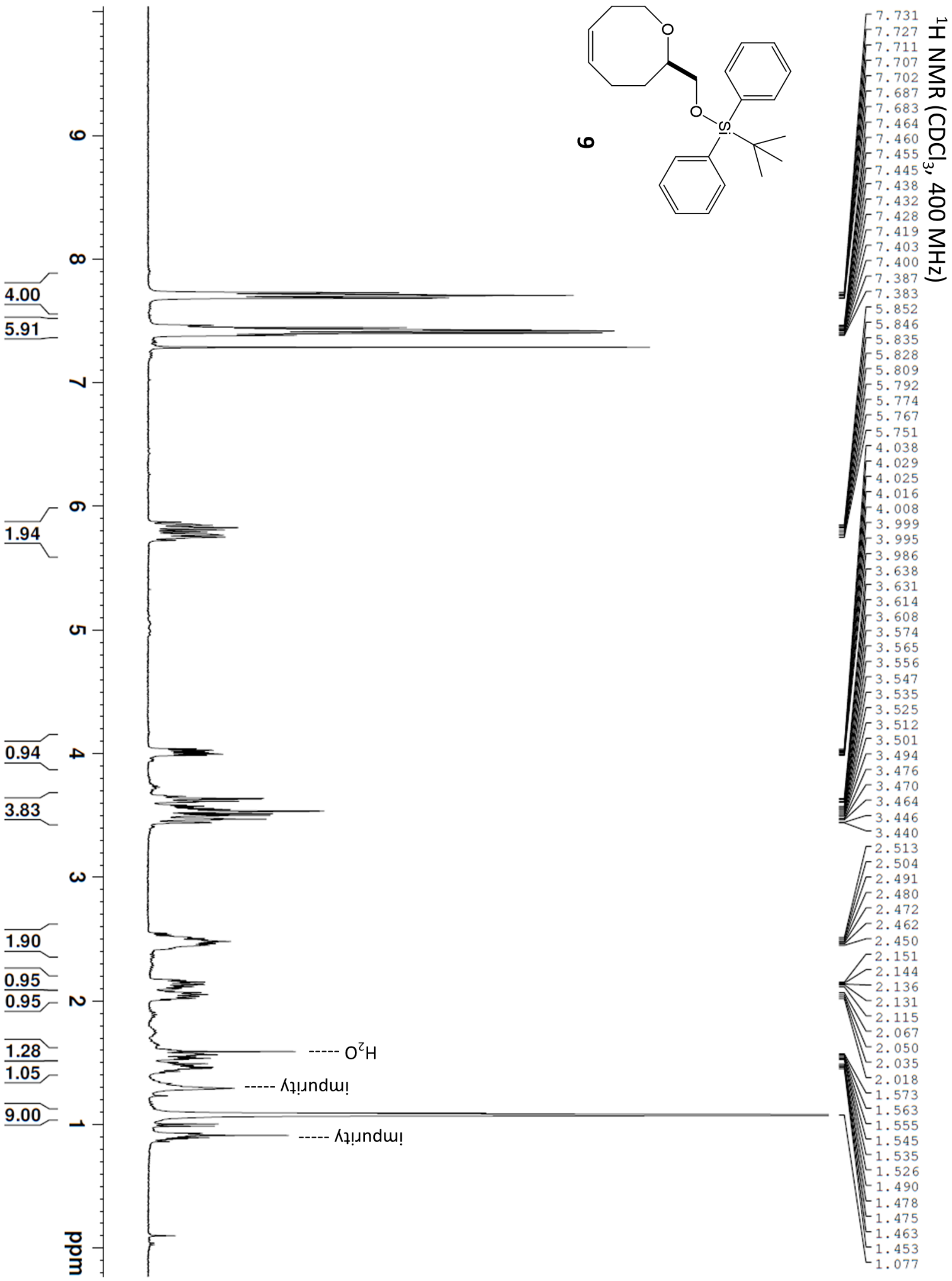

¹H NMR (CDCl₃, 400 MHz)
9
7.731 7.727 7.711 7.707 7.702 7.687 7.683 7.464 7.460 7.455 7.445 7.438 7.432 7.428 7.419 7.403 7.400 7.387 7.383 5.852 5.846 5.835 5.828 5.809 5.792 5.774 5.767 5.751 4.038 4.029 4.025 4.016 4.008 3.999 3.995 3.986 3.638 3.631 3.614 3.608 3.574 3.565 3.556 3.547 3.535 3.525 3.512 3.501 3.494 3.476 3.470 3.464 3.446 3.440 2.513 2.504 2.491 2.480 2.472 2.462 2.450 2.151 2.144 2.136 2.131 2.115 2.067 2.050 2.035 2.018 1.573 1.563 1.555 1.545 1.535 1.526 1.490 1.478 1.475 1.463 1.453 1.077
H₂O -----
impurity -----
impurity -----
4.00
5.91
1.94
0.94
3.83
1.90
0.95
0.95
1.28
1.05
9.00
9
8
7
6
5
4
3
2
1
ppm

^{13}C NMR ($CDCl_3$, 100 MHz)

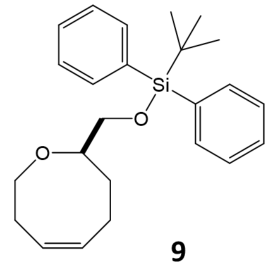

9

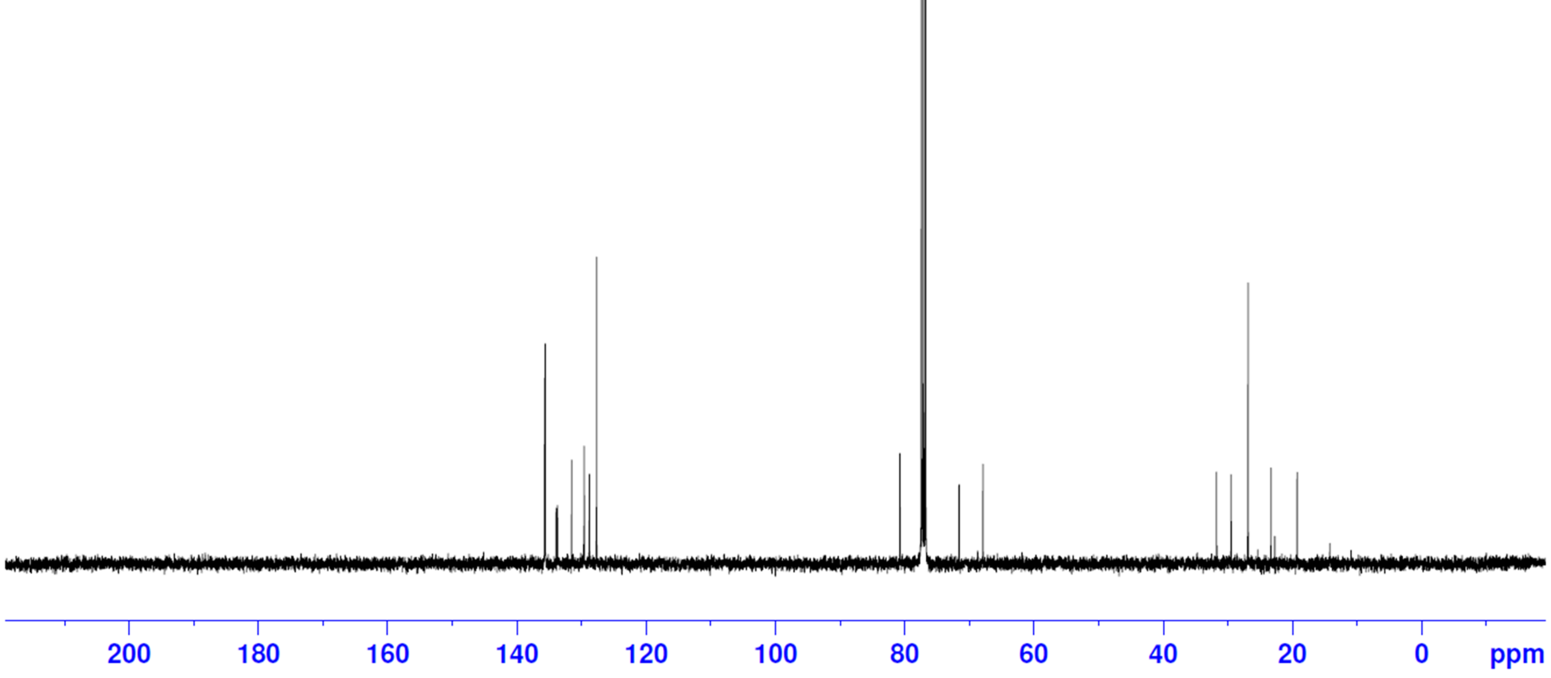

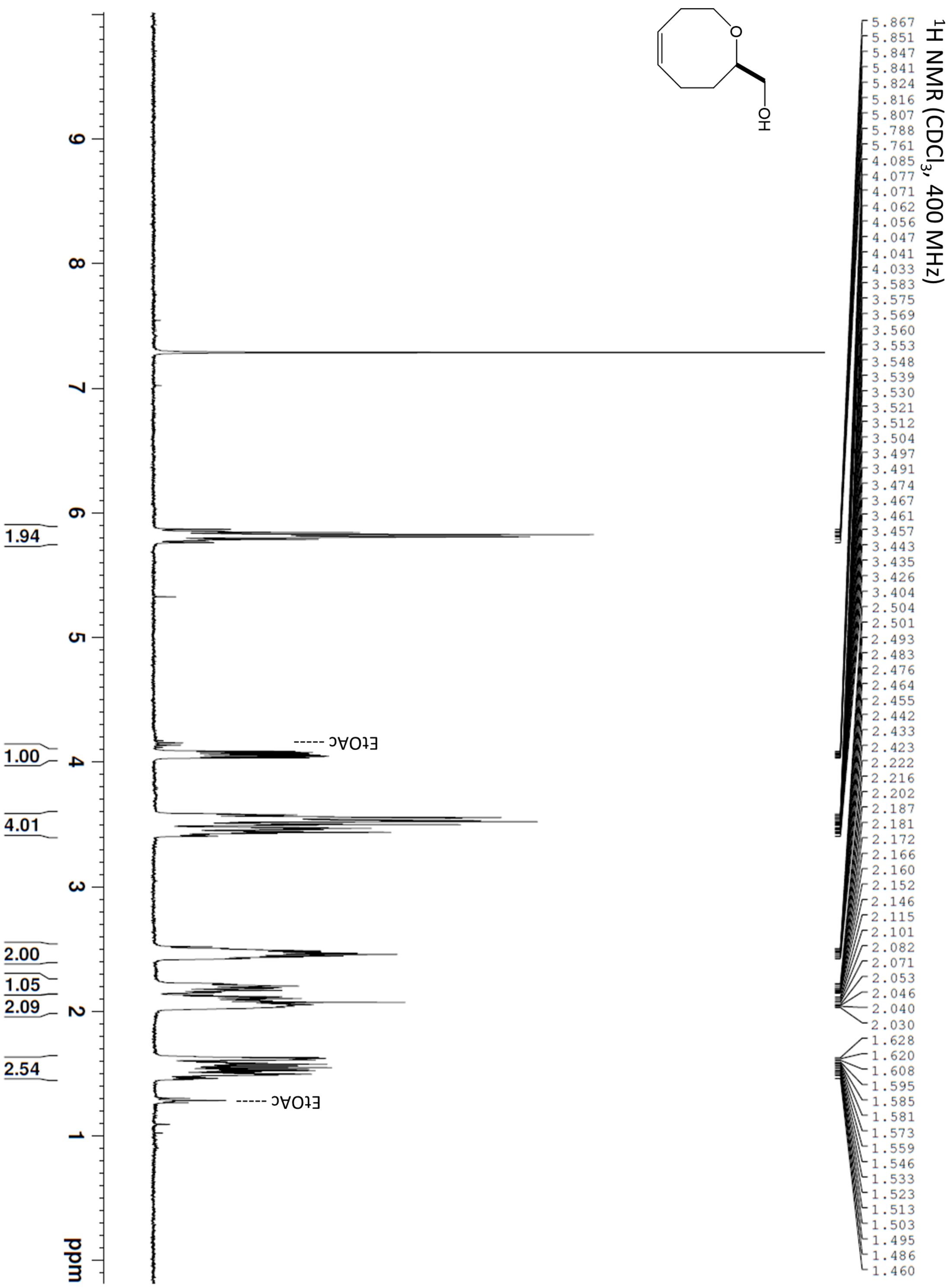

^1H NMR ($CDCl_3$, 400 MHz)
OH
O
5.867
5.851
5.847
5.841
5.824
5.816
5.807
5.788
5.761
4.085
4.077
4.071
4.062
4.056
4.047
4.041
4.033
3.583
3.575
3.569
3.560
3.553
3.548
3.539
3.530
3.521
3.512
3.504
3.497
3.491
3.474
3.467
3.461
3.457
3.443
3.435
3.426
3.404
2.504
2.501
2.493
2.483
2.476
2.464
2.455
2.442
2.433
2.423
2.222
2.216
2.202
2.187
2.181
2.172
2.166
2.160
2.152
2.146
2.115
2.101
2.082
2.071
2.053
2.046
2.040
2.030
1.628
1.620
1.608
1.595
1.585
1.581
1.573
1.559
1.546
1.533
1.523
1.513
1.503
1.495
1.486
1.460
EtOAc
EtOAc
1.94
1.00
4.01
2.00
1.05
2.09
2.54
9
8
7
6
5
4
3
2
1
ppm

^{13}C NMR ($CDCl_3$, 100 MHz)

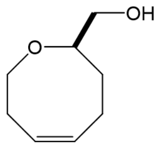

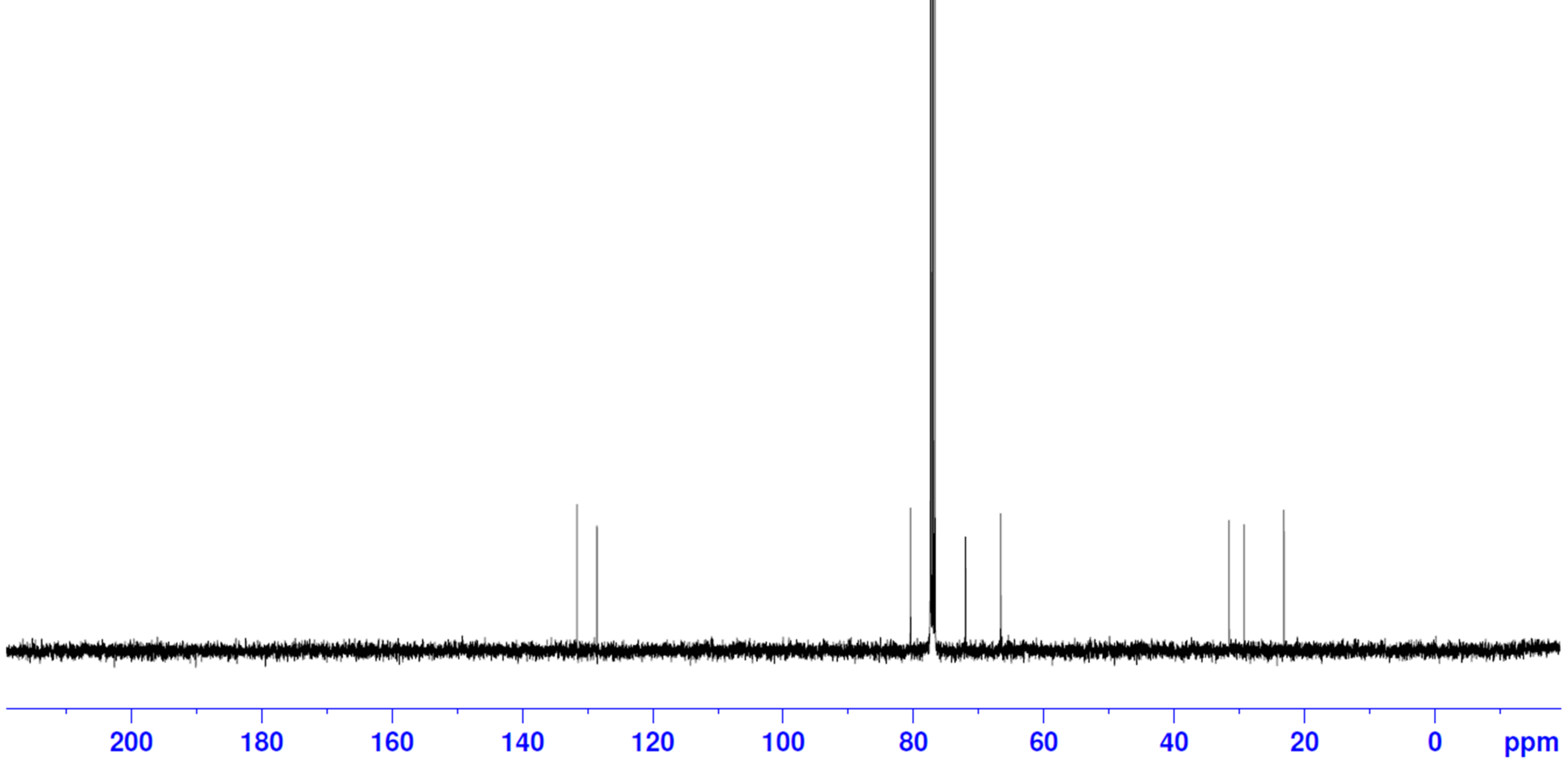

^1H NMR (C_6D_6, 400 MHz)

* Major diastereomer protons

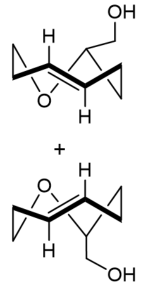

3

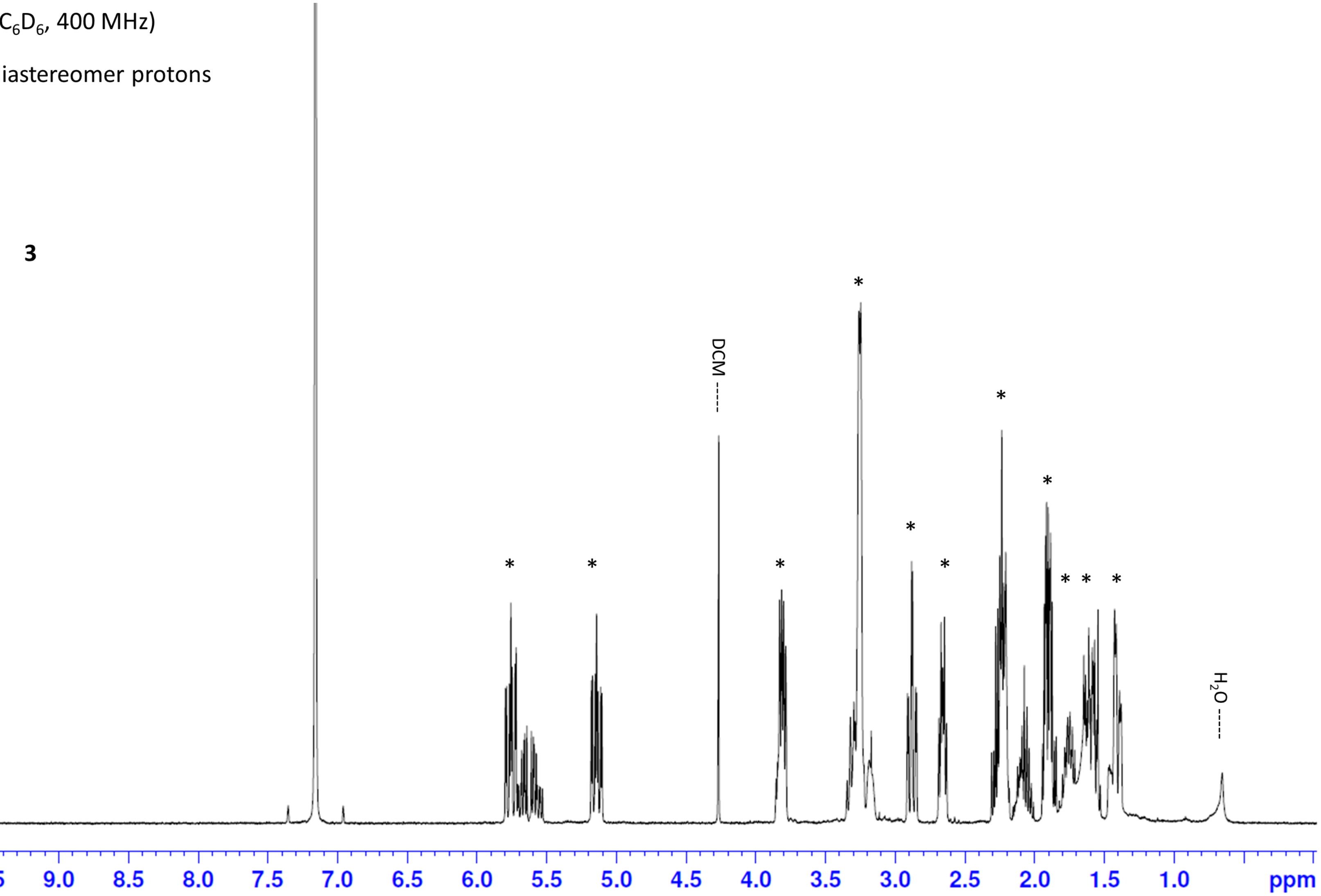

^{1}H NMR (C_6D_6, 400 MHz)

* Major diastereomer protons

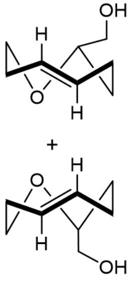

3

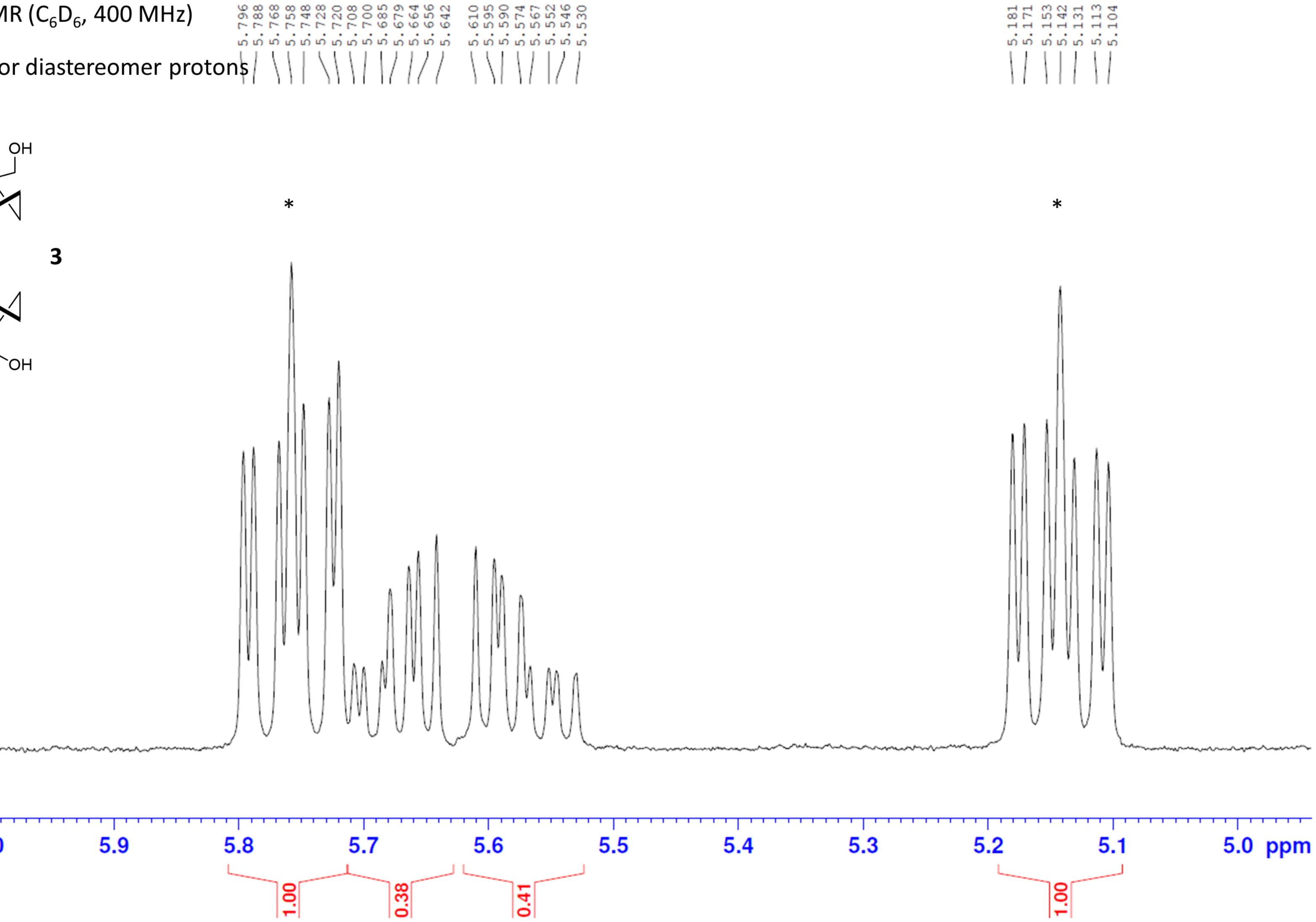

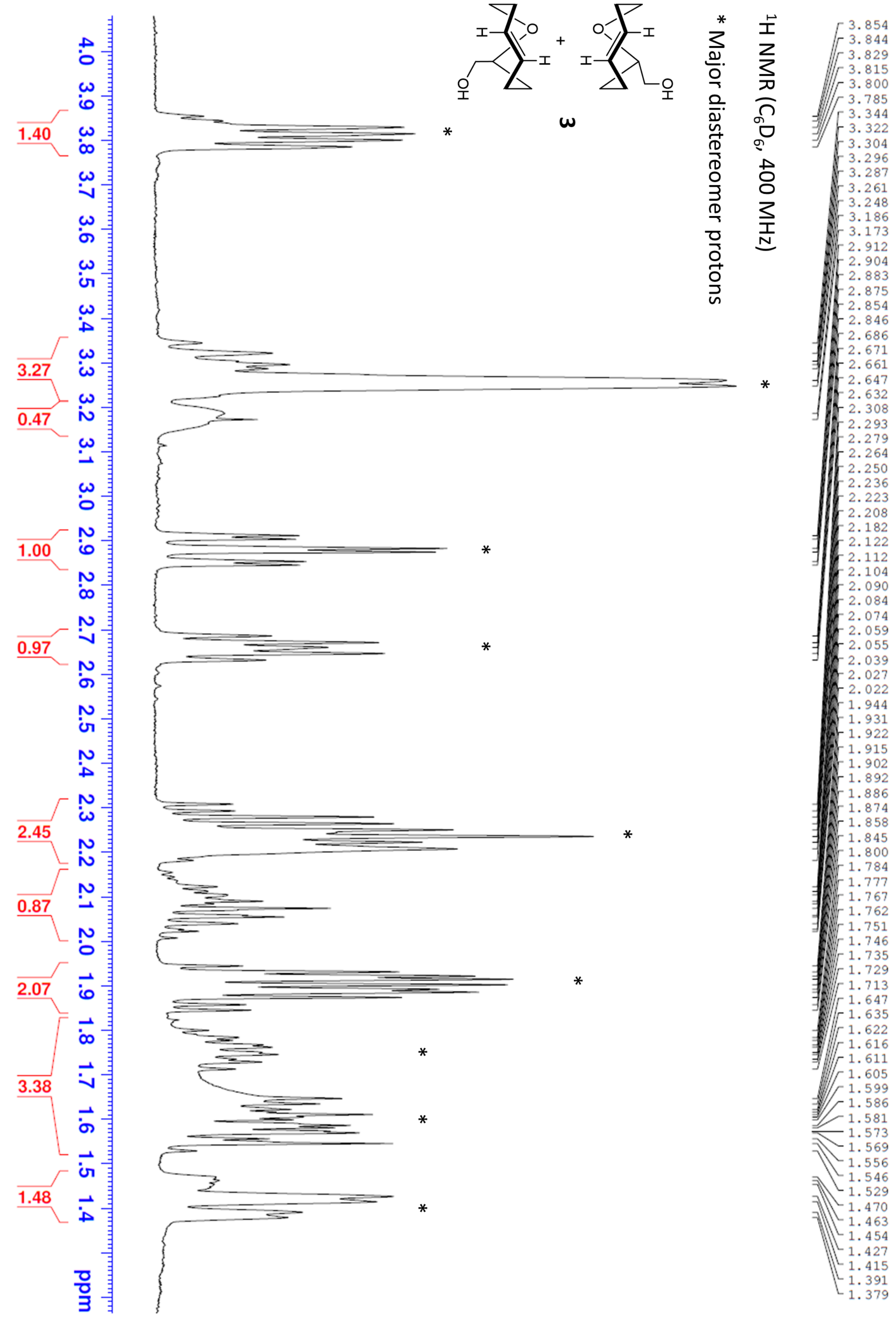

1H NMR (C6D6, 400 MHz)
* Major diastereomer protons
3
3.854 3.844 3.829 3.815 3.800 3.785 3.344 3.322 3.304 3.296 3.287 3.261 3.248 3.186 3.173 2.912 2.904 2.883 2.875 2.854 2.846 2.686 2.671 2.661 2.647 2.632 2.308 2.293 2.279 2.264 2.250 2.236 2.223 2.208 2.182 2.122 2.112 2.104 2.090 2.084 2.074 2.059 2.055 2.039 2.027 2.022 1.944 1.931 1.922 1.915 1.902 1.892 1.886 1.874 1.858 1.845 1.800 1.784 1.777 1.767 1.762 1.751 1.746 1.735 1.729 1.713 1.647 1.635 1.622 1.616 1.611 1.605 1.599 1.586 1.581 1.573 1.569 1.556 1.546 1.529 1.470 1.463 1.454 1.427 1.415 1.391 1.379
1.40
3.27
0.47
1.00
0.97
2.45
0.87
2.07
3.38
1.48
4.0 3.9 3.8 3.7 3.6 3.5 3.4 3.3 3.2 3.1 3.0 2.9 2.8 2.7 2.6 2.5 2.4 2.3 2.2 2.1 2.0 1.9 1.8 1.7 1.6 1.5 1.4 ppm

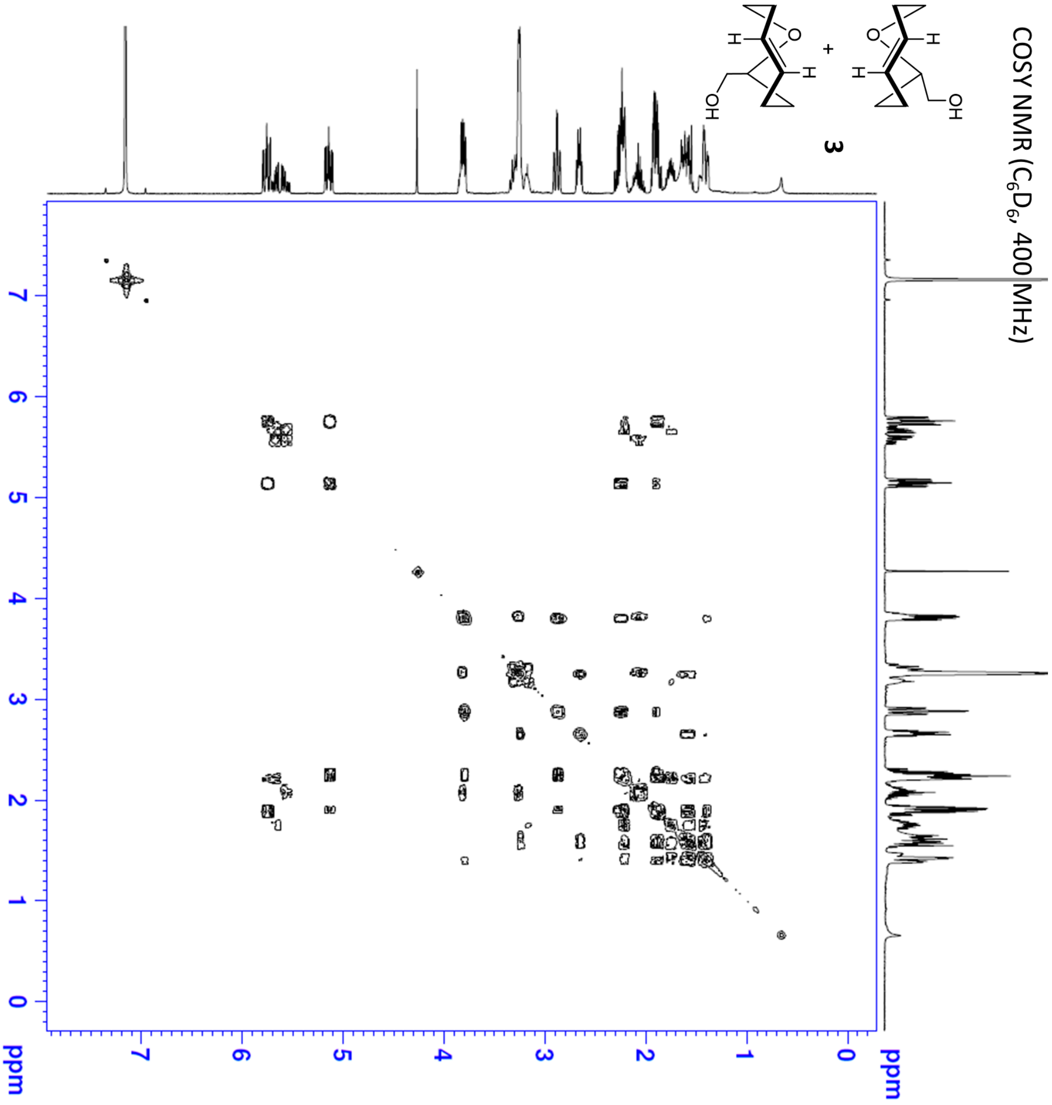

COSY NMR (C_6D_6, 400 MHz)

^{13}C NMR (C_6D_6, 100 MHz)

* Major diastereomer carbons

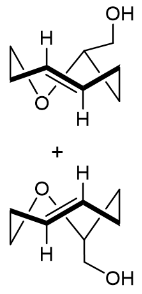

3

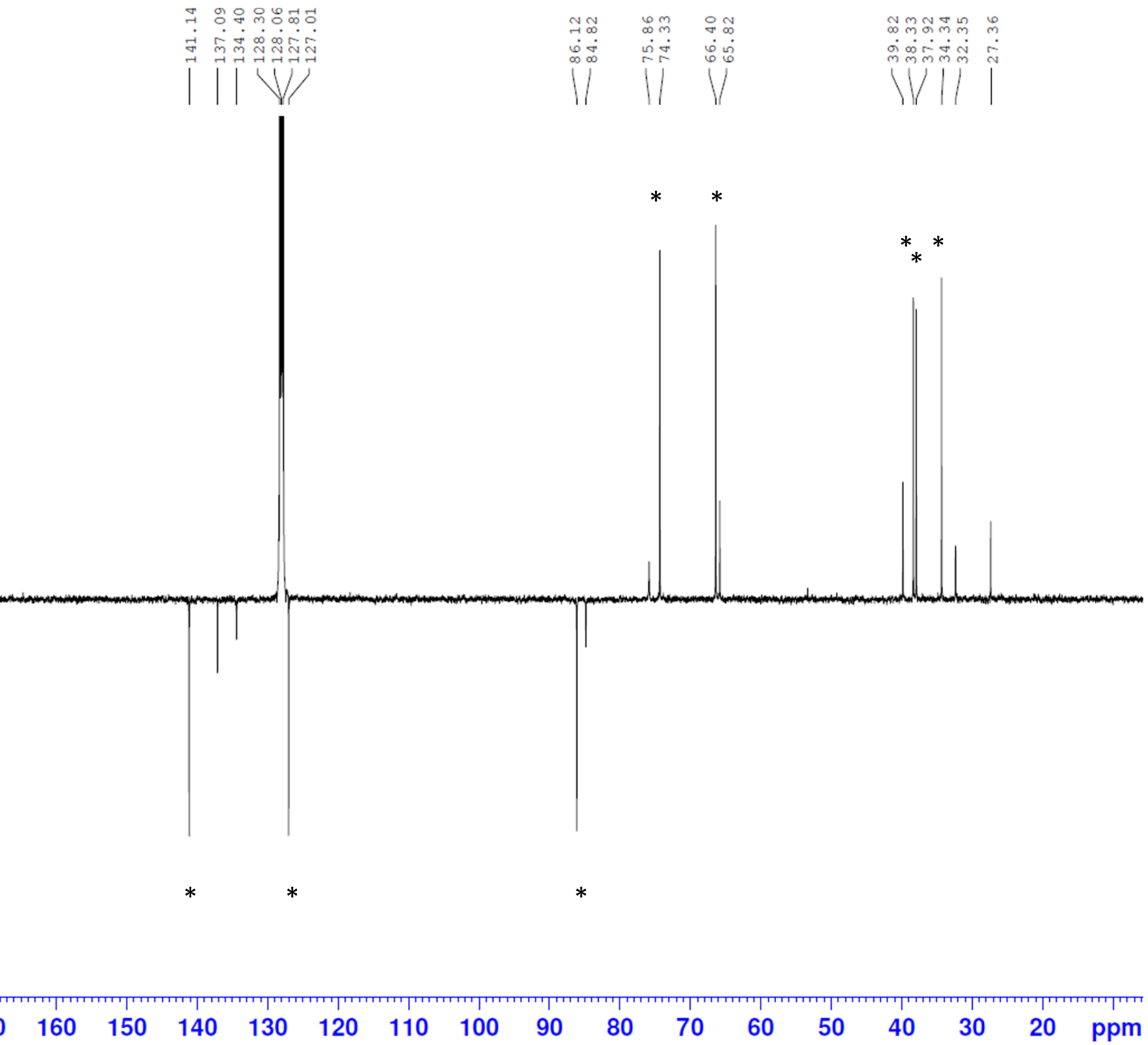

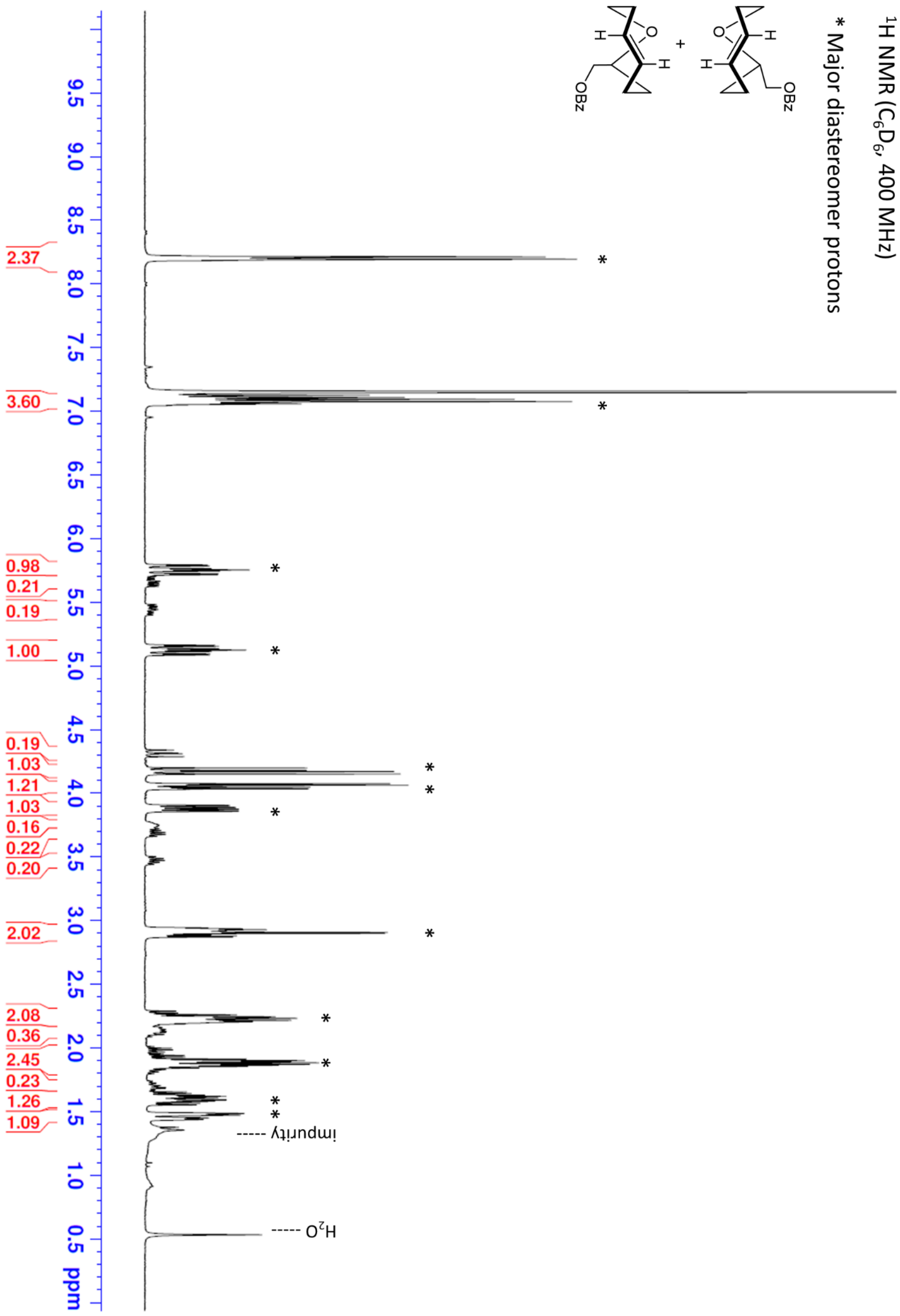

^1H NMR (C_6D_6, 400 MHz)
* Major diastereomer protons
OBz
OBz
impurity -----
H_2O -----
9.5 9.0 8.5 8.0 7.5 7.0 6.5 6.0 5.5 5.0 4.5 4.0 3.5 3.0 2.5 2.0 1.5 1.0 0.5 ppm
2.37 3.60 0.98 0.21 0.19 1.00 0.19 1.03 1.21 1.03 0.16 0.22 0.20 2.02 2.08 0.36 2.45 0.23 1.26 1.09

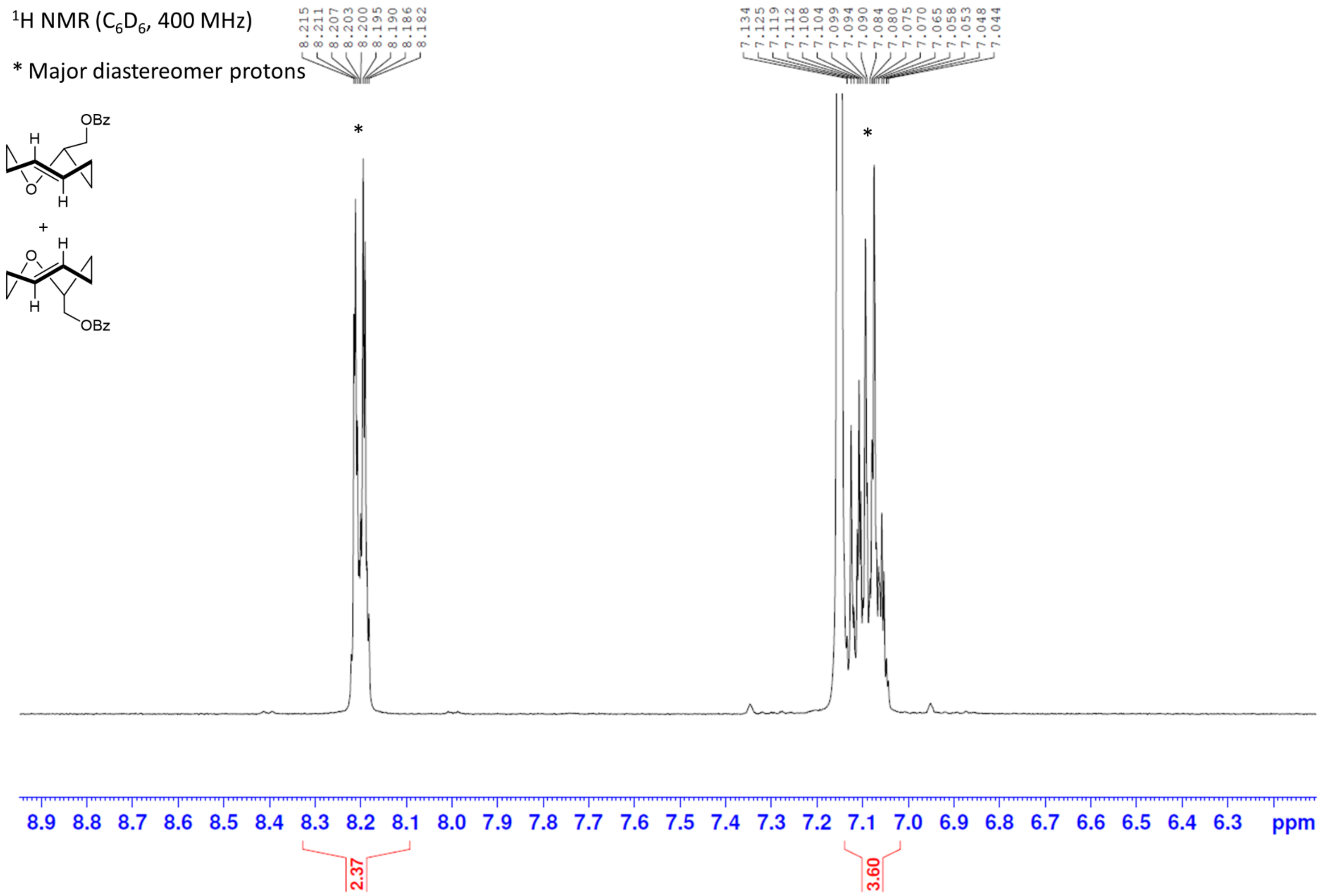

^{1}H NMR (C_6D_6, 400 MHz)
* Major diastereomer protons
OBz
H
O
+
8.215 8.211 8.207 8.203 8.200 8.195 8.190 8.186 8.182
7.134 7.125 7.119 7.112 7.108 7.104 7.099 7.094 7.090 7.084 7.080 7.075 7.070 7.065 7.058 7.053 7.048 7.044
*
*
8.9 8.8 8.7 8.6 8.5 8.4 8.3 8.2 8.1 8.0 7.9 7.8 7.7 7.6 7.5 7.4 7.3 7.2 7.1 7.0 6.9 6.8 6.7 6.6 6.5 6.4 6.3 ppm
2.37
3.60

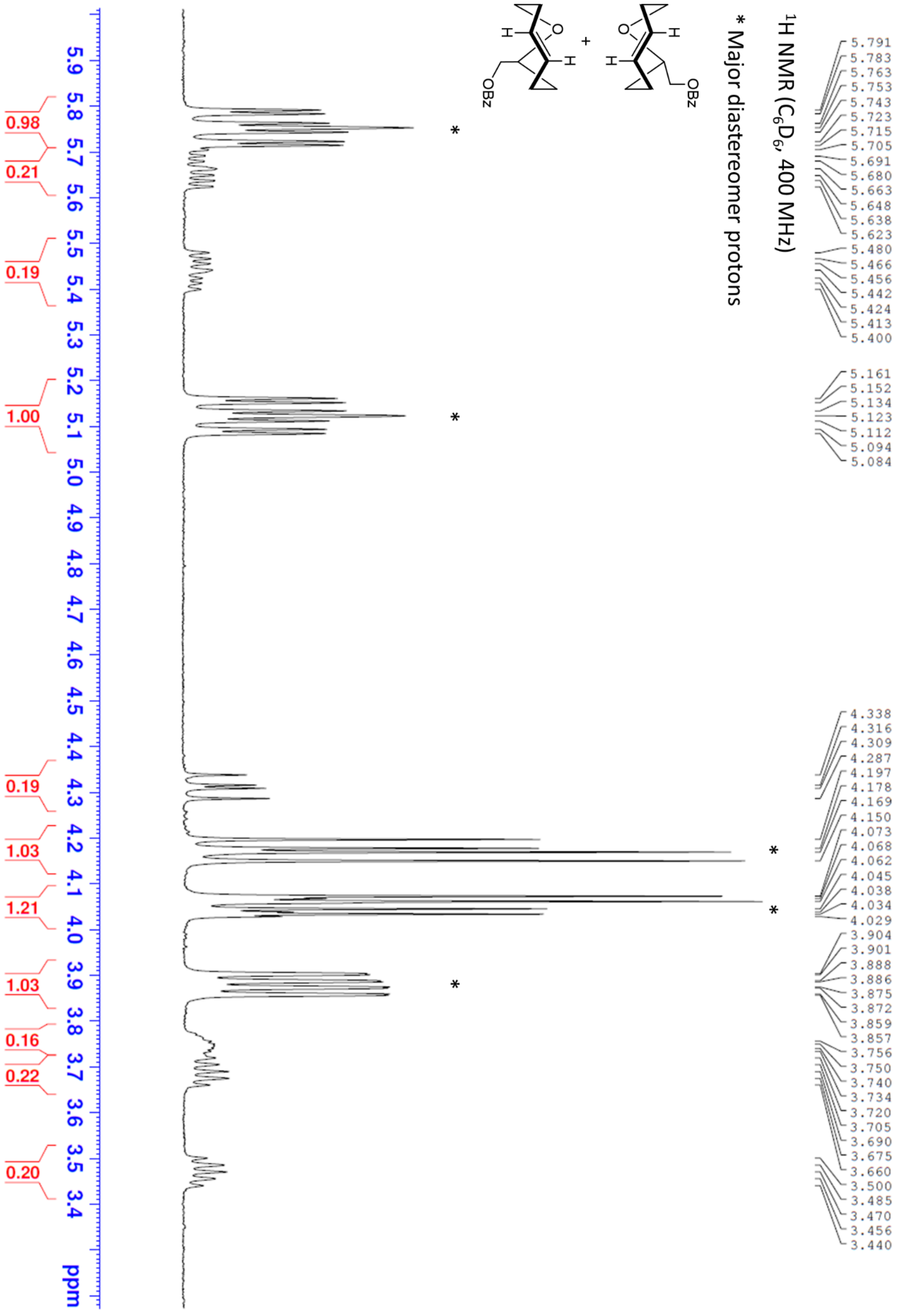

1H NMR (C6D6, 400 MHz)
* Major diastereomer protons
OBz
OBz
H
H
O
+
5.791 5.783 5.763 5.753 5.743 5.723 5.715 5.705 5.691 5.680 5.663 5.648 5.638 5.623 5.480 5.466 5.456 5.442 5.424 5.413 5.400
5.161 5.152 5.134 5.123 5.112 5.094 5.084
4.338 4.316 4.309 4.287 4.197 4.178 4.169 4.150 4.073 4.068 4.062 4.045 4.038 4.034 4.029 3.904 3.901 3.888 3.886 3.875 3.872 3.859 3.857 3.756 3.750 3.740 3.734 3.720 3.705 3.690 3.675 3.660 3.500 3.485 3.470 3.456 3.440
0.98 0.21 0.19 1.00 0.19 1.03 1.21 1.03 0.16 0.22 0.20
5.9 5.8 5.7 5.6 5.5 5.4 5.3 5.2 5.1 5.0 4.9 4.8 4.7 4.6 4.5 4.4 4.3 4.2 4.1 4.0 3.9 3.8 3.7 3.6 3.5 3.4 ppm

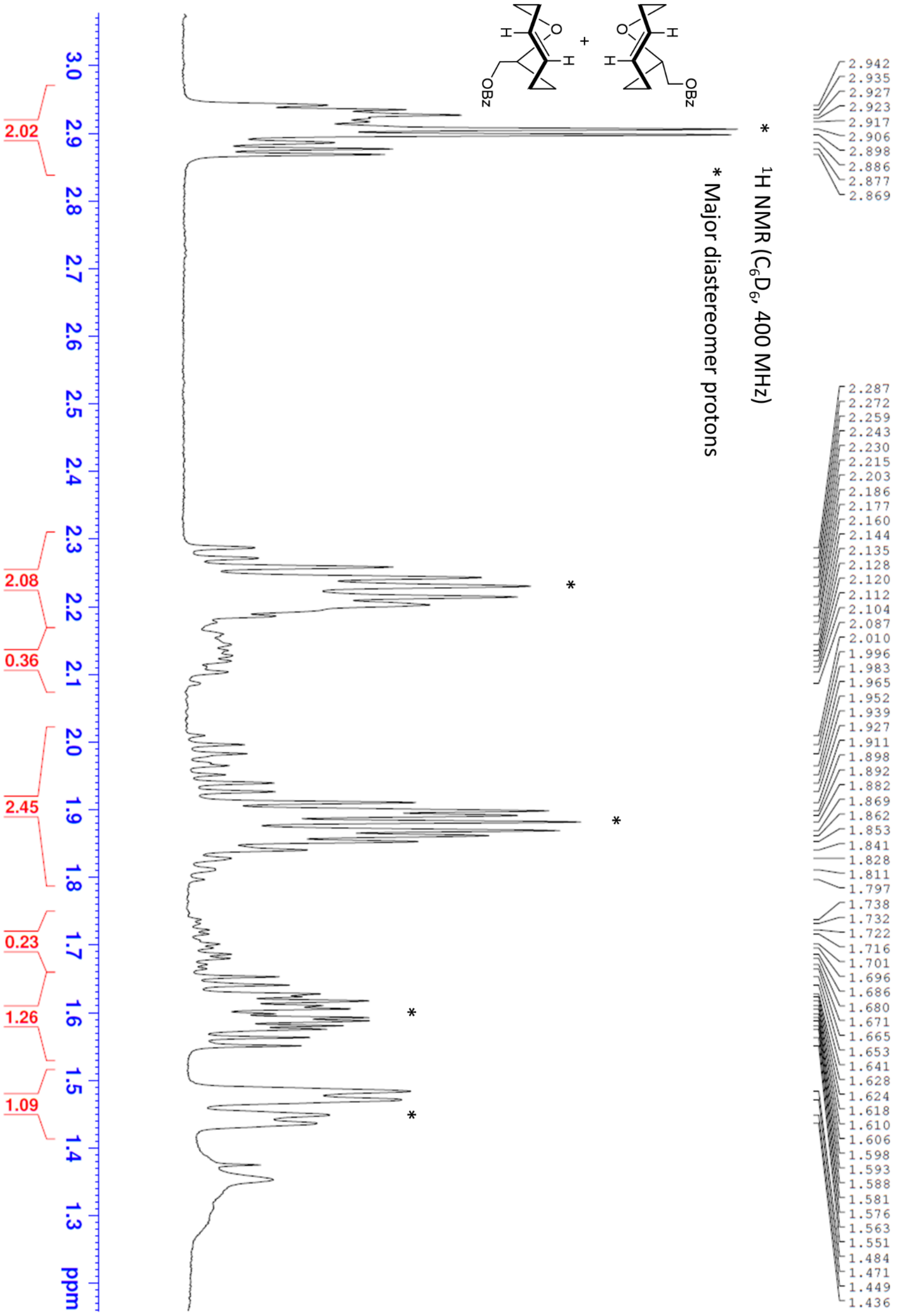
^{1}H NMR (C_6D_6, 400 MHz)
* Major diastereomer protons

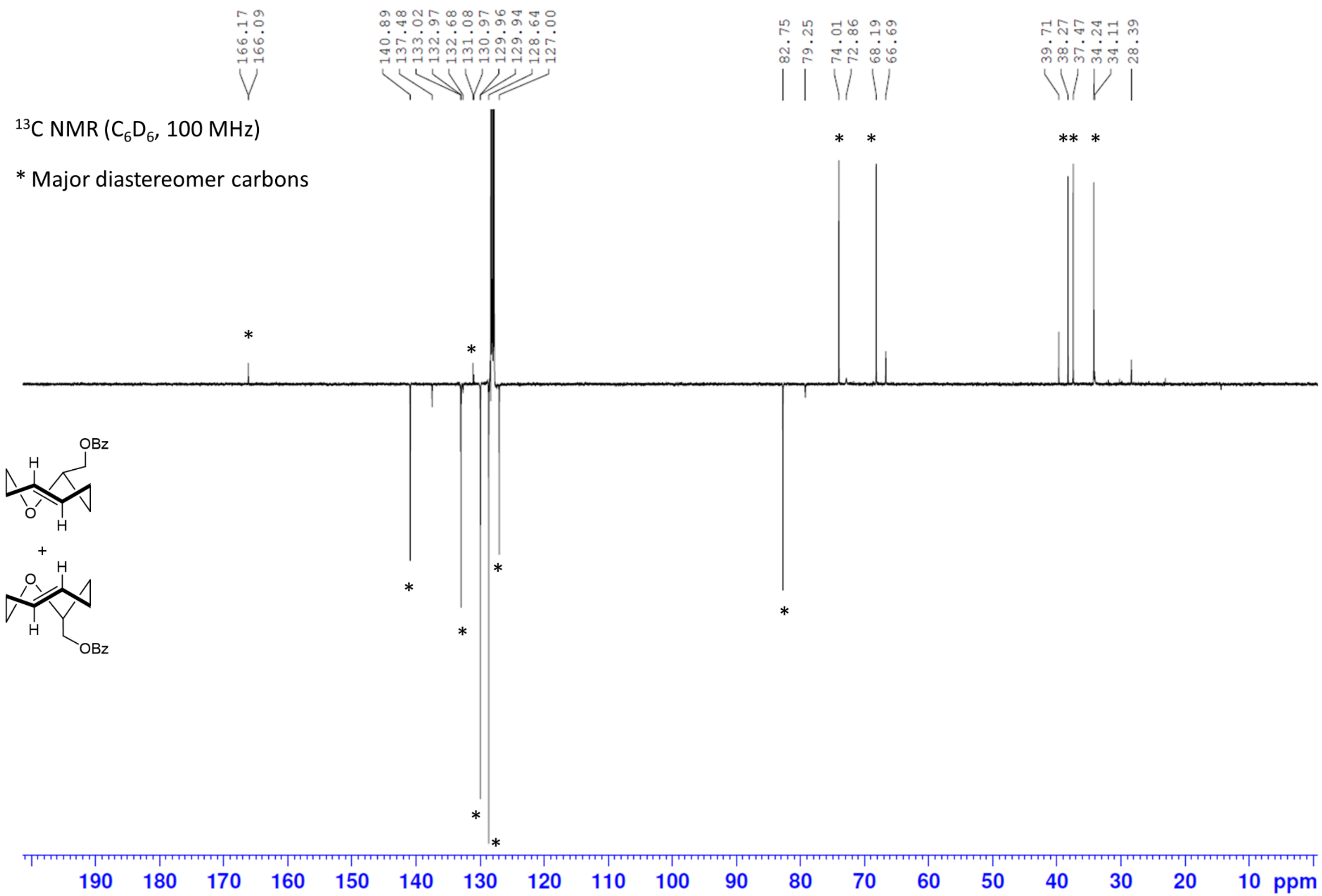

166.17
166.09
140.89
137.48
133.02
132.97
132.68
131.08
130.97
129.96
129.94
128.64
127.00
82.75
79.25
74.01
72.86
68.19
66.69
39.71
38.27
37.47
34.24
34.11
28.39
^{13}C NMR (C_6D_6, 100 MHz)
* Major diastereomer carbons
OBz
H
O
+
190 180 170 160 150 140 130 120 110 100 90 80 70 60 50 40 30 20 10 ppm

^1H NMR (C_6D_6, 400 MHz)

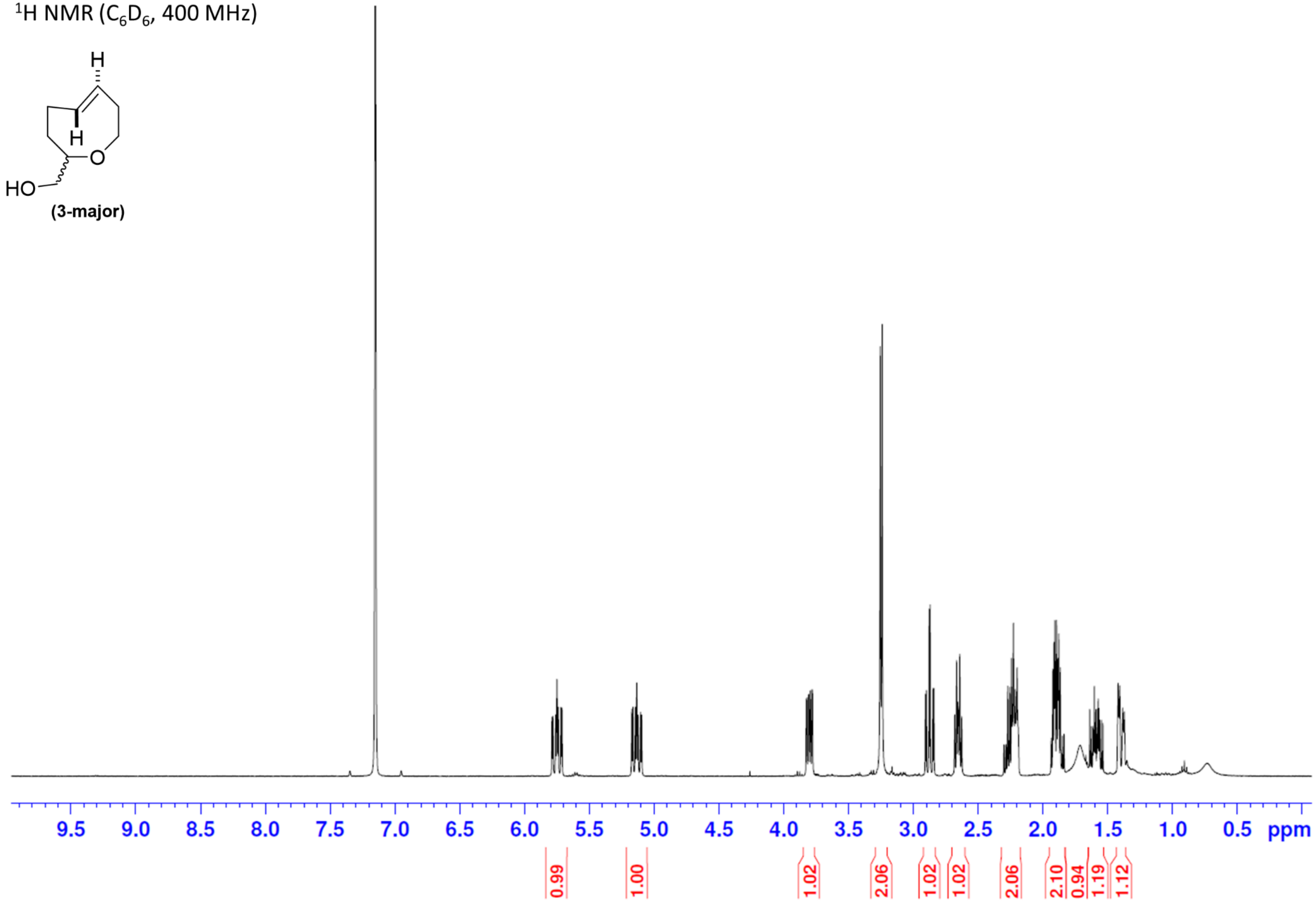

^1H NMR (C_6D_6, 400 MHz)

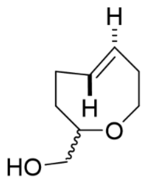

(3-major)

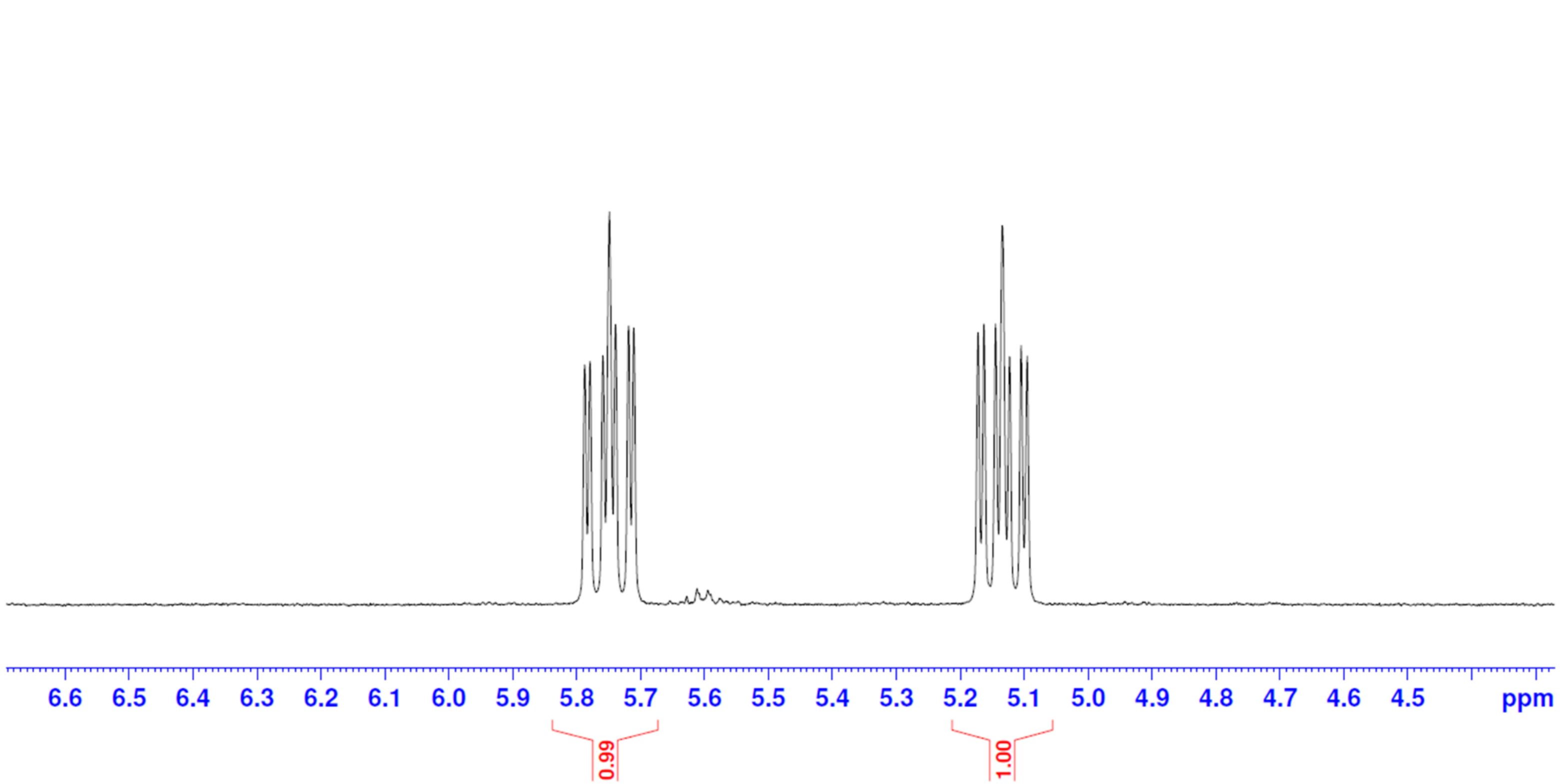

^{1}H NMR (C_6D_6, 400 MHz)

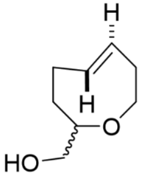

(3-major)

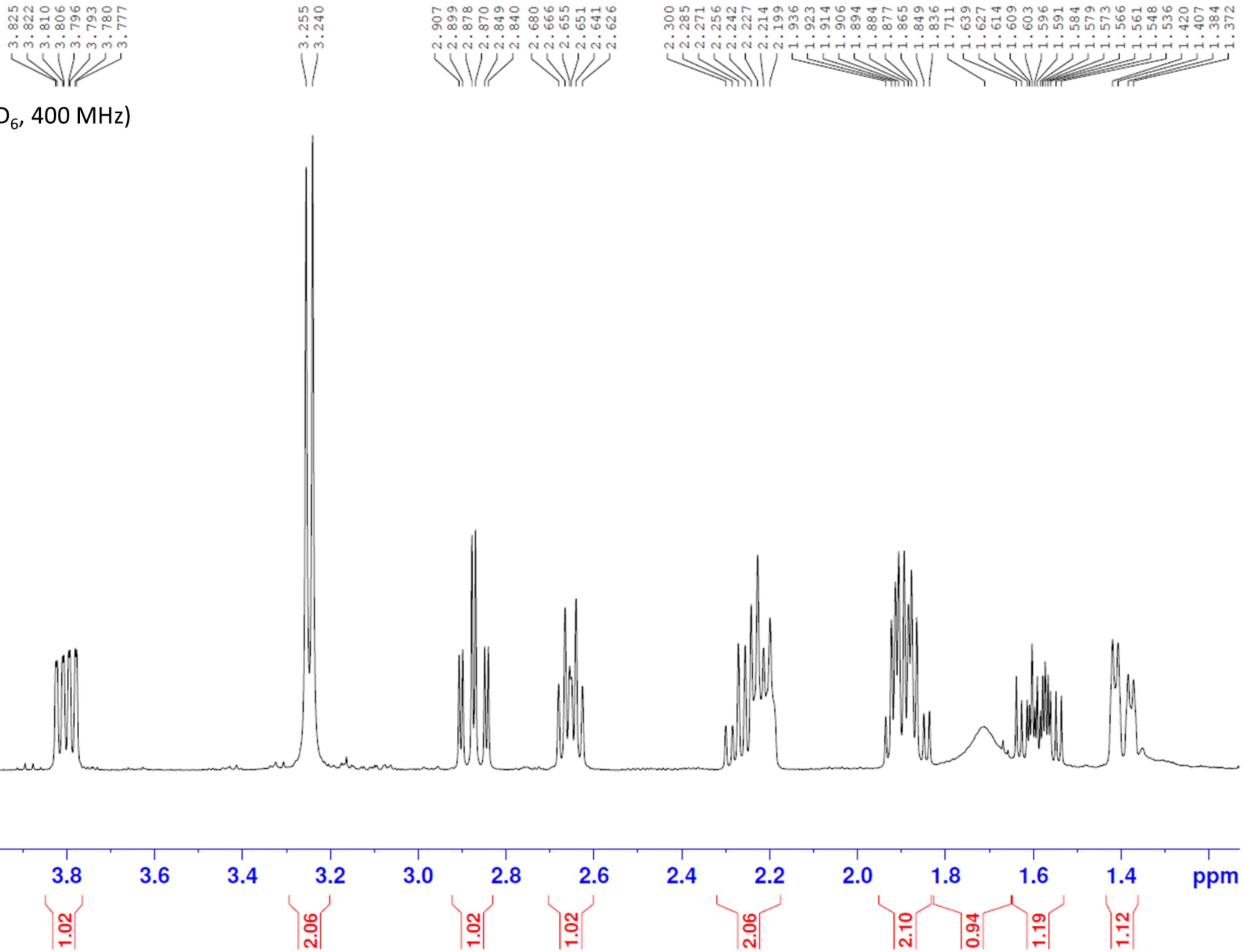

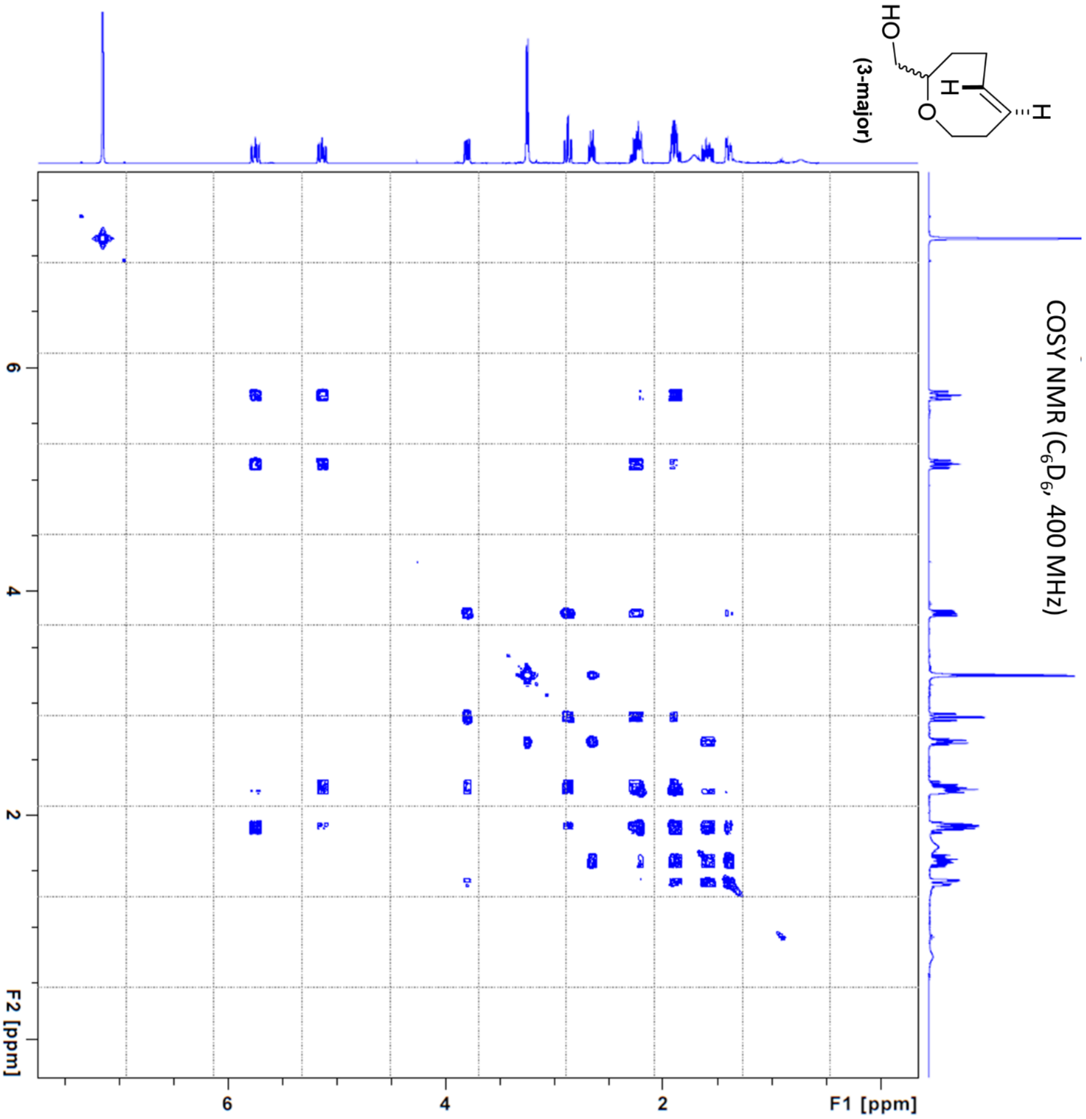
HO
H
O
H
(3-major)
COSY NMR (C_6D_6, 400 MHz)
F2 [ppm]
F1 [ppm]
6
4
2

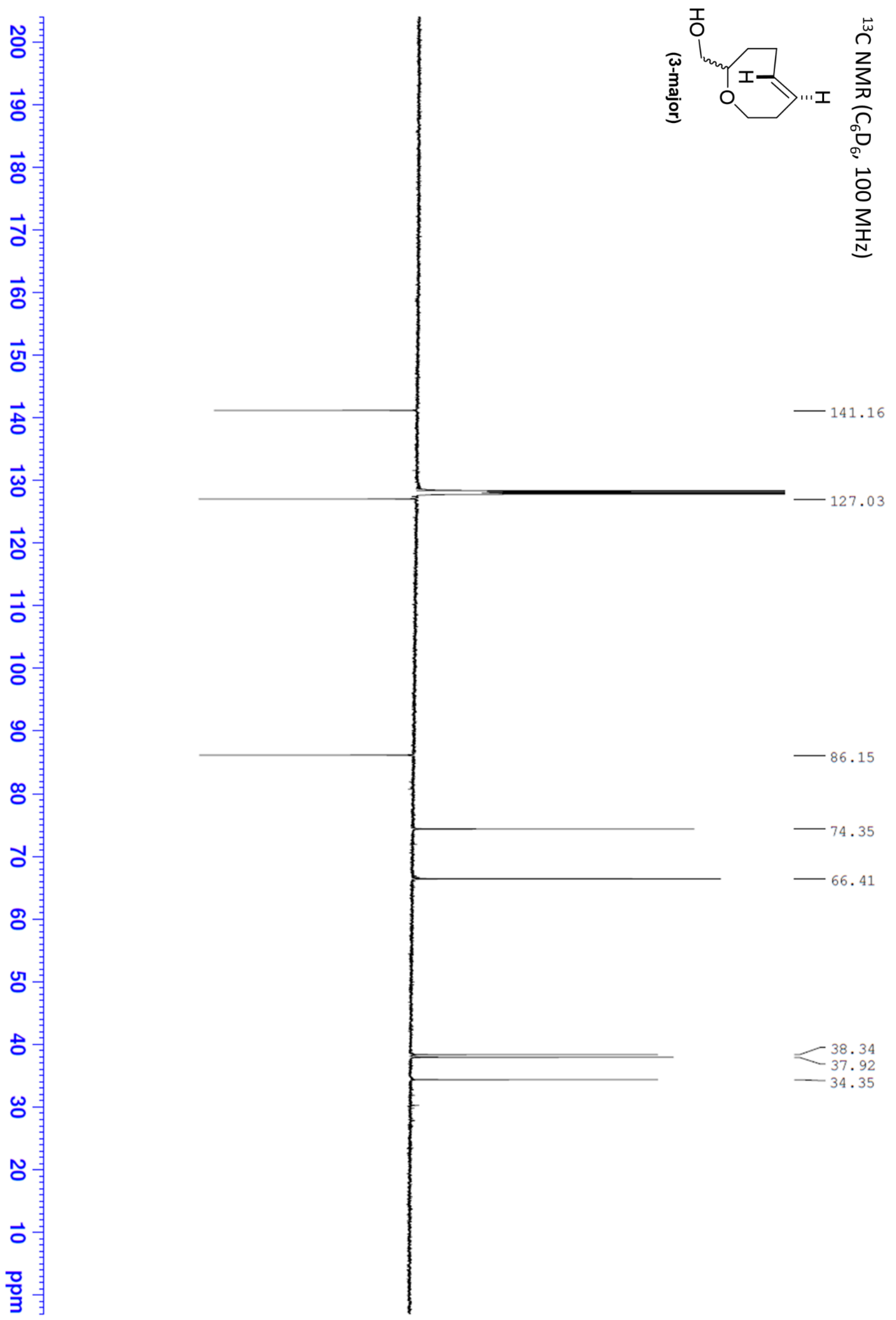

^{13}C NMR (C_6D_6, 100 MHz)
HO
H
O
H
(3-major)
141.16
127.03
86.15
74.35
66.41
38.34
37.92
34.35
200 190 180 170 160 150 140 130 120 110 100 90 80 70 60 50 40 30 20 10 ppm

S15. References

[1] P. K. Glasoe, F. A. Long; J. Phys. Chem. 1960, 64 (1), 188-190 DOI: 10.1021/j100830a521

[2] M. Royzen, G. P. A. Yap, J. M. Fox; J. Am. Chem. Soc. 2008, 130, 3760–3761 DOI: 10.1021/ja8001919

[3] R. J. Blizzard, D. R. Backus, W. Brown, C. G. Bazewicz, Y. Li, R. A. Mehl; J. Am. Chem. Soc. 2015, 137, 10044–10047 DOI: 10.1021/jacs.5b03275

[4] H. Zhang, W. S. Trout, S. Liu, G. A. Andrade, D. A. Hudson, S. L. Scinto, K. T. Dicker, Y. Li, N. Lazouski, J. Rosenthal, C. Thorpe, X. Jia, J. M. Fox; J. Am. Chem. Soc. 2016, 138, 5978–5983 DOI: 10.1021/jacs.6b02168
